# Latent causal diffusions for single-cell perturbation modeling

**DOI:** 10.1073/pnas.2602630123

**Published:** 2026-10-08

**Authors:** Lars Lorch, Jiaqi Zhang, Charlotte Bunne, Andreas Krause, Bernhard Schölkopf, Caroline Uhler

**Affiliations:** ^a^Department of Computer Science, Eidgenössische Technische Hochschule (ETH) Zürich, Zürich 8092, Switzerland; ^b^ Laboratory for Information and Decision Systems, Massachusetts Institute of Technology (MIT), Cambridge, MA 02139; ^c^ Eric and Wendy Schmidt Center, Broad Institute of MIT and Harvard, Cambridge, MA 02142; ^d^ School of Computer and Communication Sciences, École Polytechnique Fédérale de Lausanne (EPFL), Lausanne 1015, Switzerland; ^e^School of Life Sciences, Swiss Institute for Experimental Cancer Research, EPFL, Lausanne 1015, Switzerland; ^f^ Max Planck Institute for Intelligent Systems, Tübingen 72076, Germany; ^g^European Laboratory for Learning and Intelligent Systems (ELLIS) Institute, Tübingen 72076, Germany

**Keywords:** diffusion model, causal inference, perturbation modeling, single-cell analysis, interpretability

## Abstract

Perturbation screens hold the potential to systematically map regulatory processes at single-cell resolution, yet modeling and predicting transcriptome-wide responses to perturbations remains a major computational challenge. Existing methods often underperform simple baselines, fail to disentangle measurement noise from biological signal, and provide limited insight into the causal structure governing cellular responses. Here, we present the latent causal diffusion (LCD), a generative model that frames single-cell gene expression as a stationary diffusion process observed under measurement noise. LCD outperforms established approaches in predicting the distributional shifts of unseen perturbation combinations in single-cell RNA-sequencing screens while simultaneously learning a mechanistic dynamical system of gene regulation. To interpret these learned dynamics, we develop an approach we call causal linearization via perturbation responses (CLIPR), which yields an approximation of the direct causal effects between all genes modeled by the diffusion. CLIPR provably identifies causal effects under a linear drift assumption and recovers causal structure in both simulated systems and a genome-wide perturbation screen, where it clusters genes into coherent functional modules and resolves causal relationships that standard differential expression analysis cannot. The LCD-CLIPR framework bridges generative modeling with causal inference to predict unseen perturbation effects and map the underlying regulatory mechanisms of the transcriptome.

## Significance

Mapping the gene regulatory networks that govern cellular behavior is fundamental to understanding biology. While modern perturbation experiments reveal how specific genes influence one another, the exponential number of possible gene combinations makes exhaustive experimental testing impossible. We therefore require computational models that can both predict responses to unseen combinatorial perturbations and explain their underlying causal mechanisms. We introduce a diffusion-based generative model that learns explicit gene-to-gene regulation directly from perturbation data. Our approach improves the prediction of combinatorial perturbation effects and identifies causal links that standard statistical comparisons miss.

Genome-wide perturbation screens now enable causal interrogation of gene function at single-cell resolution (1–3). As these screens scale to capture the transcriptomic effects of thousands of individual gene perturbations, the central challenge shifts from data collection to modeling regulatory interactions and predicting the outcomes of multigene combinations. Genetic interactions are often nonlinear, with the effects of combined perturbations deviating from the sum of their individual effects (4, 5). Because the combinatorial search space grows exponentially, exhaustive experimental assays quickly become infeasible (6, 7). Mapping the genetic circuitry of cellular programs thus requires computational causal models that can generalize from observed data to predict the effects of unseen perturbation combinations and reveal the mechanisms governing their interactions (4, 8–11).

While deep learning has advanced representation learning for single-cell biology (12), current models often struggle to outperform simple, domain-aware baselines when predicting postperturbation responses (13–15). Foundation models pretrained on large single-cell atlases, for instance, frequently fail to predict gene expression changes for held-out perturbations more accurately than additive heuristics (16–18). Neural network models trained on individual perturbation screens typically focus on average effects, neglecting the heterogeneity and stochasticity that define cellular states (17, 19). Furthermore, these models rarely account for measurement noise, often conflating technical artifacts with biological signal (20–22). Above all, these architectures remain largely opaque, failing to characterize how perturbations propagate through genetic pathways to drive transcriptional changes. Consequently, current methods provide limited structural insight into the causal mechanisms that govern cellular responses.

In this work, we introduce single-cell perturbation modeling based on the latent causal diffusion (LCD), a generative model that frames gene expression as a latent stationary dynamical system observed under measurement noise (23, 24). We formalize gene regulation as a diffusion process governed by a system of stochastic differential equations (SDEs), where each gene’s expression evolves mechanistically as a function of its regulators. Assuming this diffusion process operates in equilibrium (25–28), we infer the functional causal mechanisms of the SDEs without direct observation of the temporal dynamics, viewing single-cell data as samples from the stationary distribution under a measurement model (29–31). Unlike previous deep learning approaches, this enables identifying causal data-generating mechanisms while explicitly decoupling biological from technical noise. We model perturbations of the transcriptome as modifications to the underlying gene regulatory mechanisms, causing the diffusion to reach a different equilibrium. When applied to large-scale Perturb-seq screens (5, 6), LCD predicted the distributional shifts of unseen perturbation combinations more accurately than established approaches while learning an explicit, gene-level dynamical system as the generative model.

The regulatory dynamics learned by LCDs are parameterized by neural networks. To render their causal dependencies interpretable, we develop causal linearization via perturbation responses (CLIPR), a linearization technique that approximates the direct gene–gene causal effects modeled in the diffusion process. For linear systems, CLIPR provably identifies causal effects by leveraging how drift perturbations behave at a control state and in equilibrium. In nonlinear systems, it provides the minimum-norm linear causal effects that best approximate the drift’s response to perturbations. We validated CLIPR on simulated systems, demonstrating that it reliably recovers ground-truth causal structures from complex neural network drifts. Finally, by applying LCD and CLIPR to a genome-wide Perturb-seq assay (1), we uncovered regulatory mechanisms in K562 cells that remain hidden under standard differential expression analysis, providing a scalable framework for mapping causal dependencies in the cell.

## Results

### A Causal Generative Model of the Transcriptome.

Latent causal diffusions model the joint expression of d genes in a cell as a stochastic process $x(t)\in \;R^{d}$ evolving over time t, as gene expression is inherently stochastic (25, 32, 33). Each gene g is represented by a random state $x_{g}\left(t\right)$, corresponding to a noiseless, unobserved version of expression, that evolves according to a stochastic differential equation (SDE) (34)

**Fig. 1.**
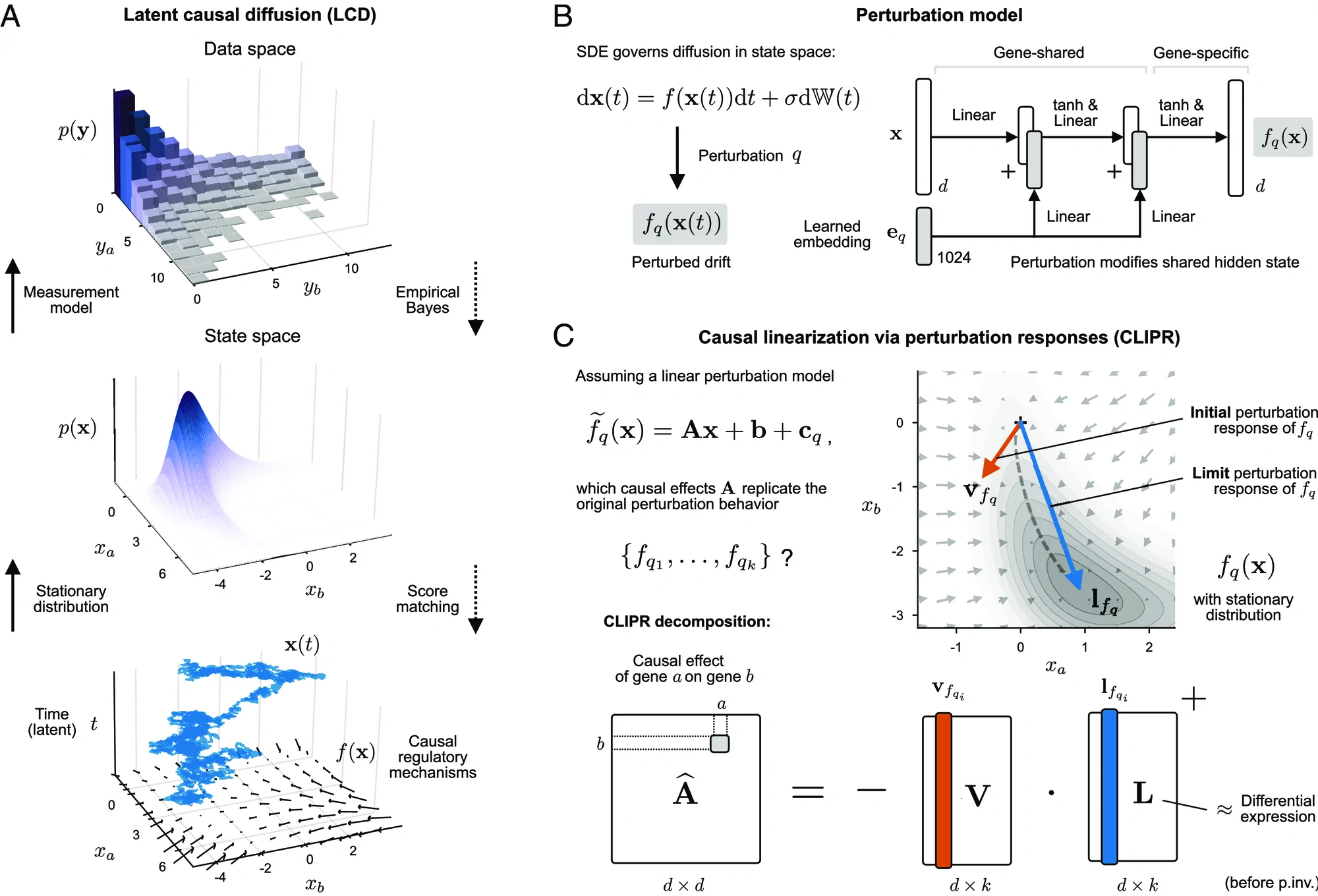
The latent causal diffusion (LCD) model and CLIPR. (*A*) LCDs model single-cell expression data y (*Top*) as noisy observations of unobserved, noiseless gene states x (*Center*). States x are samples from a stationary stochastic process x(t) evolving under the causal regulatory dynamics f(x) of an SDE (*Bottom*). To train LCDs, we first infer p(x) via empirical Bayes, then learn f in state space via score matching. (*B*) Dynamics f explicitly model gene regulation and are parameterized by a neural network with shared hidden state across genes. Perturbations modify this hidden state via learned embeddings $e_{q}$. (*C*) To interpret the causal dependencies in f, we estimate a linear causal matrix that induces the perturbation behavior of f (*Left*). This matrix is computed from the initial ($v_{f_{q}}$) and limit ($\ell_{f_{q}}$) responses when f is perturbed. The limit response is approximated by following the vector field flow until convergence (*Right*).

[1] $$\begin{array}{cc} \text{d}x_{g}(t) & ={\left[f(x(t))\right]}_{g}\text{d}t+\sigma {\text{d}W}_{g}(t), \end{array}$$

where $W_{g}(t)$ denotes standard Brownian motion. The drift function $f:R^{d}\to R^{d}$ models the causal regulatory mechanisms among the genes, and the diffusion term captures stochastic fluctuations with scale σ. The joint stochastic process x(t), obtained by solving the coupled SDEs for all genes, defines the distribution over cell states at time t (Fig. 1*A*, *Bottom*).

LCDs model the process x(t) as stationary, so the SDEs induce a time-independent distribution p(x) over gene states. The stationarity assumption enables inference of the causal dynamics f directly from the state distribution p(x), without requiring time-resolved observations of x(t) itself (Fig. 1*A*, *Center*) (23, 31). The time dimension of LCDs allows modeling gene feedback loops by implicitly unrolling causal effects over time, akin to biological systems (35–37). This concept of time differs from recent diffusion-based models, which either model time series (38, 39) or treat time as computation steps rather than a physical timescale of gene regulation (40, 41). We provide additional intuition on stationarity and the causal effects encoded in the drift f in *SI Appendix*.

Our model assumes that perturbational measurements are collected at a time point of the experiment when gene expression profiles have approximately reached a steady state. Although some genes, such as those involved in cell cycle or stress responses, exhibit intrinsically dynamic behavior, this limitation is not unique to perturbational settings and similarly affects observational settings, where steady-state models are canonically used as mathematical descriptions of gene expression (25–28). We expect this abstraction to be most appropriate for perturbation data measured at a single time point and for relatively stable genes that dominate biologically meaningful variation and regulatory responses.

We parameterize the drift f by a neural network that shares parameters across the causal mechanisms of each gene (Fig. 1*B*). Specifically, ${\left[f\left(x\right)\right]}_{g}$ and ${\left[f\left(x\right)\right]}_{g^{\prime }}$ share a hidden state, reflecting the substantial structure of gene regulatory circuits (42–45). In our experiments, the shared hidden dynamics enabled LCDs to scale to modeling a thousand genes jointly. We model perturbations as shifts in this hidden state, computed from learned embedding vectors $e_{q}$, where q is the index of a perturbation (Fig. 1*B*), as gene expressions tend to covary across perturbations, and perturbations cluster by their shared transcriptomic effects (42, 43, 46). Combinations of perturbations are represented as additive compositions of these embeddings. Perturbations thus act on the regulatory mechanisms of f rather than directly on gene expression, forcing the diffusion to converge to a new steady state. We write $f_{q}$ to denote the dynamics f under perturbation q.

We do not observe the state distribution p(x) directly but through noisy measurements of single cells. LCDs model each single-cell count vector $y\in N^{d}$ as an independent sample from the latent stationary diffusion, observed under a zero-inflated Poisson (ZIP) likelihood (30, 31)

[2] $$\begin{array}{cc} p(y_{g}|x_{g};\;\pi_{g}) & =\;\text{ZIP}(y_{g};\;\mu_{g}\left(x_{g}\right),\pi_{g}) \end{array}$$

with dropout probability $\pi_{g}$. Here, $\mu_{g}:R\to R^{+}$ is a link function with fixed, gene-specific scaling that maps states $x_{g}$ to positive Poisson rates. If the Poisson rate follows a Gamma distribution, the distribution of $y_{g}$ has a zero-inflated negative binomial form, which corresponds to the steady-state of the promoter-activation model commonly characterizing scRNA-seq data (28, 29, 47). LCDs generalize this to richer distributions p(y) by capturing causal dependencies among genes in p(x) (Fig. 1*A*, *Top*).

To infer the model from data, we view a perturbation screen as a collection of count distributions, one per perturbation $q_{i}$, where $q_{0}$ represents the unperturbed setting ($f_{q_{0}}=f$). Each count distribution arises from the same drift and measurement model, differing only by the perturbation-specific shifts in the hidden state of $f_{q_{i}}$ (31). To separate measurement and biological noise, we first infer the likelihood parameters π and state distribution p(x) for each perturbation condition. We then jointly fit the dynamics f and perturbation embeddings $e_{q}$ to all inferred state distributions (*Materials and Methods*).

### Estimating Causal Dependencies in Stationary Diffusions.

A learned LCD models a causal generative process, in which genes mechanistically regulate one another, but the dynamics of LCDs are not directly interpretable, since the drift f(x(t)) is nonlinear and depends on x(t). However, perturbations of the drift $\left\{f_{q_{1}},\cdots ,f_{q_{k}}\right\}$ reveal key regulatory mechanisms modeled by f. We can leverage this perturbation behavior to obtain a linearization of the system that preserves its overall perturbation responses, enabling us to interpret the causal structure of a learned LCD drift f.

The key idea is to ask: What linear drift $\tilde{f}$ would replicate the perturbation behavior of $\left\{f_{q_{1}},\cdots ,f_{q_{k}}\right\}$ (Fig. 1*C*, *Left*)? We show that such a linear approximation can be estimated in closed form from simple properties of the perturbed drifts $f_{q_{i}}$. Since linear systems describe gene–gene regulation by a single parameter per pair, they yield an interpretable approximation of the regulatory dynamics of f. We consider linear SDEs (Eq. **1**) with drift

[3] $$\begin{array}{cc} {\tilde{f}}_{q}\left(x\right) & =Ax+b+c_{q}, \end{array}$$

where $A\in R^{d\times d}$ and $b\in R^{d}$ are causal effect and bias parameters, $c_{q}\in R^{d}$ model the perturbations $q\in \left\{q_{1},\cdots ,q_{k}\right\}$, and the real part of the maximum eigenvalue of A is strictly negative. The matrix A and bias b are shared across perturbations and encode the gene–gene regulatory effects, whereas $c_{q}$ is perturbation-specific and encodes how each perturbation q targets the genes. Linear SDEs of this form have a unique Gaussian stationary distribution with mean (48)

[4] $$\begin{array}{c} \mu_{q}=-A^{-1}\left(b+c_{q}\right). \end{array}$$

We can describe the perturbation $f_{q}$ of a drift f by two intuitive properties: the drift vector acting at a control state and the steady state it induces. To formalize these properties, we use the zero vector 0 as the control state, which represents the mean of a standardized, unperturbed linear system. We then define the *initial perturbation response* of $f_{q}$ as

[5] $$\begin{array}{c} v_{f_{q}}:=f_{q}\left(0\right), \end{array}$$

and the *limit perturbation response* of $f_{q}$ as the fixed point

[6] $$\begin{array}{cc} \ell_{f_{q}} & :=x^{*}\;\;\text{s.t.}\;f_{q}\left(x^{*}\right)=0. \end{array}$$

For the linear model, the postperturbation mean $\mu_{q}$ is the fixed point $\ell_{f_{q}}$. Therefore, the limit perturbation response $\ell_{f_{q}}$ directly corresponds to differential expression, assuming the unperturbed system has mean 0. By contrast, the initial perturbation response $v_{f_{q}}$ represents the initial drift toward the postperturbation steady state (Fig. 1*C*, *Right*). We assume that a fixed point exists and can be approximated by integrating the drift flow (*Materials and Methods*).

Applying different perturbations $q_{i}$ to a diffusion drift f yields different perturbation responses $v_{f_{q_{i}}}$ and $\ell_{f_{q_{i}}}$. Each respective perturbation outcome reveals key drivers of the dynamics f, and collectively they map how the system behaves under perturbations. Our theorem below shows that, given several perturbation response vectors, we can construct a linear causal effect matrix $\hat{A}$ that replicates the same response vectors as the original perturbed drifts. Moreover, assuming the true drift is linear, sufficiently diverse perturbations identify all causal effects:

Theorem 1. *Let* $v_{f_{q_{i}}}$ *and* $\ell_{f_{q_{i}}}$ *be perturbation responses of a general drift* $f_{q}$ *for perturbations* $q_{i}\in \left\{q_{1},\cdots ,q_{k}\right\}$. *The matrix of the linear drift (Eq. **3**) that approximates these perturbation response vectors with least-squares error and minimum Frobenius norm is given by*

[7] $$\begin{array}{c} \hat{A}=-VL^{+}, \end{array}$$

*where* ${\left(\cdot \right)}^{+}$ *denotes the Moore–Penrose pseudoinverse, and* $V,L\in R^{d\times k}$ *are matrices with* i*-th columns* $v_{f_{q_{i}}}$ *and* $\ell_{f_{q_{i}}}$*, respectively. Moreover, if the drift function is linear (*$f_{q}={\tilde{f}}_{q}$*) and* L *has full row rank, then* $\hat{A}=A$.

Our proof is given in *SI Appendix*. When the bias b is generic, a sufficient condition for L being full rank is that k≥d perturbation shift vectors $c_{q}$ are linearly independent. We refer to this system as the causal linearization via perturbation responses (CLIPR). In practice, we use a Tikhonov-regularized variant, which improves stability under model mismatch by trading off perturbation response fit against smaller norms of the causal effects A via a scalar λ (see proof of *Theorem* 1):

[8] $$\begin{array}{c} {\hat{A}}_{\lambda }=-VL^{⊤}(LL^{⊤}+\lambda I{)}^{-1}. \end{array}$$

*Theorem* 1 has several implications. First, the identification result suggests that all single-gene perturbations can fully disentangle the causal effects in a linear system. Specifically, *Theorem* 1 shows that linear causal effects can be directly decomposed into (and uniquely identified by) the DEs L and the drift forces V at the control state 0 (Fig. 1*C*, *Right*). While access to exact perturbation responses constitutes an idealized setting, the result provides a theoretical foundation for estimating A by computing $v_{f_{q}}$ and $\ell_{f_{q}}$ for drifts $f_{q}$ learned from finite samples and noisy observations.

Second, the simple form $\hat{A}=-VL^{+}$ highlights a fundamental distinction between direct causal effects and differential expression. Under the simplifying assumptions discussed above, differential expression (DE), the mean change in expression induced by a perturbation, corresponds to the limit perturbation responses L. CLIPR shows that DE, as captured by L, is distinct from the direct causal effects among the genes themselves: DE emerges from unrolling direct causal effects A over time. We say that gene a is a direct cause of gene b if b depends on perturbations of a when all other genes are held fixed (49). By definition of the drift (Eq. **3**), A encodes precisely these direct causal dependencies.

Third, CLIPR enables interpreting nonlinear drifts $f_{q}$, since $v_{f_{q}}$ and $\ell_{f_{q}}$ can be computed for any drift function that models perturbations. CLIPR then reveals the linear causal effects $\hat{A}$ that best approximate the perturbation behavior of the original dynamics $f_{q}$. The regularized estimator ${\hat{A}}_{\lambda }$ mitigates artifacts resulting from model mismatch when the linear model is overdetermined by the nonlinear responses, since it encourages smaller norms of A in favor of overfitting the responses. However, as ${\hat{A}}_{\lambda }$ is shrunk toward zero along directions weakly spanned by the columns of L, small entries should not be read as evidence of absent regulation (*SI Appendix*, Fig. S13).

Fourth, the perturbation shifts $c_{q}$ in the approximated linear drift (Eq. **3**) are not needed to compute the estimate $\hat{A}$. What matters is that the original drift $f_{q}\left(x\right)$ can be evaluated and integrated to obtain V and L. Consequently, perturbations in the approximated linear drift (Eq. **3**) do not require known targets and are not restricted to gene perturbations. More generally, the LCD-CLIPR framework is agnostic to perturbation type and could therefore also be applied to perturbations induced by drugs or other chemical compounds. In fact, the following corollary shows that the perturbation shifts $c_{q}$ in the approximated linear model are implied by the CLIPR matrix $\hat{A}$:

Corollary 2. *The bias* $\hat{b}$ *and shift vectors* ${\hat{c}}_{q}$ *of the linear model (Eq. **3**) implied by the matrix* $\hat{A}$ *of Theorem 1 are*

b^=−A^ℓf  and  c^q=A^ℓf−A^ℓfq.

### Perturbation Effect Prediction at Single-Cell Resolution.

We evaluated LCDs against established methods for predicting the effects of unseen perturbation combinations in Perturb-seq assays. Our evaluation used two datasets that contain both single-gene and double-gene perturbations, spanning different cell lines and CRISPR-Cas technologies [Norman et al. (5) and Wessels et al. (6); *SI Appendix*, Table S1]. For both datasets, each method was trained on control samples, all single-gene perturbations, and a subset of double-gene perturbations. After training, methods predicted full single-cell distributions of the transcriptome for the held-out two-gene combinations. Using ten disjoint testing folds, we obtained held-out predictions for all two-gene perturbations in both screens (*SI Appendix*, Fig. S2).

We compared LCDs to various methods. Recent findings suggested that established approaches perform on par with additive heuristics (13, 14, 50). We thus evaluated an additive baseline called SALT and its variant PEPER, which learns a nonlinear correction on top of SALT (15). We also evaluated the deep-learning approaches CPA (21), which uses autoencoders, and GEARS (19), which learns graph neural networks over gene relationship graphs sourced from prior knowledge (*Materials and Methods*). We quantified prediction accuracy relative to the observed postperturbation data using several metrics. Our primary metric was the maximum mean discrepancy (MMD), which measures full distributional fit (20, 51). Low MMD implies the prediction matches all moments of the observed single-cell distribution, characterizing not only its means, but also its (co)variances, skewness, and tail-heaviness. Following prior work, we also report the root mean squared error (RMSE) between predicted and observed means (19, 21) as well as the Pearson correlation of expression changes relative to the average of all observed perturbation means (52). We mostly focused on metrics computed on the top 20 differentially expressed genes, as most genes often show negligible change under perturbation (19, 20).

**Fig. 2.**
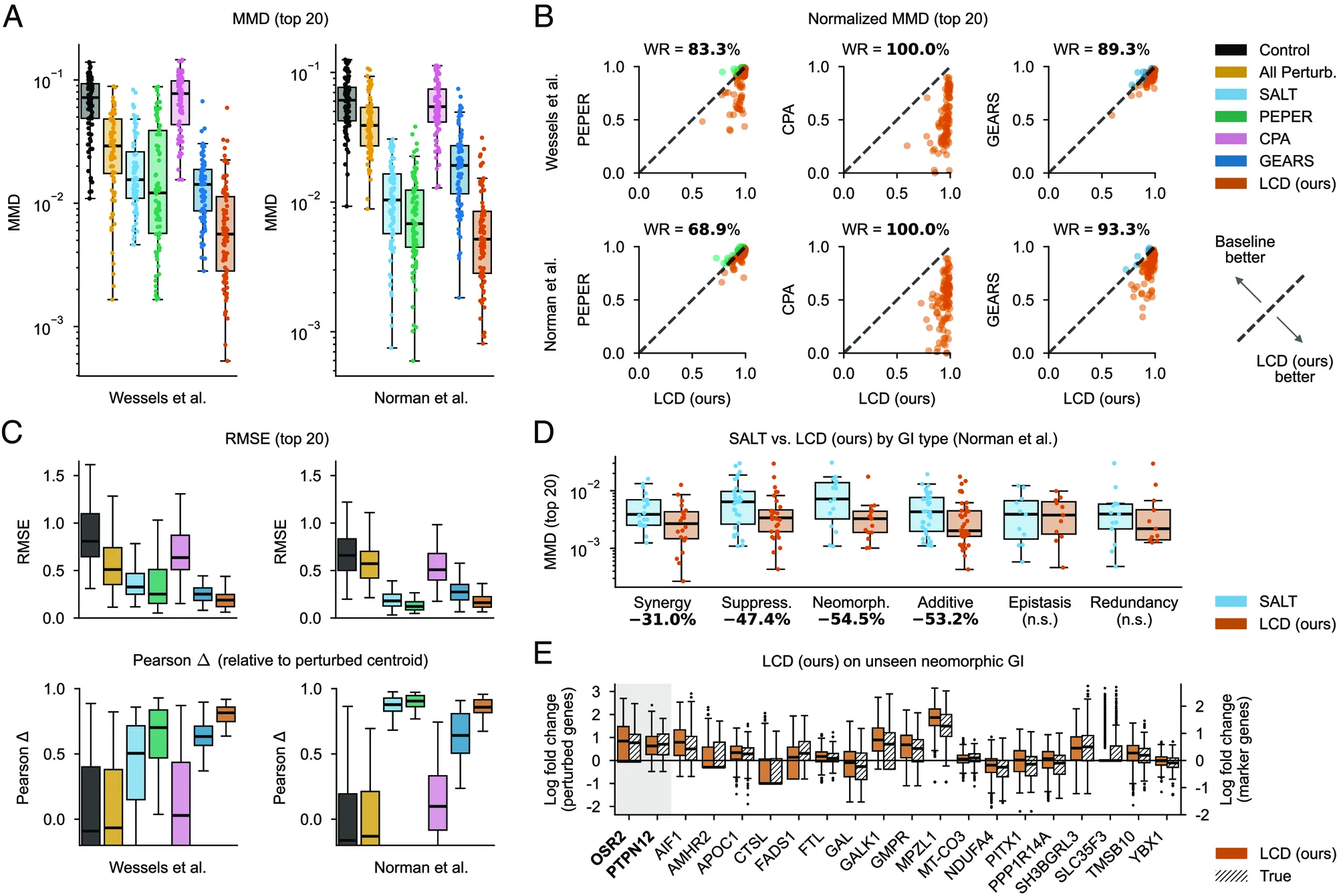
LCDs outperform state-of-the-art models at predicting combinatorial perturbation effects. (*A*) Maximum mean discrepancy (MMD) between predicted and observed single-cell expressions for held-out double-gene perturbations of the two datasets, computed on top 20 differentially expressed (DE) genes. Each dot represents one perturbation. Two leftmost bars show baseline performance of randomly sampled control and perturbed cells from all conditions. (*B*) Per-perturbation comparison of LCD with deep-learning baselines on MMD, normalized to [0,1] across methods. Win rate (WR): proportion of perturbations where LCD achieved lower error. (*C*) Predicted vs. observed mean expression. RMSE over top 20 DE genes (*Top*) and Pearson correlation of changes relative to perturbed centroid (average of all observed perturbation means) (*Bottom*). (*D*) MMD stratified by genetic interaction (GI) type, comparing LCD and the additive baseline SALT. Axis labels indicate significant improvement per GI category (percentage reduction in median MMD by LCD) or not (n.s.; one-sided Wilcoxon signed-rank test, P<0.05). (*E*) Predicted vs. observed expression for top 20 DE genes of a held-out perturbation with neomorphic GI. Gray section shows the perturbed genes (bold labels). All box plots show the median, interquartile range (IQR), and whiskers extend to farthest points within 1.5× IQR.

LCDs significantly outperformed both the heuristics and established approaches in terms of overall distributional accuracy of the predicted single-cell transcriptomes (Fig. 2*A*). Per-perturbation comparisons on each dataset show that LCDs made more accurate predictions as measured by MMD than all other methods in more than roughly 80% of test perturbations (Fig. 2*B*). Only PEPER achieved comparable performance to LCDs on the screen by Norman et al., where more single-gene perturbations are available for calibrating its additive heuristic (98 for Norman et al. vs. 5 for Wessels et al.; *SI Appendix*, Table S1), suggesting that LCDs disentangle indi vidual gene contributions effectively from combined perturba tions. When reducing the single-cell transcriptome to a mean expression vector, LCDs still provided the most accurate predictions across all baselines, though SALT and PEPER performed more competitively (Fig. 2*C*, *Top*). Its advantage over the baselines was most pronounced when adjusting for systematic variation under perturbation (Fig. 2*C*, *Bottom*). LCDs thus achieved significant accuracy gains by modeling a full single-cell count distribution and produced the most perturbation-specific predictions after accounting for systematic variation. These results held when stratifying by gene activity level (*SI Appendix*, Fig. S6) and on the simpler task of classifying which genes are most differentially expressed, where LCDs achieved the highest precision (*SI Appendix*, Fig. S7).

Combinations of gene perturbations may behave differently than their individual components suggest. We followed Norman et al. (5) and classified double-gene perturbations into six genetic interaction (GI) types based on their observed transcriptomic profiles, stratifying the performance of LCDs by GI type (*SI Appendix*). By comparing to the naïve additive SALT heuristic, we isolated the GI types for which LCD yielded significant improvement (Fig. 2*D*). LCDs improved most over an additive model, for example, for unexpected (neomorphic) GIs, where a linear model of the individual effects does not provide a good fit for the combination profile. By contrast, SALT performed on par with LCDs for redundant or epistatic GIs, where either both or one of the individual perturbations, respectively, strongly explain the combined effect. While both methods use additivity to model combinations, they differ in where this addition occurs: SALT naïvely sums differential expression profiles, whereas LCD sums perturbation embeddings $e_{q}$ that modulate the hidden state of a nonlinear drift function (Fig. 1*A* and *B*). This allows LCDs to capture interaction effects that unroll through the nonlinear dynamics. Fig. 2*E* illustrates the LCD prediction on a two-gene perturbation combination with neomorphic GI that was not seen during training. LCD correctly predicted most observed DE trends, both of the on-target and marker (top 20 DE) genes. *SI Appendix*, Fig. S5 provides additional plots for the other GI types.

### Recovering Causal Effects in Simulated Systems.

Next, we evaluated the CLIPR estimator component of our model. A quantitative evaluation of causal effect predictions is challenging, because the ground-truth links in gene networks are not generally known. To enable an accurate validation, we first tested CLIPR on perturbation data simulated by linear systems with single-gene shift perturbations (Eq. **3**), where the true causal structure and perturbation effects are known (*Materials and Methods*). Since CLIPR depends only on the drift $f_{q}\left(x\right)$, we evaluate the causal structure component in isolation by fitting the neural network drift directly to states x∼p(x) generated by the linear systems, omitting the measurement model (Eq. **2**) both in the ground-truth data-generating process and during inference. Our evaluation covered ground-truth systems with varying numbers of genes and perturbed genes as well as regulatory structures in A, testing ten randomly generated systems for each setting. We measured accuracy by classifying the gene–gene causal effects ${\hat{A}}_{\lambda }$ predicted by CLIPR into positive, negative, or absent and then computing the area under a (three-class) receiver operating characteristic curve (AUROC) given the ground-truth A (*Materials and Methods*). We also tested how the predicted effect strength of ${\hat{A}}_{\lambda }$ influences accuracy by computing Pearson correlations of predicted and true effects as well as F1 classification scores for the top k (@k) predicted effects by absolute value. We use LCD-CLIPR to denote the linear causal effects estimated by applying CLIPR to the learned LCD drifts.

**Fig. 3.**
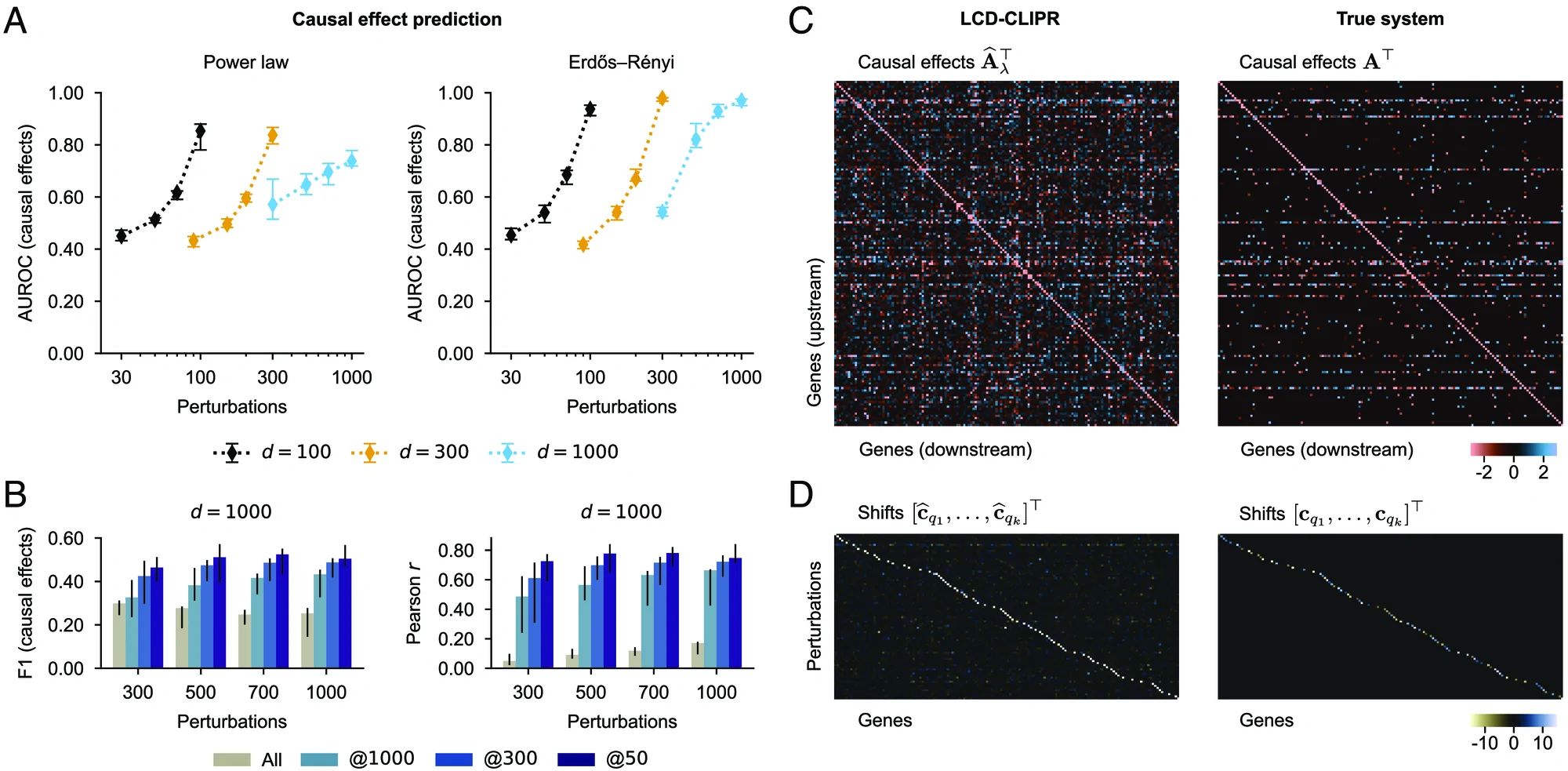
LCD-CLIPR accurately recovers causal effects in linear systems from learned diffusion drifts. (*A*) AUROC for classifying gene–gene causal effects (positive, negative, or absent) across all gene pairs. Results are shown for systems of 100, 300, and 1,000 genes across varying training perturbation counts (x-axis), comparing power-law (*Left*) and Erdős-Rényi (*Right*) regulatory dependencies in A. (*B*) F1 score for classifying gene–gene causal effects (*Left*) and average Pearson correlation between predicted and true causal effects (*Right*) for the top k predicted effects, ranked by absolute magnitude, under power-law dependencies. Markers indicate medians, error bars indicate 10 to 90th percentiles across test systems. (*C*) Comparison of causal effects inferred by LCD-CLIPR vs. the ground-truth data-generating system (300 genes). (*D*) Inferred and true perturbation vectors for the system shown in (*C*). For visual clarity, only 80 perturbations and 150 genes with the largest outgoing effects are displayed.

LCD-CLIPR achieved high accuracy in predicting both causal links and their effect signs across all sparsity structures, numbers of genes, and numbers of perturbations (Fig. 3*A*). The classification accuracy of LCD-CLIPR effects monotonically improved with the number of perturbations, reaching median AUROC values between 0.74 to 0.84 (power-law structures) and 0.93 to 0.98 (Erdős-Rényi structures) when observing d perturbations. Moreover, stronger predicted effects resulted in higher F1 scores and correlations with the ground truth (Fig. 3*B*), indicating that the strongest perturbation effects learned by the LCD drift $f_{q}$ aligned strongly with those of the true system. We later build on this finding when applying LCD-CLIPR to a genome-wide Perturb-seq screen.

Fig. 3*C* depicts the CLIPR effects extracted from a LCD drift alongside those of the true data-generating system. LCD-CLIPR correctly identified key regulator genes of the power-law regulatory structure, visible as horizontal nonzero lines with many effects. The stability-inducing negative diagonal is recovered accurately, but LCD-CLIPR tends to be less sparse, since the estimator minimizes an $\ell^{2}$ norm (*Theorem* 1). Fig. 3*D* shows the predicted and true shift vectors that generated the perturbation data. The CLIPR shift predictions ${\hat{c}}_{q}$ follow directly from the matrix $\hat{A}$ (*Corollary* 2). The estimator precisely recovered the single-gene targets, the on-target perturbation effects, and the near-zero off-target effects. The shifts ${\hat{c}}_{q}$ were not constrained to be sparse or one-hot, but recovered the perturbation pattern solely from the inferred effects ${\hat{A}}_{\lambda }$.

### Disentangling Causation from Differential Expression in Perturb-Seq.

Our results suggest that LCDs accurately predict the effects of unseen perturbation combinations and recover the regulatory structure of linear systems. We therefore reasoned that LCD-CLIPR, now including the measurement model, can be used to infer the direct causal effects among genes in Perturb-seq screens. This capability addresses a fundamental limitation of standard differential expression analyses, which capture only total causal effects accumulated through the regulatory network. This distinction matters. When gene a directly regulates gene b, perturbing a causes the differential expression of b. However, the reverse does not hold, since this differential expression may be caused by pathways of intermediate genes rather than a direct link from a to b (*SI Appendix*, Fig. S1). Understanding cellular regulatory programs therefore requires mapping direct causal mechanisms, not merely their downstream cascades.

We evaluated the capability of inferring the causal structure of direct gene–gene effects by training a LCD on a genome-wide Perturb-seq assay of the human chronic myeloid leukemia (K562) cell line (1). This screen recorded single-gene perturbations of all genes modeled by the LCD, providing significantly more perturbation responses for CLIPR to estimate regulatory structure compared to the combinations datasets used for benchmarking (Fig. 2 and *SI Appendix*, Table S1). While the perturbation dynamics learned by the LCD drift f are likely highly nonlinear, our goal is to study the degree to which the linearized CLIPR causal effects predict plausible biological structure.

**Fig. 4.**
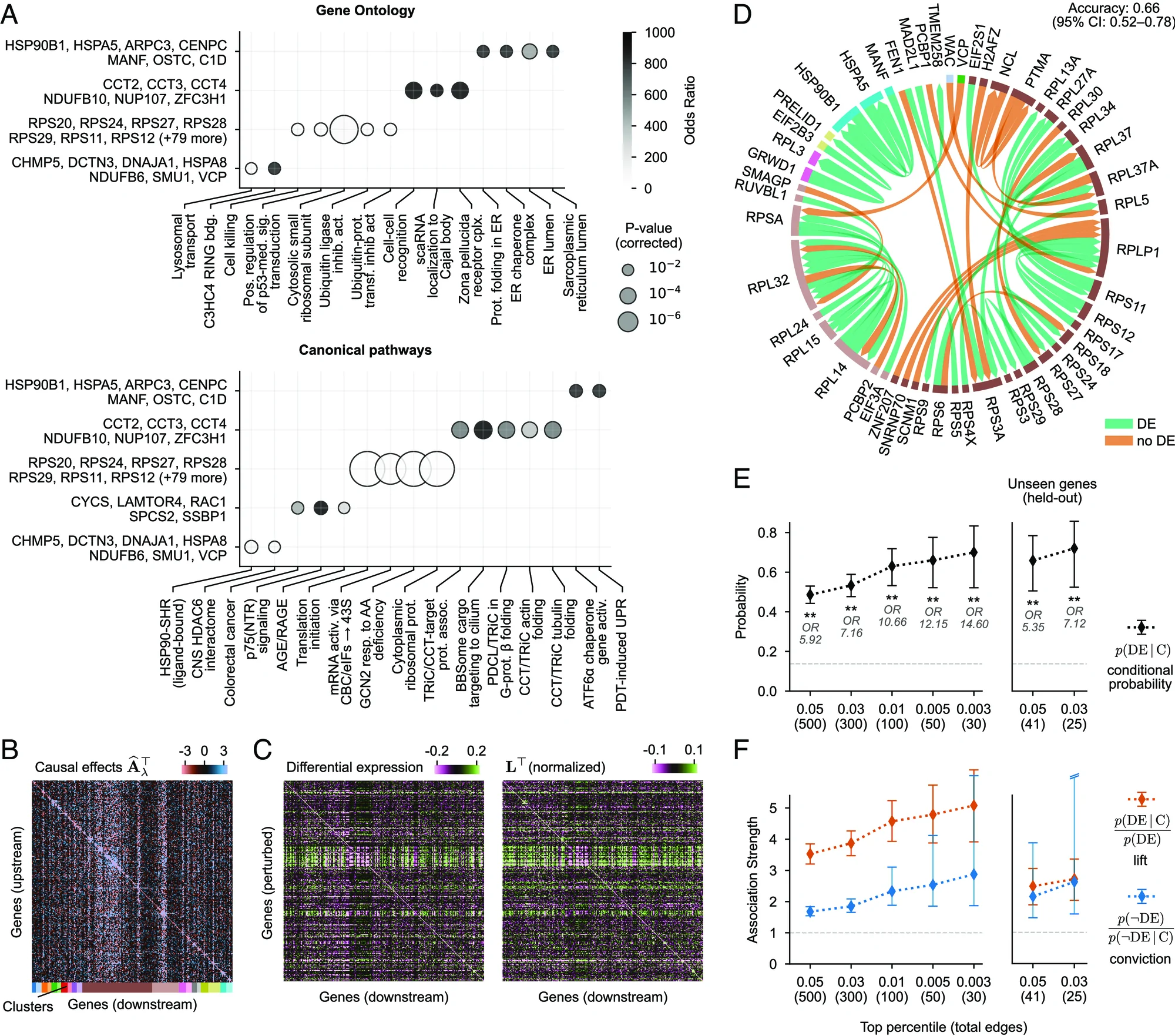
LCD-CLIPR infers direct gene–gene causal effects from genome-wide Perturb-seq data. (*A*) Hierarchical clustering based on shortest-path distances in the CLIPR graph identifies gene modules enriched for specific Gene Ontology terms (*Top*) and canonical pathways (*Bottom*). Clusters with at least one enriched term are shown. (*B*) Predicted causal effects and associated clusters from the enrichment analysis. (*C*) Comparison between observed differential expression (DE) (*Left*) and normalized $L^{⊤}$ (*Right*). For visual clarity, only the 247 genes within identified clusters and their perturbations are shown; see *SI Appendix*, Figs. S9 and S10 for full matrices. (*D*) Strongest predicted causal effects (50 total). Gene colors correspond to clusters indicated in (*B*). Edges are green where the predicted effect is validated by observed DE, and red otherwise. (*E* and *F*) Validation of inferred causal links as a function of predicted effect strength. Predicted direct causation (C) significantly increases the probability of observing DE (one-sided Fisher’s exact test, P<0.01, OR: odds ratio). Results shown for all genes (*Left*) and held-out genes not perturbed during training (*Right*). Error bars indicate 95% CIs [Wilson binomial for (*E*), bootstrap for (*F*)]. Gray dashed lines show the dataset-wide differential expression rate p(DE) (*E*) and baseline corresponding to independence (*F*).

After estimating the CLIPR matrix ${\hat{A}}_{\lambda }$ from the learned drift, we first performed hierarchical clustering of genes in the causal graph to study its network structure. We grouped genes into clusters with strong average within-cluster causal effects (*SI Appendix*). To assess whether these clusters aligned with established biological annotations, we performed gene set enrichment analysis (GSEA) (53) using known ontology terms and canonical pathways (54) (Fig. 4*A*). Several clusters showed significant enrichment for both gene ontology terms and pathways [one-sided Fisher’s exact test, P<0.01, with Benjamini–Hochberg (BH) correction for multiple testing across gene clusters and annotations (55)]. Moreover, clusters were enriched for distinct terms and pathways, suggesting that tight dependencies in the LCD-CLIPR graph organize genes into functionally coherent modules. (Fig. 4*B*) shows the predicted causal effects ${\hat{A}}_{\lambda }$ of the genes in the clusters analyzed for enrichment. This matrix is qualitatively different than the genome-wide DE matrix (Fig. 4*C*, *Left*) when ordering the perturbed genes alongside the cause (upstream) genes. Instead, the LCD-CLIPR component L closely matches the differential expressions (Fig. 4*C*, *Right*), since it captures the difference in the pre- and postperturbation equilibria (Fig. 1*C*). Moreover, causal effects are, as expected, sparser than differential expressions when considering all d=1,000 genes. We provide plots of the full matrices, including V, L, and $L^{+}$, in *SI Appendix*, Figs. S9 and S10. In addition, *SI Appendix*, Fig. S12 shows that these findings are robust with respect to choice of the regularization parameter λ.

Next, we validated the directed links predicted by LCD-CLIPR. Since the ground-truth causal effects are unknown, we tested an implication that can be assessed directly from the observed data: If gene a directly regulates gene b, then perturbing a will result in DE of b. We evaluated whether this is the case for the CLIPR estimates in the Perturb-seq screen. Specifically, we studied how reliably this implication holds as a function of the effect magnitude predicted by LCD-CLIPR, motivated by the observation that accuracy was highest for stronger predicted effects in simulated systems (Fig. 3*B*). (Fig. 4*D*) shows the 50 strongest predicted causal effects, many of which involved ribosomal protein (RP) genes, and whether a perturbation of the upstream gene caused significant DE downstream (Mann–Whitney U test, P<0.05, BH corrected for multiple testing across genes). These top 50 predictions achieved an accuracy of 0.66 (95% CI: 0.52 to 0.78), which is nontrivial compared to the baseline differential expression rate (≈0.138) across perturbations of the 1,000 genes modeled here. To quantify how well the LCD-CLIPR estimates satisfy the implication across predicted effect strengths, we computed the conditional probability of DE given predicted causal dependence (C) as well as *lift* and *conviction* (56) (*SI Appendix*). Lift measures the likelihood of DE given C compared to DE unconditionally, and conviction quantifies how much rarer violations of the implication (C but not DE) are than expected if C and DE were independent. Consistent with our results on simulated systems (Fig. 3*B*), stronger effects predicted by LCD-CLIPR significantly increased the probability of observing DE upon perturbation of the cause (Fisher’s exact test, one-sided, P<0.01) (Fig. 4*E*, *Left*). For the 50 strongest predicted causal effects, observing significant DE was approximately 4.78× more likely as measured by lift (95% CI: 3.88 to 5.73), and observing no DE was 2.53× less likely as measured by conviction (95% CI: 1.85 to 4.12), relative to the baseline DE rate (Fig. 4*F*, *Left*), suggesting that strong LCD-CLIPR links are consistent with the observed downstream effects.

CLIPR estimates a full causal structure ${\hat{A}}_{\lambda }$ irrespective of whether all or only some genes were perturbed. Hence, LCD-CLIPR makes predictions about the causal effects of genes not originally perturbed in the screen Fig. 3. To test this generalization capability, we split the 41 upstream genes shown in (Fig. 4*D*) (all genes identified as causes among the top 50 strongest predictions) into five test folds and trained new LCD models, each leaving out perturbations for one fold. We then applied CLIPR to each learned drift, reaggregated the predicted causal effects for all held-out genes, and computed the previous implication metrics using the true held-out perturbation data. Without observing any on-target perturbations, the strongest LCD-CLIPR effects predicted for the held-out genes still significantly increased the probability of observing DE when perturbing the cause gene, similar to when on-target perturbations were observed (Fig. 4*E*, *Right*). Significant DE was approximately 2.71× more likely, and no DE 2.62× less likely, relative to baseline DE rates for top 0.03 percentile of predicted edges (25 total) (Fig. 4*F*, *Right*).

Overall, our findings suggest that LCD-CLIPR can learn biologically plausible causal structure among genes, even when genes were not perturbed in the original screen. Despite only approximating the perturbation behavior of the LCD drift, CLIPR organized genes into coherent functional clusters with tight causal effects and made meaningful predictions about which genes are affected by perturbations. This highlights two main distinguishing factors between CLIPR and DE analysis: CLIPR infers direct causal effects, DE only causal descendants; and CLIPR can generalize to genes not perturbed in the original Perturb-seq assay.

## Discussion

Latent causal diffusions were designed to address the need for perturbation models that are both predictive and interpretable (4, 8, 9). As fundamentally causal models, they learn explicit, stochastic regulatory dynamics from Perturb-seq count inputs, assuming a principled measurement model. Perturbations modify the hidden states of the regulatory diffusion dynamics, and CLIPR enables interpreting these perturbed dynamics by constructing a linear approximation of the gene–gene causal structure of the entire system. LCDs and CLIPR thus stand in contrast to deep learning approaches, which are often black-box, deterministic, or based on prior gene relationship annotations (17, 19–22, 40).

Multiple temporal trajectories can generate the same aggregate population snapshots, so inference of causal dependencies from single-cell data has fundamental limits (23). Our approach alleviates this by learning from multiple snapshots, each modeled as a perturbation of the same causal regulatory dynamics. Our results suggest that these learned dynamics often predict the outcomes of unseen perturbations more accurately than existing approaches (Fig. 2*B*), while recovering causal dependencies consistent with ground-truth effects of simulated systems Fig. 3 as well as known biology Fig. 4. *Theorem* 1 shows that snapshots of sufficiently diverse experimental conditions can provably identify causality, aligned with similar results showing that interventions improve identifiability (57–59).

The LCD model and CLIPR have potential limitations. In our experiments, LCDs performed best at predicting perturbation effects when the GI of a perturbation combination is unexpected, synergistic, or suppressive (Fig. 2*D*). When GI is additive or mean predictions suffice, simpler methods like SALT or PEPER (15), which are less computationally intensive, perform competitively. Similar to CPA, the LCD perturbation model relies on embeddings learned during training and thus only allows predicting the outcomes of combinations of observed perturbations (Fig. 1*B*). LCD could potentially be extended to predict unseen single perturbations, as well as combinatorial perturbations whose constituent perturbations are not directly observed during training, by replacing learned perturbation embeddings with biologically informed representations. However, as Perturb-seq now covers the full space of individual gene perturbations (1–3), modeling their exponential number of combinations remains the key computational challenge, which LCDs enable us to do. Beyond discovering GIs, combinations are central to, for example, cell reprogramming and cell state engineering (60, 61) as well as drug combination therapies (62). LCD-CLIPR predicted nontrivial causal structure even for unperturbed genes (Figs. 3 and 4*E* and *F*, *Right*), showing that LCDs can make inferences about unperturbed components of the system. Ultimately, CLIPR remains a linear approximation of the learned LCD drift and likely the true regulatory dynamics, which should be taken into account when interpreting the presence or absence of specific links. This may explain why larger LCD-CLIPR effects tend to be more aligned with differential expression (Figs. 3*B* and 4*E* and *F*).

LCDs and CLIPR have the prospect of integrating with different biological modalities and future modeling approaches. We focused on gene perturbations to emphasize the difference between causality and differential expression, but our framework directly extends to drug perturbation screens (63), since perturbation targets need not be known to learn LCDs or compute CLIPR. Multiple batches or cell types may be integrated by conditioning the regulatory mechanisms on additional learned embeddings (Fig. 1*B*), similar to foundation model approaches (17). All perturbational datasets considered in this work were collected at a single time point and were reasonably described by a stable distributional assumption. Future extensions could relax this assumption by incorporating multiple stable states (e.g., bifurcation), as explored in prior stochastic modeling frameworks (25). Such extensions may enable modeling perturbational measurements collected across multiple time points but would require careful disentanglement of dynamical state transitions and nonstationarity from technical variation such as batch effects. Moreover, our method for inferring state densities from counts could extend to chromatin accessibility, surface protein readouts, or beyond transcriptomics. Conversely, LCD drifts could be trained from gene states inferred by other denoising techniques (64). CLIPR could render the causal structure of any diffusion interpretable, provided it models perturbations, and therefore both LCDs and CLIPR may be of interest even outside of biology. More generally, future research could explore approaches for merging the state density and drift inference steps without dropping established noise model assumptions or the separation of biological and measurement noise (Fig. 1*A*). Implementing these integrations would require calibrating assumptions made by various components and careful benchmarking with existing techniques. Overall, stationary diffusions hold promise to complement Perturb-seq screens with accurate learning-based prediction and to navigate the experimental design space by improving our causal understanding of the systems we assay.

## Materials and Methods

### Inference.

We first describe how to infer the generative model from data. The LCD model distinguishes diffusion (biological) from measurement (technical) noise by modeling discrete observations p(y) as noisy samples from the stationary diffusion densities p(x), governed by the likelihood p(y∣x; π). To achieve this separation in the generative model, we perform inference in two consecutive steps (Fig. 1*A*). Suppose we observe data $Y_{q}=\left\{y^{\left(n\right)}\right\}$ for a control condition $q_{0}$ and k perturbations $q_{1},\cdots ,q_{k}$. Inference of the model then proceeds as follows:

1. Learn state densities $p_{q}\left(x\right)$ and likelihood parameters π that maximize evidence of the observed $Y_{q}$.
  **Input:** data $Y_{q}$ for each condition q
  **Output:** samples $X_{q}∼p_{q}\left(x\right)$ and π
2. Learn drift f and embeddings $e_{q}$ that jointly fit $p_{q}\left(x\right)$.
  **Input:** states $X_{q}$ for each condition q
  **Output:**f and embeddings $e_{q}$

After both inference steps, we have learned the stationary diffusion drift f, perturbation embeddings $e_{q}$, and measurement noise parameters π. These specify the full generative model and enable data generation under unseen embeddings $e_{q^{\prime }}$. The samples $X_{q}$ are only used for fitting the drift and discarded afterward. We now describe each step in more detail.

#### Inference stage 1: Measurement model.

Inference of the state density p(x) corresponds to learning the *prior* that generates the data distribution p(y), an approach called empirical Bayes (65). Our goal is not posterior inference of p(x∣y), since we do not require the state that generated a specific datum y. Our approach to this inference problem is to model the prior p(x) as an implicit distribution that is easy to sample from and differentiate (66). Specifically, we model prior samples x∼p(x; ϕ) as x=g(ϵ,ϕ), where ϵ is random noise and g a differentiable function with respect to parameters ϕ. This choice enables a flexible distributional family for p(x) to avoid biasing the drift trained on the inferred priors in the second inference step. Implicitly modeling the prior is possible, because both of our inference steps only require samples x∼p(x; ϕ) or differentiating them with respect to ϕ.

We infer the prior $p(x;\;\phi_{q})$ of a condition q by maximizing the evidence of the observed readouts $Y_{q}$. We use separate parameters $\phi_{q}$ for each condition q but share the likelihood parameters π across conditions, as π is used for test-time generation. The evidence $\log p(Y_{q};\;\phi_{q},\pi )$ is given by

[9] $$\begin{array}{c} \begin{array}{cc} \log p(Y_{q};\;\phi_{q},\pi ) & =\sum_{n}\log\int p\left(x;\;\phi_{q}\right)p\left(y^{\left(n\right)}|x;\;\pi \right)\;\text{d}x\\ & \approx \sum_{n}LSE_{m=1}^{M}\left(\log p(y^{\left(n\right)}|x^{\left(m\right)};\;\pi )\right)-\log M, \end{array} \end{array}$$

where we approximate the integral with M samples $x^{\left(m\right)}∼p\left(x;\;\phi_{q}\right)$, written with the log-sum-exp operator $LSE_{m}(z^{\left(m\right)}):=\log{\text{Σ}}_{m}\exp(z^{\left(m\right)})$, which has a numerically stable implementation. The ZIP likelihood factors as $p(y_{g}|x_{g};\pi_{g})=\pi_{g}∥(y_{g}=0)+(1-\pi_{g})\text{Pois}(y_{g};\mu_{g}(x_{g}))$, where $∥(y_{g}=0)=1$ iff $y_{g}=0$ and $\pi_{g}=\;\text{sigmoid}\left({\overset{¯}{\pi }}_{g}\right)$ with learned logit ${\bar{\pi }}_{g}\in R$ (Eq. **2**). To account for scale differences across genes, we used the rate function $\mu_{g}\left(x_{g}\right)=\eta_{g}\log\left(1+e^{x_{g}}\right)$, where $\eta_{g}$ is a fixed gene scaling computed from control samples (*Training Details*).

Optimization of the evidence is nontrivial, since the estimator (Eq. **9**) is biased and high-variance. Expanding the joint likelihood $\log p(y|x;\;\pi )=\sum_{g=1}^{d}\log p\left(y_{g}|x_{g};\;\pi_{g}\right)$ shows that the LSE term scales linearly with d. Due to the exponential, maximizing the log-sum-exp term requires learning to generate samples with high likelihood across all d dimensions jointly, which becomes exponentially less efficient with d. We tackle this by using large M and introducing a scaling $\tau^{-1}$ of logp(y∣x; π) in the loss:

[10] $$\begin{array}{c} \begin{array}{cc} \log p^{\tau }(Y_{q},\phi_{q},\pi ) & :=\sum_{n}\tau \overset{M}{\underset{m=1}{\text{LSE}}}\left(\tau^{-1}\log p(y^{\left(n\right)}|x^{\left(m\right)};\;\pi )\right). \end{array} \end{array}$$

The temperature τ interpolates between fitting the full joint distribution including higher-order dependencies (τ=1, higher variance) and fitting the marginal distributions only (high τ, lower variance). As τ→∞, the log-sum-exp operator converges to the arithmetic mean, effectively swapping logarithm and summation like a Jensen’s lower bound, and treating the d dimensions in the joint likelihood as (unconditionally) independent, thus alleviating the variance caused by the exponential. Our final training objective $L_{\text{IP}}$ for learning the implicit prior is to minimize the combined loss

[11] $$\begin{array}{c} L_{\text{IP}}\left(Y_{q},\phi_{q},\pi \right)=-\log p^{1}\left(Y_{q},\phi_{q},\pi \right)-\log p^{\tau }\left(Y_{q},\phi_{q},\pi \right) \end{array}$$

tuned over $\tau \in \left\{5,20,100\right\}$. $L_{\text{IP}}$ is fully differentiable with respect to ϕ and π by propagating gradients through the implicit samples x=g(ϵ,ϕ). In *SI Appendix*, Fig. S8, we ablate the temperature scaling in $L_{\text{IP}}$.

#### Inference stage 2: Stationary diffusion.

Inferring the drift f and perturbation embeddings $e_{q}$ requires learning a stationary diffusion model that jointly fits the densities $p_{q}\left(x\right)$ obtained in inference stage 1. Any learning approach may in principle be used for this. In our experiments on SDEs with constant σ (Eq. **1**), score matching was computationally efficient and achieved good fit and generalization. In this setting, a drift can be inferred by denoising smoothed data $\tilde{x}∼N\left(\tilde{x};\;x,\alpha^{2}I\right)$, where x∼p(x). The drift f that minimizes the squared loss $||f\left(\tilde{x}\right)+\left(\tilde{x}-x\right)/\alpha^{2}{\left|\right|}_{2}^{2}$ in expectation satisfies $f\left(\tilde{x}\right)=\nabla \log p\left(\tilde{x};\;\alpha \right)$ under weak regularity conditions, which implies the SDE has stationary density $p(\tilde{x};\;\alpha )$ for $\sigma =\sqrt{2}$ (67). The difference between $\nabla \log p(\tilde{x};\;\alpha )$ and ∇logp(x) becomes negligible as α→0. Following Song and Ermon (68), we thus infer f by learning to denoise different smoothing levels α, conditioning the drift on α. The loss for drift $f(x;\;\theta ,e_{q},\alpha )$ is then

[12] $$\begin{array}{c} \begin{array}{cc} L_{\text{D}}\left(X_{q},\theta ,e_{q},\alpha \right) & =\sum_{n}|{\left|\alpha^{\left(n\right)}f({\tilde{x}}^{\left(n\right)};\;\theta ,e_{q},\alpha^{\left(n\right)})+\epsilon^{\left(n\right)}|\right|}_{2}^{2}, \end{array} \end{array}$$

where ${\tilde{x}}^{\left(n\right)}=x^{\left(n\right)}+\alpha^{\left(n\right)}\epsilon^{\left(n\right)}$, $\epsilon^{\left(n\right)}∼N\left(0,I_{d}\right)$, and the scales $\alpha^{\left(n\right)}$ are drawn from the geometric sequence $\alpha =[0.01,\cdots ,10]\in R^{30}$. In Eq. **12**, we scaled the norm by $\alpha^{2}$ to ensure that loss terms have comparable magnitudes. Training on multiple and large smoothing levels α avoided fitting all perturbed densities $p_{q}\left(x\right)$ unconditionally like a mixture distribution of all conditions, ignoring the embeddings $e_{q}$. Avoiding this also required careful sampling based on α (*Sampling*). For CLIPR, we condition f on the smallest $\alpha_{1}=0.01$ and thus least smoothing error (*CLIPR*). We fixed the diffusion scale σ (Eq. **1**) to $\sigma =\sqrt{2}$ throughout all experiments. This is a time-scale convention, not a model parameter, as score matching ties drift and diffusion terms together when fitting p(x). Rescaling both only rescales time, and our choice selects the parameterization in which f equals the score. We shared θ across conditions q, modeling the gene regulatory mechanisms and enabling test-time generation for unseen $e_{q}$.

Score matching fits the drift $f(x;\;\theta ,e_{q})$ to a gradient field, abstracting away the fact that θ is fit to multiple densities and coupled through $e_{q}$. While this would imply that steady-state dynamics have zero curl ($\partial {\left[f_{q}\right]}_{a}/\partial x_{b}=\partial {\left[f_{q}\right]}_{b}/\partial x_{a}$), the perturbation behavior modeled by LCDs is not symmetric. Because perturbations modify the hidden state of a nonlinear drift (Fig. 1*B*), perturbing gene a may not have the same effect on gene b as perturbing gene b has on gene a. CLIPR reveals that LCD perturbations indeed exhibit asymmetry (Figs. 3*C* and 4*B*). To learn stationary densities with nonzero curl and enable modeling, for example, negative feedback loops of three repressors (23, 36), future work could leverage more general learning objectives not based on the gradient field assumption (31), which would also allow learning state-dependent diffusions σ(x), where σ carries biological meaning rather than serving as a time-scale convention.

### Sampling.

To generate data $y∼p_{q}\left(y\right)$ for a test perturbation $e_{q}$, we conditioned f on the test embedding $e_{q}$ and simulated the diffusion to obtain samples $x∼p_{q}\left(x\right)$ from the stationary density. For a perturbation combination of $q_{a}$ and $q_{b}$, the embedding is $e_{q_{a}}+e_{q_{b}}$ (*Neural Network Architectures*). We generated diffusion samples by annealing the Euler–Maruyama method according to the trained smoothing levels α (68). The sample path approximation at step l is (69)

[13] $$\begin{array}{c} \begin{array}{c} x^{\left(l+1\right)}=x^{\left(l\right)}+f(x^{\left(l\right)};\;\theta ,e_{q},\alpha^{\left(l\right)}){\left(\alpha^{\left(l\right)}\right)}^{2}\Delta t+\xi^{\left(l\right)}\alpha^{\left(l\right)}\sqrt{2\Delta t} \end{array} \end{array}$$

for noise $\xi^{\left(l\right)}∼N\left(0,I_{d}\right)$, step size Δt, and smoothing level $\alpha^{\left(l\right)}$. In our experiments, we used $\Delta t=10^{-4}$, initialized at $x^{\left(0\right)}∼N\left(0,I_{d}\right)$, and computed 500 Euler–Maruyama steps per annealing scale in α, starting with $\alpha_{30}=10.0$ and ending at $\alpha_{1}=0.01$. The last state $x^{\left(l\right)}$ corresponds to the sample from $p_{q}\left(x\right)$ after inverting the shift and scale standardization performed on the training data (*Training Details*). For each $x∼p_{q}\left(x\right)$, we generated one observation y∼p(y∣x; π) (Eq. **2**) as the final sample from the LCD. *SI Appendix* summarizes the full LCD workflow.

### Neural Network Architectures.

For our experiments, we parameterized the drift and the implicit priors using multilayer perceptrons (MLPs). The implicit prior network g(ϵ,ϕ) transformed noise $\epsilon ∼N(\epsilon ;0,I_{64})\in R^{64}$ into samples $x=g(\epsilon ,\phi )\in R^{d}$. After an initial linear layer mapping ϵ to 64 dimensions, the network consisted of a sequence of residual MLP blocks with 64 hidden dimensions (4 blocks), followed by a linear layer mapping to d dimensions, and additional residual MLP layers with 256 hidden dimensions (2 blocks). Residual MLP blocks computed output $z^{\prime }$ from input z as $z^{\prime }:=z+\;\text{Linear}\left(\text{SiLU}\left(\text{Linear}\left(z\right)\right)\right)$, where SiLU(z):=z·sigmoid(z) elementwise and Linear(z):=Wz+b with independent parameters W,b and corresponding dimensions per layer. Residual layers stabilized training and the variance of p(x; ϕ), and the low dimensionality reduced memory, enabling larger Monte Carlo sample sizes M during training.

The drift $f(x;\;\theta ,e_{q},\alpha )$ was parameterized by a fixed α-dependent scaling to adjust for changing state and gradient magnitudes across the different smoothing scales α (70). Specifically, we modeled f as

[14] $$\begin{array}{cc} f(x;\;\theta ,e_{q},\alpha ) & =\frac{1}{\alpha }h(\frac{x}{\sqrt{1+\alpha^{2}}};\;\theta ,e_{q},\alpha ), \end{array}$$

where h is the main neural network parameterized by θ, taking state x, perturbation embedding $e_{q}$, and smoothing scale α as input. h is an MLP with two hidden layers, tanh nonlinearities, and $d_{f}\in \left\{1,024,2,048\right\}$ dimensions (Fig. 1*B*). Representations $e_{q}$ have 1,024 dimensions and are jointly learned with θ. They are added to both hidden states of the MLP before the nonlinearity following separate learned linear transformations, similar to conditioning techniques in vision models (71). We model combinations of perturbations $q_{a}$ and $q_{b}$ by adding embeddings as $e_{q_{a},q_{b}}=e_{q_{a}}+e_{q_{b}}$ and represent the control condition $q_{0}$ by the zero vector $e_{q_{0}}=0$. Last, h models the effect of α by encoding the index of α in the sequence α as a sinusoidal position embedding (72), then mapping to $d_{f}$ dimensions with a linear layer, tanh nonlinearity, and a second linear layer, and finally adding the α representation to the first hidden state of h. *SI Appendix*, Fig. S8 provides ablations of these modeling choices.

### Training Details.

As described in *Inference*, we observe data $Y_{q}$ for a control condition $q_{0}$ and k perturbations $q_{1},\cdots ,q_{k}$. Training then proceeds in two consecutive steps. We used the same training hyperparameters devised during development of the method across all experiments unless we note they are tuned otherwise (*Baselines and Hyperparameters*).

We learned the priors by minimizing $L_{\text{IP}}$ (Eq. **11**) with gradient descent for 100,000 steps (Wessels et al., k≈100) or 250,000 steps (Norman et al. and Replogle et al., k>200). We used the Adam optimizer (73) ($\beta_{1}=0$, $\beta_{2}=0.99$) with learning rate 0.0003, warmed up for 5,000 steps and cosine-decayed to zero. Each step randomly sampled a perturbation q and then updated parameters $\phi_{q}$ and π based on $Y_{q}$. The loss $L_{\text{IP}}$ was computed using a batch size of 64 and M=20,000 Monte Carlo samples. Gradients were clipped at $\ell_{2}$-norm 1.0. We used the link function $\mu_{g}\left(x_{g}\right)=\eta_{g}\log\left(1+\exp\left(x_{g}\right)\right)$ to map states $x_{g}$ to Poisson rates, where $\eta_{g}$ are gene-specific scalings (Eq. **2**). We fixed these scalings to $\eta_{g}={\bar{y}}_{g}+{\bar{s}}_{g}^{2}/{\bar{y}}_{g}-1$, lower bounded by 1, where ${\bar{y}}_{g}$ and ${\bar{s}}_{g}^{2}$ are sample means and variances of $y_{g}$ in the control data $Y_{q_{0}}$. This is the method-of-moments estimator for the Poisson mean under a ZIP model of $y_{g}$ (74) and thus calibrated $\mu_{g}\left(x_{g}\right)$ to the expected scale of $y_{g}$ (see *SI Appendix*, Fig. S8 for an ablation of this scaling). After optimization, we generated N=10,000 state samples $X_{q}={\left\{x^{\left(n\right)}\right\}}_{n=1}^{N}∼p\left(x;\;\phi_{q}\right)$ for training the diffusion.

We trained the drift f and perturbation embeddings $e_{q}$ by minimizing $L_{\text{D}}$ (Eq. **12**) with gradient descent for 50,000 steps. We used the Adam optimizer ($\beta_{1}=0.9$, $\beta_{2}=0.999$) with learning rate 0.001, cosine-decayed to zero, and weight decay tuned over $\left\{0.03,0.1,0.3,1.0,3.0\right\}$. Weight decay was most effective at controlling over- and underfitting and tuning predictive performance of LCDs. Each update step randomly selected one condition q, one smoothing level $\alpha^{\left(n\right)}$ for each $x^{\left(n\right)}$, and then updated parameters θ and $e_{q}$, using a batch size of 8,192 to compute $L_{\text{D}}$. We standardized the training data to facilitate gradient-based optimization by subtracting the control means of $X_{q_{0}}$ and dividing by the SDs across all training samples $X_{q_{0}},\cdots ,X_{q_{k}}$. *SI Appendix* summarizes the computational requirements of training.

### CLIPR.

We numerically approximated the fixed point defining the perturbation response $\ell_{f_{q}}$ by integrating the flow of f conditioned on $e_{q}$ as

[15] $$\begin{array}{cc} \ell_{f_{q}} & \approx \lim_{t\to \infty }\bar{x}\left(t\right), \end{array}$$

where $\text{d}\bar{x}\left(t\right)/\;\text{d}t=f\left(\bar{x}\left(t\right);\;\theta ,e_{q},\alpha \right)$ and $\bar{x}\left(0\right)=0$ (Fig. 1*C*). For integration, we used the same annealing scheme for α as used for sampling but dropped the diffusion term (*Sampling*). Integration starting from the control vector 0 resolves the ambiguity that $f_{q}$ may admit multiple fixed points. We assume throughout that this limit exists, which held true in all experiments. To compute CLIPR, we assembled the V and L matrices (*Theorem* 1) by conditioning the drift f separately on each perturbation embedding $e_{q}$ learned during training.

For the linear systems in Fig. 3, we used a regularization strength of λ=1/(10d) for systems of d variables, specifically $\lambda =10^{-3},3\cdot 10^{-4},10^{-4}$ for d=100,300,1,000. These values were selected by cross-validation on held-out linear systems. For real-world datasets, cross-validation is not possible, because the true causal effects are unknown. Moreover, values calibrated on linear systems are likely overly conservative under the model mismatch introduced by nonlinear perturbation responses. We therefore selected λ by plotting the norm of ${\hat{A}}_{\lambda }$ against its approximation error and identified the λ value beyond which changes to the CLIPR matrix diminish with further regularization. For our analysis in Fig. 4, this yielded $\lambda =10^{-2}$. *SI Appendix* and *SI Appendix*, Fig. S11 provide additional details.

We standardize the states p(x) before learning f, so f operates in different unit scales than the original p(x) (*Training Details*). However, we still want to interpret the causal effects of the drift in the original units when applying CLIPR to f learned in standardized units. That is, we seek the CLIPR linearization of the drift we *would have learned* had the data not been standardized. For this, we can provide the original drift $f^{x}$ as an explicit function of the data transformation and learned drift $f^{s}$ assuming the diffusion scale σ is fixed. If densities p(x) are transformed as x=m+Ts, where $m\in R^{d}$ and $T\in R^{d\times d}$ positive definite, the perturbation responses of $f^{x}\left(x\right)$ (Eqs. **5** and **6**) are given in terms of $f^{s}\left(s\right)$ as

[16] $$\begin{array}{c} \begin{array}{cc} v_{f^{x}} & =T^{-1}f^{s}\left(-T^{-1}m\right),\\ \ell_{f^{x}} & =m+T\ell_{f^{s}}. \end{array} \end{array}$$

We always applied Eq. **16** before computing CLIPR to account for data standardization before training. *SI Appendix* provides formal statements of these results and applies them to linear SDEs as a practical illustration. *SI Appendix* also gives an overview of the full LCD-CLIPR workflow.

### Datasets and Preprocessing.

We performed experiments on Perturb-seq data by Norman et al. (5) and Wessels et al. (6) (Fig. 2) and by Replogle et al. (1) (Fig. 4). All datasets were prepared with standard preprocessing steps. Initial quality control filtered genes expressed in less than 20 cells, cells measuring less than 200 genes, and perturbations with less than 100 cells, as well as outliers based on dataset-specific cutoffs for high mitochondrial RNA counts and total cell and gene counts. For benchmarking, we split the double-gene perturbations into a validation set of 20 perturbations for hyperparameter tuning and a test set used for benchmarking, which was split further into 10 folds. For a given test fold, methods were trained on control, single-gene, and double-gene perturbations of the remaining test folds. Combining all experiments, we obtained test-time predictions for all double-gene perturbations (*SI Appendix*, Fig. S2). *SI Appendix*, Table S1 lists summary statistics for the perturbations available in each dataset.

For all datasets, we modeled 1,000 highly variable genes based on Seurat-dispersions (75) of the control and single-gene perturbation data (always in training split), normalized to median control library sizes and log1p transformed. Gene selection prioritized perturbed genes and top 20 marker genes of the perturbations. Norman et al. recorded the single-gene perturbations underlying most double-gene perturbations, which enabled us to classify gene pairs into GI categories by comparing the single and double postperturbation means (*SI Appendix*). GI metrics computed on the 1,000 selected genes correlated with those computed on the full gene set (*SI Appendix*, Fig. S3). We therefore determined thresholds for GI types based on the classifications of the original study (*SI Appendix*, Fig. S4).

We also performed experiments on perturbation data generated by linear systems (Fig. 3). Here, we observed samples x∼p(x) directly rather than first inferring p(x) from counts y∼p(y∣x; π) (Eq. **2**). For each evaluation setting, we randomly generated ten linear systems (Eq. **3**) with different parameters A,b and then sampled single-gene perturbation data from the systems’ stationary distributions using one-sparse shift vectors $c_{q}$. Specifically, we sampled $A∼\;{\text{Unif}}_{\pm }\left(1,3\right)$ and b∼Unif(−3,3), where ${\text{Unif}}_{\pm }$ denotes a uniform distribution with random signs, masked A by random Erdős-Rényi and power-law sparsity structures, and then shifted the masked A by a constant diagonal to ensure stability with a maximum eigenvalue of −0.5 (31). Erdős-Rényi structures contained independently sampled nonzero entries (10 per gene in expectation) (76), and power-law structures were randomly generated with few regulator genes and many downstream genes (Barabási-Albert preferential attachment with strength 2 and 10 links per gene, orientation flipped with probability 0.1 to create feedback loops) (77). Each perturbation $c_{q}$ targeted a unique gene g with on-target shift ${\left[c_{q}\right]}_{g}∼\;{\text{Unif}}_{\pm }\left(5,15\right)$ and zero entries otherwise. The simulated datasets contained 200 samples per perturbation.

### Metrics.

For benchmarking, each method generated 1,000 samples per test perturbation Fig. 2. We computed MMD using a squared exponential kernel $k\left(y,y^{\prime }\right)=\;\exp(-||y-y^{\prime }{\left|\right|}_{2}^{2}/2d\gamma^{2})$, averaged over 10 length scales γ∈[0.01,10] in geometric progression (20). We also calculated RMSE and Pearson correlation between the predicted and observed means. For Pearson correlation (Δ), we subtracted the perturbed centroid (average of all observed perturbation means) from both the predicted and observed means (52), rather than the control centroid (19, 21), to adjust for systematic variation. We identified the top 20 differentially expressed (marker) genes relative to control using Mann–Whitney U tests with BH correction. Metrics were computed on normalized counts (library size $10^{4}$, log1p transformed). An explicit definition of the empirical MMD is given in *SI Appendix*.

For evaluating CLIPR, we computed the accuracy of ${\hat{A}}_{\lambda }$ relative to the ground-truth A by classifying the predicted effects into positive, negative, and absent using the thresholds ±0.5Fig. 3. We reported one-vs.-one AUROC scores averaged over the three class pairs, using predicted effect strength as confidences, which is insensitive to class imbalance (78). We calculated the F1 score for the three-class setting by averaging the scores predicted for each label. All metrics excluded the diagonals of the causal effect matrix (self-links).

### Baselines and Hyperparameters.

We compared LCDs to various methods Fig. 2. All figures show the baseline performance achieved by random control and perturbed cells of all training perturbations. SALT predicted postperturbation effects of double perturbations as the sum of their constituent single perturbation effects (15). Specifically, let ${\text{Δ}}_{q}\in R^{d}$ denote the mean differential expression of the normalized and log-transformed counts after perturbation q. Then, SALT generated samples for a double perturbation $q_{a},q_{b}$ by shifting randomly sampled control data by $\Delta_{q_{a}}+\Delta_{q_{b}}$. If q was not observed as a single perturbation, SALT imputed the mean across all training perturbations. *SI Appendix* provides details on the other baselines.

We tuned loss temperature τ, hidden size $d_{f}$, and weight decay for LCDs and key hyperparameters of each baseline. For this, we selected configurations that minimized the median MMD (top 20 DE genes) evaluated on a validation split of the double-gene perturbations (*SI Appendix*, Fig. S2). *Training Details* provides details for LCD and *SI Appendix* for the baselines. For the linear systems and genome-wide Perturb-seq and experiments (Figs. 3 and 4), we trained on significantly more perturbations and thus doubled the drift training steps. Because these datasets lack double perturbations for validation, we fixed hyperparameters at intermediate values of τ=20, weight decay = 1, and $d_{f}=2,048$.

## Supplementary Material

Appendix 01 (PDF)

## Data Availability

Code and evaluation scripts are provided in the GitHub repository https://github.com/larslorch/lcd-clipr (79).

## References

[1] J. M. Replogle , Mapping information-rich genotype-phenotype landscapes with genome-scale Perturb-seq. Cell **185**, 2559–2575 (2022). 10.1016/j.cell.2022.05.013 PMC9380471 35688146

[2] R. J. Carlson, M. D. Leiken, A. Guna, N. Hacohen, P. C. Blainey, A genome-wide optical pooled screen reveals regulators of cellular antiviral responses. Proc. Natl. Acad. Sci. U.S.A. **120**, e2210623120 (2023). 10.1073/pnas.2210623120 PMC10120039 37043539

[3] A. C. Huang *et al*., X-Atlas/Orion: Genome-wide Perturb-seq datasets via a scalable Fix-Cryopreserve platform for training dose-dependent biological foundation models. bioRxiv [Preprint] (2025). 10.1101/2025.06.11.659105 (Accessed 11 June 2026).

[4] J. E. Rood, A. Hupalowska, A. Regev, Toward a foundation model of causal cell and tissue biology with a Perturbation Cell and Tissue Atlas. Cell **187**, 4520–4545 (2024). 10.1016/j.cell.2024.07.035 39178831

[5] T. M. Norman , Exploring genetic interaction manifolds constructed from rich single-cell phenotypes. Science **365**, 786–793 (2019). 10.1126/science.aax4438 PMC6746554 31395745

[6] H. H. Wessels , Efficient combinatorial targeting of RNA transcripts in single cells with Cas13 RNA Perturb-seq. Nat. Methods **20**, 86–94 (2023). 10.1038/s41592-022-01705-x PMC10030154 36550277

[7] B. Cleary, A. Regev, The necessity and power of random, under-sampled experiments in biology. arXiv [Preprint] (2020). https://arxiv.org/abs/2012.12961 (Accessed 30 September 2025).

[8] A. Tejada-Lapuerta , Causal machine learning for single-cell genomics. Nat. Genet. **57**, 797–808 (2025). 10.1038/s41588-025-02124-2 40164735

[9] C. Uhler, Building a two-way street between cell biology and machine learning. Nat. Cell Biol. **26**, 13–14 (2024). 10.1038/s41556-023-01279-6 PMC11444754 38228823

[10] J. P. Taylor-King, M. Bronstein, D. Roblin, The future of machine learning within target identification: Causality, reversibility, and druggability. Clin. Pharmacol. Ther. **115**, 655–657 (2024). 10.1002/cpt.3158 38169071

[11] D. Dimitrov, S. Schrod, M. Rohbeck, O. Stegle, Interpretation, extrapolation and perturbation of single cells. Nat. Rev. Genet. **27**, 349–370 (2026). 10.1038/s41576-025-00920-4 41478891

[12] C. Bunne , How to build the virtual cell with artificial intelligence: Priorities and opportunities. Cell **187**, 7045–7063 (2024). 10.1016/j.cell.2024.11.015 PMC12148494 39672099

[13] G. I. Gavriilidis, V. Vasileiou, A. Orfanou, N. Ishaque, F. Psomopoulos, A mini-review on perturbation modelling across single-cell omic modalities. Comput. Struct. Biotechnol. J. **23**, 1886 (2024). 10.1016/j.csbj.2024.04.058 PMC11076269 38721585

[14] C. Ahlmann-Eltze, W. Huber, S. Anders, Deep-learning-based gene perturbation effect prediction does not yet outperform simple linear baselines. Nat. Methods **22**, 1657–1661 (2025). 10.1038/s41592-025-02772-6 PMC12328236 40759747

[15] T. Gaudelet *et al*., Season combinatorial intervention predictions with Salt & Peper. arXiv [Preprint] (2024). https://arxiv.org/abs/2404.16907 (Accessed 30 September 2025).

[16] C. V. Theodoris , Transfer learning enables predictions in network biology. Nature **618**, 616–624 (2023). 10.1038/s41586-023-06139-9 PMC10949956 37258680

[17] H. Cui , scGPT: Toward building a foundation model for single-cell multi-omics using generative AI. Nat. Methods **21**, 1470–1480 (2024). 10.1038/s41592-024-02201-0 38409223

[18] Y. Rosen , Toward universal cell embeddings: Integrating single-cell RNA-seq datasets across species with SATURN. Nat. Methods **21**, 1492–1500 (2024). 10.1038/s41592-024-02191-z PMC11310084 38366243

[19] Y. Roohani, K. Huang, J. Leskovec, Predicting transcriptional outcomes of novel multigene perturbations with GEARS. Nat. Biotechnol. **42**, 927–935 (2024). 10.1038/s41587-023-01905-6 PMC11180609 37592036

[20] C. Bunne , Learning single-cell perturbation responses using neural optimal transport. Nat. Methods **20**, 1759–1768 (2023). 10.1038/s41592-023-01969-x PMC10630137 37770709

[21] M. Lotfollahi , Predicting cellular responses to complex perturbations in high-throughput screens. Mol. Syst. Biol. **19**, e11517 (2023). 10.15252/msb.202211517 PMC10258562 37154091

[22] C. He, J. Zhang, M. Dahleh, C. Uhler, MORPH predicts the single-cell outcome of genetic perturbations across conditions and data modalities. bioRxiv [Preprint] (2025). 10.1101/2025.06.27.661992 (Accessed 10 April 2026).

[23] C. Weinreb, S. Wolock, B. K. Tusi, M. Socolovsky, A. M. Klein, Fundamental limits on dynamic inference from single-cell snapshots. Proc. Natl. Acad. Sci. U.S.A. **115**, E2467–E2476 (2018). 10.1073/pnas.1714723115 PMC5878004 29463712

[24] A. E. Teschendorff, A. P. Feinberg, Statistical mechanics meets single-cell biology. Nat. Rev. Genet. **22**, 459–476 (2021). 10.1038/s41576-021-00341-z PMC10152720 33875884

[25] T. B. Kepler, T. C. Elston, Stochasticity in transcriptional regulation: Origins, consequences, and mathematical representations. Biophys. J. **81**, 3116–3136 (2001). 10.1016/S0006-3495(01)75949-8 PMC1301773 11720979

[26] A. Raj, C. S. Peskin, D. Tranchina, D. Y. Vargas, S. Tyagi, Stochastic mRNA synthesis in mammalian cells. PLoS Biol. **4**, e309 (2006). 10.1371/journal.pbio.0040309 PMC1563489 17048983

[27] N. G. Van Kampen, Stochastic Processes in Physics and Chemistry, North-Holland Personal Library (Elsevier, ed. 3, 2007).

[28] V. Shahrezaei, P. S. Swain, Analytical distributions for stochastic gene expression. Proc. Natl. Acad. Sci. U.S.A. **105**, 17256–17261 (2008). 10.1073/pnas.0803850105 PMC2582303 18988743

[29] R. Lopez, J. Regier, M. B. Cole, M. I. Jordan, N. Yosef, Deep generative modeling for single-cell transcriptomics. Nat. Methods **15**, 1053–1058 (2018). 10.1038/s41592-018-0229-2 PMC6289068 30504886

[30] P. Dibaeinia, S. Sinha, SERGIO: A single-cell expression simulator guided by gene regulatory networks. Cell Syst. **11**, 252–271 (2020). 10.1016/j.cels.2020.08.003 PMC7530147 32871105

[31] L. Lorch, A. Krause, B. Schölkopf, “Causal modeling with stationary diffusions” in *Proceedings of The 27th International Conference on Artificial Intelligence and Statistics, Proceedings of Machine Learning Research*, S. Dasgupta, S. Mandt, Y. Li, Eds. (Proceedings of Machine Learning Research (PMLR), 2024), vol. 238, pp. 1927–1935.

[32] M. B. Elowitz, A. J. Levine, E. D. Siggia, P. S. Swain, Stochastic gene expression in a single cell. Science **297**, 1183–1186 (2002). 10.1126/science.1070919 12183631

[33] E. M. Ozbudak, M. Thattai, I. Kurtser, A. D. Grossman, A. Van Oudenaarden, Regulation of noise in the expression of a single gene. Nat. Genet. **31**, 69–73 (2002). 10.1038/ng869 11967532

[34] B. Øksendal, Stochastic Differential Equations (Springer, 2003).

[35] R. Thomas, D. Thieffry, M. Kaufman, Dynamical behaviour of biological regulatory networks-I. Biological role of feedback loops and practical use of the concept of the loop-characteristic state. Bull. Math. Biol. **57**, 247–276 (1995). 10.1007/BF02460618 7703920

[36] M. B. Elowitz, S. Leibler, A synthetic oscillatory network of transcriptional regulators. Nature **403**, 335–338 (2000). 10.1038/35002125 10659856

[37] U. Alon, Network motifs: Theory and experimental approaches. Nat. Rev. Genet. **8**, 450–461 (2007). 10.1038/nrg2102 17510665

[38] G. H. T. Yeo, S. D. Saksena, D. K. Gifford, Generative modeling of single-cell time series with PRESCIENT enables prediction of cell trajectories with interventions. Nat. Commun. **12**, 3222 (2021). 10.1038/s41467-021-23518-w PMC8163769 34050150

[39] M. E. Vinyard *et al*., scDiffEq: Drift-diffusion modeling of single-cell dynamics with neural stochastic differential equations. bioRxiv (2023). 10.1101/2023.12.06.570508 (Accessed 2 December 2025).

[40] S. He *et al*., Squidiff: Predicting cellular development and responses to perturbations using a diffusion model. *Nat. Methods* 23, 65–77 (2025). 10.1038/s41592-025-02877-y PMC12872408 41184550

[41] T. Zhang , cfDiffusion: Diffusion-based efficient generation of high quality scRNA-seq data with classifier-free guidance. Brie. Bioinform. **26**, bbaf071 (2025). 10.1093/bib/bbaf071 PMC11846686 39987461

[42] A. Tanay, R. Sharan, R. Shamir, Discovering statistically significant biclusters in gene expression data. Bioinform. Oxford **18**, S136 (2002). 10.1093/bioinformatics/18.suppl_1.s136 12169541

[43] E. Segal , Module networks: Identifying regulatory modules and their condition-specific regulators from gene expression data. Nat. Genet. **34**, 166–176 (2003). 10.1038/ng1165 12740579

[44] M. Isalan , Evolvability and hierarchy in rewired bacterial gene networks. Nature **452**, 840–845 (2008). 10.1038/nature06847 PMC2666274 18421347

[45] G. Heimberg, R. Bhatnagar, H. El-Samad, M. Thomson, Low dimensionality in gene expression data enables the accurate extraction of transcriptional programs from shallow sequencing. Cell Syst. **2**, 239–250 (2016). 10.1016/j.cels.2016.04.001 PMC4856162 27135536

[46] T. R. Hughes , Functional discovery via a compendium of expression profiles. Cell **102**, 109–126 (2000). 10.1016/s0092-8674(00)00015-5 10929718

[47] D. Grün, L. Kester, A. Van Oudenaarden, Validation of noise models for single-cell transcriptomics. Nat. Methods **11**, 637–640 (2014). 10.1038/nmeth.2930 24747814

[48] M. Jacobsen, A brief account of the theory of homogeneous Gaussian diffusions in finite dimensions. Front. Pure Appl. Probab. **1**, 3 (1993).

[49] J. M. Mooij, J. Peters, D. Janzing, J. Zscheischler, B. Schölkopf, Distinguishing cause from effect using observational data: Methods and benchmarks. J. Mach. Learn. Res. **17**, 1–102 (2016).

[50] L. Li *et al*., A systematic comparison of single-cell perturbation response prediction models. bioRxiv [Preprint] (2024). 10.1101/2024.12.23.630036 (Accessed 12 December 2025). PMC13557097 42715312

[51] A. Gretton, K. M. Borgwardt, M. J. Rasch, B. Schölkopf, A. Smola, A kernel two-sample test. J. Mach. Learn. Res. **13**, 723–773 (2012).

[52] R. Viñas Torné , Systema: A framework for evaluating genetic perturbation response prediction beyond systematic variation. Nat. Biotechnol. **44**, 1050–1059 (2025). 10.1038/s41587-025-02777-8 PMC13271886 40854979

[53] E. Y. Chen , Enrichr: Interactive and collaborative HTML5 gene list enrichment analysis tool. BMC Bioinform. **14**, 128 (2013). 10.1186/1471-2105-14-128 PMC3637064 23586463

[54] A. Liberzon *et al*., Molecular signatures database (MSigDB) 3.0. *Bioinformatics* **27**, 1739–1740 (2011). 10.1093/bioinformatics/btr260 PMC3106198 21546393

[55] Y. Benjamini, Y. Hochberg, Controlling the false discovery rate: A practical and powerful approach to multiple testing. J. R. Stat. Soc. Ser. B Methodol. **57**, 289–300 (1995).

[56] S. Brin, R. Motwani, J. D. Ullman, S. Tsur, “Dynamic itemset counting and implication rules for market basket data” in *Proceedings of the 1997 ACM SIGMOD International Conference on Management of Data*, J. M. Peckman, S. Ram, M. Franklin, Eds. (Association for Computing Machinery, 1997), pp. 255–264.

[57] J. Peters, P. Bühlmann, N. Meinshausen, Causal inference by using invariant prediction: Identification and confidence intervals. J. R. Stat. Soc. Ser. B Stat. Methodol. **78**, 947–1012 (2016).

[58] K. Yang, A. Katcoff, C. Uhler, “Characterizing and learning equivalence classes of causal DAGs under interventions” in *International Conference on Machine Learning* (PMLR), J. Dy, A. Krause, Eds. (2018), pp. 5541–5550.

[59] J. Zhang , Identifiability guarantees for causal disentanglement from soft interventions. Adv. Neural Inf. Process. Syst. **36**, 50254–50292 (2023).

[60] H. Wang, Y. Yang, J. Liu, L. Qian, Direct cell reprogramming: Approaches, mechanisms and progress. Nat. Rev. Mol. Cell Biol. **22**, 410–424 (2021). 10.1038/s41580-021-00335-z PMC8161510 33619373

[61] S. M. Rafelski, J. A. Theriot, Establishing a conceptual framework for holistic cell states and state transitions. Cell **187**, 2633–2651 (2024). 10.1016/j.cell.2024.04.035 38788687

[62] R. B. Mokhtari , Combination therapy in combating cancer. Oncotarget **8**, 38022 (2017). 10.18632/oncotarget.16723 PMC5514969 28410237

[63] S. R. Srivatsan , Massively multiplex chemical transcriptomics at single-cell resolution. Science **367**, 45–51 (2020). 10.1126/science.aax6234 PMC7289078 31806696

[64] D. Van Dijk , Recovering gene interactions from single-cell data using data diffusion. Cell **174**, 716–729 (2018). 10.1016/j.cell.2018.05.061 PMC6771278 29961576

[65] H. E. Robbins, “An empirical Bayes approach to statistics” in *Proceedings of the Third Berkeley Symposium on Mathematical Statistics and Probability (1954/1955), Vol. I*, J. Neyman, Ed. (University of California Press, 1956), pp. 157–163.

[66] P. J. Diggle, R. J. Gratton, Monte Carlo methods of inference for implicit statistical models. J. R. Stat. Soc. Ser. B Stat. Methodol. **46**, 193–212 (1984).

[67] P. Vincent, A connection between score matching and denoising autoencoders. Neural Comput. **23**, 1661–1674 (2011). 10.1162/NECO_a_00142 21492012

[68] Y. Song, S. Ermon, “Generative modeling by estimating gradients of the data distribution” in *Advances in neural information processing systems*, H. Wallach et al., Eds. (Curran Associates, Inc., 2019), vol. 32.

[69] S. Särkkä, A. Solin, Applied Stochastic Differential Equations (Cambridge University Press, 2019), vol. 10.

[70] Y. Song ., “Score-based generative modeling through stochastic differential equations” in *International Conference on Learning Representations* (2021).

[71] V. Dumoulin, J. Shlens, M. Kudlur, “A learned representation for artistic style” in *International Conference on Learning Representations* (2017).

[72] A. Vaswani *et al*., “Attention is all you need” in *Advances in neural information processing systems*, I. Guyon *et al*., Eds. (Curran Associates, Inc., 2017), vol. 30.

[73] D. Kingma, J. Ba, “Adam: A method for stochastic optimization” in *International Conference on Learning Representations* (2015).

[74] S. Beckett , Zero-inflated Poisson (ZIP) distribution: Parameter estimation and applications to model data from natural calamities. Involv. J. Math. **7**, 751–767 (2014).

[75] R. Satija, J. A. Farrell, D. Gennert, A. F. Schier, A. Regev, Spatial reconstruction of single-cell gene expression data. Nat. Biotechnol. **33**, 495–502 (2015). 10.1038/nbt.3192 PMC4430369 25867923

[76] P. Erdős, A. Rényi, On random graphs. Publ. Math. **6**, 290–297 (1959).

[77] A. L. Barabási, R. Albert, Emergence of scaling in random networks. Science **286**, 509–512 (1999). 10.1126/science.286.5439.509 10521342

[78] D. J. Hand, R. J. Till, A simple generalisation of the area under the ROC curve for multiple class classification problems. Mach. Learn. **45**, 171–186 (2001).

[79] L. Lorch *et al*., “Code for: Latent causal diffusions for single-cell perturbation modeling.” GitHub. https://github.com/larslorch/lcd-clipr. Deposited 24 September 2026. 10.1073/pnas.2602630123 PMC13663777 42848419

