# Supplementary material for: Latent causal diffusions for single-cell perturbation modeling
Source: Proc Natl Acad Sci U S A. 2026 Oct 8;123(41):e2602630123. doi: 10.1073/pnas.2602630123 (PMC13663777; doi:10.1073/pnas.2602630123)
Supplement: Supplementary file 1 [file pnas.2602630123.sapp.pdf]

Supporting Information for

Latent Causal Diffusions for Single-Cell Perturbation Modeling

Lars Lorch, Jiaqi Zhang, Charlotte Bunne, Andreas Krause, Bernhard Schölkopf, and Caroline Uhler

Andreas Krause
E-mail: krausea@ethz.ch

Bernhard Schölkopf
E-mail: bs@tuebingen.mpg.de

Caroline Uhler
E-mail: cuhler@mit.edu

This PDF file includes:

Supporting text
Figs. S1 to S16
Tables S1 to S3
SI References

Supporting Information Text

CLIPR Under Change of Variables

When performing a variable transformation of the states $\mathbf{x}$ before learning a drift f, learned drifts will operate in different unit scales than the original state distribution $p(\mathbf{x})$. Our goal is to obtain CLIPR estimates for f in original units given only access to the transformation parameters and the learned f. To accomplish thisprime, the following theorem provides the original drift as an explicit function of the transformation and the learned drift, assuming the diffusion scale is fixed:

Theorem 3. *Let $f^s(\mathbf{s}) = -\nabla u(\mathbf{s})$ be the drift of an SDE $\mathrm{d}\mathbf{s}(t) = f^s(\mathbf{s}(t))\mathrm{d}t + \sigma \mathrm{d}\mathbb{W}(t)$ with $u : \mathbb{R}^d \to \mathbb{R}$ that induces stationary distribution $p(\mathbf{s})$. Suppose the variables are transformed as $\mathbf{x} = \mathbf{m} + \mathbf{T}\mathbf{s}$, where $\mathbf{m} \in \mathbb{R}^d$ and $\mathbf{T} \in \mathbb{R}^{d\times d}$ is positive definite. Then, the drift $f^x(\mathbf{x})$ of an SDE that induces the stationary distribution $p(\mathbf{x})$ with the same diffusion scale σ is*

$$f^x(\mathbf{x}) = \mathbf{T}^{-1} f^s\big(\mathbf{T}^{-1}(\mathbf{x} - \mathbf{m})\big)\,.$$

Theorem 3 derives the drift of an SDE with the same diffusion scale σ as the original SDE, because σ is typically not changed based on a priori transformations such as data standardization. Without fixing σ, the transformed drift follows directly from Itô's Lemma (1) and restrictions on f and the transform can be relaxed. The gradient field and integrability condition ensure that f induces a stationary distribution and has zero curl. Since we train the drift f with score matching, which approximately yields a gradient field, Theorem 3 can be used for analyzing LCD drifts despite the standardization used in training. The following corollary gives the perturbation responses of the drift f^x in Theorem 3 as an explicit function of f^s and the parameters of the transform:

Corollary 4. *The perturbation responses of f^x in Theorem 3 can be expressed in terms of f^s as*

$$\begin{aligned}\mathbf{v}_{f^x} &= \mathbf{T}^{-1} f^s(-\mathbf{T}^{-1}\mathbf{m})\,,\\ \boldsymbol{\ell}_{f^x} &= \mathbf{m} + \mathbf{T}\boldsymbol{\ell}_{f^s}\,.\end{aligned}$$

Proof of Theorem 1

We seek the parameter matrix $\mathbf{A}$ that induces the perturbation response vectors $\{\mathbf{v}_{f_{q_1}}, \dots, \mathbf{v}_{f_{q_k}}\}$ and $\{\boldsymbol{\ell}_{f_{q_1}}, \dots, \boldsymbol{\ell}_{f_{q_k}}\}$ under the linear model (Eq. 3) with the least squared approximation error and minimum Frobenius norm.

By definition of $\widetilde{f}_q$, it must hold that $\mathbf{v}_{f_q} = \mathbf{b} + \mathbf{c}_q$ for the linear model to induce the initial perturbation response $\mathbf{v}_{f_q}$. Moreover, since the post-perturbation mean $\boldsymbol{\mu}_q$ is the unique fixed point of the linear drift, the linear model must satisfy $\boldsymbol{\ell}_{f_q} = \boldsymbol{\mu}_q$. Substituting both conditions into the analytical form of the mean (Eq. 4), we obtain the equality $\mathbf{A}\boldsymbol{\ell}_{f_q} = -\mathbf{v}_{f_q}$. Combining the conditions for all perturbations $\{q_1, \dots, q_k\}$, we obtain the system of linear equations

$$\mathbf{A}\mathbf{L} = -\mathbf{V}\,, \tag{17}$$

where $\mathbf{V}$ and $\mathbf{L}$ are defined as in the theorem statement. If $\mathbf{A}$ satisfies the equality (Eq. 17), the linear SDE model with causal effects $\mathbf{A}$ induces exactly the perturbation responses specified by $\mathbf{V}$ and $\mathbf{L}$.

In general, this system may, however, be underdetermined (infinitely many solutions $\mathbf{A}$) or inconsistent (no solution $\mathbf{A}$). We therefore seek the least squares solution $\widehat{\mathbf{A}} = \arg\min_{\mathbf{A}} \|\mathbf{A}\mathbf{L} + \mathbf{V}\|_\mathrm{F}^2$ with minimum norm $\|\mathbf{A}\|_\mathrm{F}^2$, which always exists, is unique, and is given by the Moore-Penrose pseudoinverse (2)

$$\widehat{\mathbf{A}} = -\mathbf{V}\mathbf{L}^+\,.$$

Moreover, if $\mathbf{L} \in \mathbb{R}^{d\times k}$ has full row rank, $\widehat{\mathbf{A}}$ is the only least squares solution (3). Thus, if additionally an exact solution $\mathbf{A}$ of Eq. 17 exists, where the least squares error is minimized at zero, the matrix $\widehat{\mathbf{A}}$ equals this solution. This implies that $\widehat{\mathbf{A}} = \mathbf{A}$ if the perturbation response matrices $\mathbf{V}$ and $\mathbf{L}$ are generated by a linear drift $f = \widetilde{f}$, where an exact solution exists (the true $\mathbf{A}$), and $\mathbf{L}$ has full row rank.

A sufficient condition for $\mathbf{L}$ being full row rank is that $k \geq d$ of the perturbation shift vectors $\mathbf{c}_{q_i}$ are linearly independent. This fails only in the degenerate case where $\mathbf{b}$ is an an affine combination of the perturbation shifts, i.e., $\mathbf{b} = \sum_{i=1}^k \beta_i \mathbf{c}_{q_i}$ with $\sum_{i=1}^k \beta_i = 1$. Otherwise, the vectors $\mathbf{b} + \mathbf{c}_{q_i} = \boldsymbol{\ell}_{f_{q_i}}$ are linearly independent, so $\mathbf{L}$ has full rank.

When Eq. 17 is inconsistent, the least squares solution $\widehat{\mathbf{A}}$ may not be optimal in all applications. The estimator can be made more well-behaved by regularizing the norm of the solution itself via $\widehat{\mathbf{A}}_\lambda = \arg\min_{\mathbf{A}} \|\mathbf{A}\mathbf{L} + \mathbf{V}\|_\mathrm{F}^2 + \lambda\|\mathbf{A}\|_\mathrm{F}^2$, known as Tikhonov regularization. The analytical solution to this regularized problem is given by (4)

$$\widehat{\mathbf{A}}_\lambda = -\mathbf{V}\mathbf{L}^\top\big(\mathbf{L}\mathbf{L}^\top + \lambda\mathbf{I}\big)^{-1}\,.$$

■

Proof of Corollary 2

The linear system (Eq. 3) with CLIPR matrix $\widehat{\mathbf{A}}$ satisfies Eq. 4. By definition, $\mathbf{c}_q = 0$ for the unperturbed drift f, so $\boldsymbol{\mu} = \boldsymbol{\ell}_f = -\widehat{\mathbf{A}}^{-1}\mathbf{b}$ and thus $\mathbf{b} = -\widehat{\mathbf{A}}\boldsymbol{\ell}_f$. Following the same reasoning, the perturbation vectors $\mathbf{c}_q$ are given by $\mathbf{c}_q = -\mathbf{b} - \widehat{\mathbf{A}}\boldsymbol{\ell}_{f_q}$.

■

Proof of Theorem 3

To prove the statement, we first establish the following fact:

Lemma 5. *Let $u : \mathbb{R}^d \to \mathbb{R}$ be a function such that $\exp(-2u(\mathbf{x}))$ is integrable. For any positive definite matrix $\boldsymbol{\Gamma}$, the SDE*

$$\mathrm{d}\mathbf{x}(t) = -\boldsymbol{\Gamma}\nabla u(\mathbf{x}(t))\mathrm{d}t + \boldsymbol{\Gamma}^{1/2}\mathrm{d}\mathbb{W}(t) \tag{18}$$

has stationary distribution

$$p(\mathbf{x}) \propto \exp(-2u(\mathbf{x}))\,,$$

which is independent of the scaling matrix $\boldsymbol{\Gamma}$.

Proof The Fokker-Planck equation (5) for the SDE (Eq. 18) states that the time evolution of the density $p(\mathbf{x}, t)$ is given by

$$\frac{\partial}{\partial t}p(\mathbf{x}, t) = -\nabla \cdot J(\mathbf{x}, t)\,,$$

where the probability flow is

$$J(\mathbf{x}, t) = -\boldsymbol{\Gamma}\nabla u(\mathbf{x})\,p(\mathbf{x}, t) - \frac{1}{2}\boldsymbol{\Gamma}\nabla p(\mathbf{x}, t)\,.$$

A distribution $p(\mathbf{x})$ is stationary if and only if $\frac{\partial}{\partial t}p(\mathbf{x}, t) = 0$, which by the Fokker-Planck equation is equivalent to $\nabla \cdot J(\mathbf{x}) = 0$ for all $\mathbf{x}$. A sufficient condition for this is that the probability current itself vanishes, i.e. $J(\mathbf{x}) = \mathbf{0}$. We verify that $p(\mathbf{x}) \propto \exp(-2u(\mathbf{x}))$ satisfies this condition. For this choice of $p(\mathbf{x})$, we have

$$\nabla p(\mathbf{x}) = -2p(\mathbf{x})\nabla u(\mathbf{x})\,.$$

Substituting into the expression for $J(\mathbf{x})$ yields

$$J(\mathbf{x}) = -\boldsymbol{\Gamma}\nabla u(\mathbf{x})\,p(\mathbf{x}) - \frac{1}{2}\boldsymbol{\Gamma}\nabla p(\mathbf{x}) = -\boldsymbol{\Gamma}\nabla u(\mathbf{x})\,p(\mathbf{x}) + \boldsymbol{\Gamma}\nabla u(\mathbf{x})\,p(\mathbf{x}) = \mathbf{0}\,.$$

Thus, $p(\mathbf{x}) \propto \exp(-2u(\mathbf{x}))$ is a stationary distribution, and this form is independent of $\boldsymbol{\Gamma}$. The integrability condition ensures that the normalization constant is finite, so that $p(\mathbf{x})$ is a valid probability density.

■

We now prove the main result. Given a transformation $\mathbf{x} = \phi(\mathbf{s})$, the stochastic processes $\mathbf{x}(t)$ is obtained from $\mathbf{s}(t)$ by

$$\mathbf{x}(t) = \phi(\mathbf{s}(t)) = \mathbf{m} + \mathbf{T}\mathbf{s}(t)\,.$$

By Itô's Lemma (1) applied to the general SDE form and ϕ, the SDE for $\mathbf{x}(t)$ is

$$\begin{aligned}
\mathrm{d}\mathbf{x}(t) &= \nabla\phi(\mathbf{s}(t))\,\mathrm{d}\mathbf{s}(t) \\
&= \mathbf{T}\big(f(\mathbf{s}(t))\mathrm{d}t + \sigma\mathrm{d}\mathbb{W}(t)\big) \\
&= \mathbf{T}f(\mathbf{s}(t))\mathrm{d}t + \sigma\mathbf{T}\mathrm{d}\mathbb{W}(t) \\
&= \mathbf{T}f\big(\mathbf{T}^{-1}(\mathbf{x}(t) - \mathbf{m})\big)\mathrm{d}t + \sigma\mathbf{T}\mathrm{d}\mathbb{W}(t)
\end{aligned} \tag{19}$$

This SDE induces the transformed stationary distribution $p(\mathbf{x})$. However, the diffusion scale in Eq. 19 is $\sigma\mathbf{T}$, while we week the SDE with the same diffusion scale σ as the original SDE. Lemma 5 shows that a constant positive definite scaling $\boldsymbol{\Gamma}$ does not change the stationary distribution. Hence, we apply the scaling factor $\boldsymbol{\Gamma} = \mathbf{T}^{-2}$ to Eq. 19. This yields

$$\mathrm{d}\widetilde{\mathbf{x}}(t) = \mathbf{T}^{-1}f\big(\mathbf{T}^{-1}(\,\widetilde{\mathbf{x}}(t) - \mathbf{m}\,)\big)\mathrm{d}t + \sigma\mathrm{d}\mathbb{W}(t)\,,$$

which induces the correct transformed stationary distribution with the original diffusion scale σ.

■

Proof of Corollary 4

By Theorem 3, the drift f^x is given by $f^x(\mathbf{x}) = \mathbf{T}^{-1} f^s\big(\mathbf{T}^{-1}(\mathbf{x}-\mathbf{m})\big)$. Since $\mathbf{v}_{f^x} = f^x(\mathbf{0})$ by definition, we have

$$\mathbf{v}_{f^x} = \mathbf{T}^{-1} f^s(-\mathbf{T}^{-1}\mathbf{m})\,.$$

Moreover, the fixed point $\mathbf{x}^*$ of f^x satisfies

$$f^x(\mathbf{x}^*) = \mathbf{0} \Leftrightarrow f^s\big(\mathbf{T}^{-1}(\mathbf{x}^*-\mathbf{m})\big) = \mathbf{0}$$

Since the fixed point $\mathbf{s}^*$ of f^s satisfies $f^s(\mathbf{s}^*) = \mathbf{0}$, we have $\mathbf{s}^* = \mathbf{T}^{-1}(\mathbf{x}^*-\mathbf{m})$, and conversely, $\mathbf{x}^* = \mathbf{m} + \mathbf{T}\mathbf{s}^*$. Thus,

$$\boldsymbol{\ell}_{f^x} = \mathbf{m} + \mathbf{T}\boldsymbol{\ell}_{f^s}\,.$$

■

Example: Linear SDEs and Gaussian distributions

In this section, we illustrate Theorem 1 and Corollary 2 by applying them to linear SDEs (Eq. 3), for which the stationary distribution is known analytically. Consider an SDE system with drift $f^s(\mathbf{s})$ that induces a Gaussian stationary distribution $\mathbf{s} \sim \mathcal{N}(\boldsymbol{\mu}, \mathbf{P}^{-1})$. We apply the elementwise transformation

$$\mathbf{x} = \phi(\mathbf{s}) = \mathbf{m} + \mathbf{T}\mathbf{s}\,,$$

where $\mathbf{T}$ is positive definite. Under this transformation, $\mathbf{x}$ remains Gaussian with $\mathbf{x} \sim \mathcal{N}(\mathbf{m} + \mathbf{T}\boldsymbol{\mu}, \mathbf{T}\mathbf{P}^{-1}\mathbf{T})$. Our goal is to derive the drift and causal effects of an SDE inducing the stationary distribution $p(\mathbf{x})$ using only the drift $f^s(\mathbf{s})$ of an SDE inducing the stationary distribution $p(\mathbf{s})$ and the transformation ϕ, keeping the diffusion scale fixed.

We begin with a drift f^s inducing the stationary distribution $p(\mathbf{s})$. For a Gaussian distribution, we have $\nabla \log p(\mathbf{s}) = -\mathbf{P}(\mathbf{s}-\boldsymbol{\mu})$. An SDE with stationary distribution $p(\mathbf{s})$ is thus the Langevin diffusion with

$$\mathrm{d}\mathbf{s}(t) = -\mathbf{P}(\mathbf{s}(t) - \boldsymbol{\mu})\mathrm{d}t + \sqrt{2}\mathrm{d}\mathbb{W}(t)\,.$$

This identifies the drift $f^s(\mathbf{s})$ and the causal matrix $\mathbf{A}_s$ of the linear model (Eq. 3) as

$$f^s(\mathbf{s}) = -\mathbf{P}(\mathbf{s}-\boldsymbol{\mu}) \qquad\qquad \mathbf{A}_s = -\mathbf{P}\,.$$

We now apply our results to obtain the parameters of an SDE inducing $p(\mathbf{x})$.

Theorem 3: Since $f^s(\mathbf{s})$ is the gradient of a potential and the SDE admits a stationary distribution, Theorem 3 can be directly applied to obtain the drift $f^x(\mathbf{x})$ of an SDE inducing $p(\mathbf{x})$ as its stationary distribution with the same diffusion scale:

$$f^x(\mathbf{x}) = \mathbf{T}^{-1} f^s\big(\mathbf{T}^{-1}(\mathbf{x}-\mathbf{m})\big) = -\mathbf{T}^{-1}\mathbf{P}\mathbf{T}^{-1}(\mathbf{x}-\mathbf{m}-\mathbf{T}\boldsymbol{\mu})\,.$$

As expected, this matches the drift derived directly from the closed form of $p(\mathbf{x})$ by computing $\nabla \log p(\mathbf{x})$. The corresponding matrix $\mathbf{A}_x$ of f^x in Eq. 3 is

$$\mathbf{A}_x = -\mathbf{T}^{-1}\mathbf{P}\mathbf{T}^{-1}\,.$$

Corollary 4: Applying Corollary 4, the perturbation responses of f^x are

$$\begin{aligned}\mathbf{v}_{f^x} &= \mathbf{T}^{-1} f^s(-\mathbf{T}^{-1}\mathbf{m}) = \mathbf{T}^{-1}\mathbf{P}\mathbf{T}^{-1}(\mathbf{m}+\mathbf{T}\boldsymbol{\mu})\\ \boldsymbol{\ell}_{f^x} &= \mathbf{m} + \mathbf{T}\boldsymbol{\ell}_{f^s} = \mathbf{m} + \mathbf{T}\boldsymbol{\mu}\end{aligned}$$

As shown in Theorem 1, the causal matrix $\mathbf{A}_x$ of f^x must satisfy

$$\mathbf{A}_x\boldsymbol{\ell}_{f^x} = -\mathbf{v}_{f^x}\,.$$

Substituting the expressions for $\mathbf{v}_{f^x}$ and $\boldsymbol{\ell}_{f^x}$, we obtain the condition

$$\mathbf{A}_x(\mathbf{m}+\mathbf{T}\boldsymbol{\mu}) = -\mathbf{T}^{-1}\mathbf{P}\mathbf{T}^{-1}(\mathbf{m}+\mathbf{T}\boldsymbol{\mu})\,,$$

confirming that the matrix $\mathbf{A}_x$ derived from $f^x(\mathbf{x})$ exactly satisfies the constraints specified by $\mathbf{v}_{f^x}$ and $\boldsymbol{\ell}_{f^x}$ in Corollary 4.

LCD-CLIPR Workflow

The LCD-CLIPR framework has several consecutive components, illustrated in Fig. 1 and described in Materials and Methods . Here, we give an overview of the complete workflow to highlight how the components fit together, with pseudocode specifying the inputs, outputs, learned parameters, and optimization objectives at each stage. The general workflow is as follows:

$$\text{LCD inference (Algorithm 1)} \quad \begin{matrix} \nearrow & \text{LCD sampling (Algorithm 2)} \\ \searrow & \text{CLIPR (Algorithm 3)} \end{matrix}$$

First, we infer the LCD model from data, as summarized in Algorithm 1 (SI Appendix, Fig. S14). Specifically, given single-cell count data for different perturbation conditions q, we first train the measurement model (Fig. 1A, top) by inferring the latent state densities $p_q(\mathbf{x})$ for all q, and then we train the diffusion drift f based on the inferred $p_q(\mathbf{x})$ (Fig. 1A, bottom). This yields the full LCD generative model, comprising the stationary diffusion drift f, the perturbation embeddings $\mathbf{e}_q$, and the measurement noise parameters $\boldsymbol{\pi}$.

Given the trained LCD, we can generate single-cell count samples for perturbations modeled by unseen embeddings $\mathbf{e}_{q'}$, as described in Algorithm 2 (SI Appendix, Fig. S15). We sample from the LCD by conditioning the drift f on $\mathbf{e}_{q'}$, simulating the diffusion using an annealed Euler–Maruyama scheme, and then drawing count observations given the inferred measurement noise parameters $\boldsymbol{\pi}$. In this work, we query the model with unseen combinatorial perturbations, where query embeddings $\mathbf{e}_{q'}$ are given by $\mathbf{e}_{q'} = \mathbf{e}_{q_a} + \mathbf{e}_{q_b}$ for perturbations q_a and q_b seen during training.

Given the trained LCD, we can additionally interpret the direct causal dependencies among genes by computing the CLIPR approximation of the learned drift f, as summarized in Algorithm 3 (SI Appendix, Fig. S16). We compute the perturbation responses for CLIPR by integrating the flow of f and evaluating f at the control state $\mathbf{0}$. The resulting CLIPR matrix yields the direct causal effects of the linear system that best approximates the perturbation responses of the learned drift f.

Drift, Stationary Distributions, and Causal Effects

Stationarity vs. vanishing drift A stationary distribution $p(\mathbf{x})$ of an SDE is sometimes conflated with a vanishing drift $f(\mathbf{x})$, but the two are not the same. The drift $[f(\mathbf{x})]_g$ describes how the state $\mathbf{x}$ influences the instantaneous dynamics of each gene g, while stationarity means that the distribution $p(\mathbf{x})$ induced by the SDE $\mathrm{d}\mathbf{x}(t) = f(\mathbf{x}(t))\mathrm{d}t + \sigma \mathrm{d}\mathbb{W}(t)$ (Eq. 1) does not change over time. This does not imply that regulatory interactions encoded in $[f(\mathbf{x})]_g$ are zero.

The Ornstein–Uhlenbeck SDE $\mathrm{d}\mathbf{x}(t) = -\frac{1}{2}\mathbf{x}(t)\mathrm{d}t + \mathrm{d}\mathbb{W}(t)$ illustrates this difference. This system is stable and has stationary distribution $\mathbf{x} \sim \mathcal{N}(\mathbf{0}, \mathbf{I})$, but its drift $f(\mathbf{x}) = -\frac{1}{2}\mathbf{x}$ is nonzero and depends on the current state $\mathbf{x}$. Each coordinate continuously influences its own evolution, pulling the system back toward the mean. Thus, the drift is precisely what makes the process stable and lets it converge to a stationary distribution. Conversely, setting $f(\mathbf{x}) = \mathbf{0}$ yields the Brownian motion $\mathrm{d}\mathbf{x}(t) = \mathrm{d}\mathbb{W}(t)$, which is not stable and has a time-dependent distribution $\mathbf{x}(t) \sim \mathcal{N}(\mathbf{0}, t\mathbf{I})$ whose variance grows with time.

A gene at steady state therefore need not have $[f(\mathbf{x})]_g \approx 0$. Given the continuous driving noise in the SDE, an equilibrium, and thus a stationary distribution, is only possible with a nontrivial drift. Learning $f(\mathbf{x})$ from the stationary distribution therefore recovers regulatory dynamics rather than merely transcriptional noise (Materials and Methods).

Direct causal effect vs. differential expression The difference between drift and its stationary distribution is the same as that between *direct* causal effects and their *total* downstream consequences. Our formulation models the *direct* causal effects among genes by the drift $f(\mathbf{x})$. The stationary distribution $p(\mathbf{x})$ over gene states $\mathbf{x}$ is the statistical footprint of the *accumulated* downstream effects of $f(\mathbf{x})$, obtained by unrolling these instantaneous mechanisms over time. Perturbing $f(\mathbf{x})$ cascades through the system and shifts $p(\mathbf{x})$, so a change in a marginal distribution relative to the control condition (differential expression) reflects the downstream consequences of perturbation effects that have propagated through *direct* causal effects over time.

Intuitively, the stationary distribution $p(\mathbf{x})$ gives the probability density of finding the genes in state $\mathbf{x}$, and this probability depends on the entire past history of direct causal effects that acted on $\mathbf{x}$, not only the instantaneous effect $f(\mathbf{x})$ acting on it at present. This is clearest in linear systems (Eq. 3), where the matrix $\mathbf{A}$ encodes each direct causal effect as a single scalar rather than a neural network (SI Appendix, Fig. S1). When estimating $\mathbf{A}$ with CLIPR (Theorem 1), we recover the direct causal effects that best explain the stationary distributions induced by perturbations of $f(\mathbf{x})$. The regularization parameter λ in Eq. 8 imposes a ridge penalty, so that $\widehat{\mathbf{A}}_\lambda$ captures the direct effects most strongly supported across perturbations while shrinking weakly identified ones. Fig. 3 confirms empirically that our approach lets us recover the ground-truth direct effects of linear systems even when we only observe samples from the stationary distribution.

Gene Interaction Classification

We follow the framework by Norman et al. (6) to classify gene pairs into gene interaction (GI) types. Their approach measures GIs by comparing the mean expression of double gene perturbations to those of the constituent single gene perturbations. Since the data by Wessels et al. (7) does not contain sufficient single gene perturbations to cover a substantial number of double perturbations, our GI analysis focuses on the data by Norman et al. (6) (Table S1).

Our experiments were performed on a selection of $d = 1000$ highly variable genes (Materials and Methods). Following Norman et al., we performed GI type classifications based on the $p = 570$ genes with a minimum mean unique molecular identifier (UMI) count of 0.5. We define:

- $\boldsymbol{\delta}_a \in \mathbb{R}^p$: differential expression vector for perturbation q_a
- $\boldsymbol{\delta}_b \in \mathbb{R}^p$: differential expression vector for perturbation q_b
- $\boldsymbol{\delta}_{a,b} \in \mathbb{R}^p$: differential expression vector for the combinatorial perturbation of q_a and q_b

Here, differential expression denotes the difference in mean counts between the perturbation and control (q_0) data after library standardization. Let $c_a, c_b \in \mathbb{R}$ be the regression coefficients obtained by fitting

$$[\boldsymbol{\delta}_{a,b}]_g = c_a[\boldsymbol{\delta}_a]_g + c_b[\boldsymbol{\delta}_b]_g + \epsilon_g \, ,$$

for each gene $g \in \{1, \ldots, p\}$, where ϵ_g denotes the residual error. The parameters are estimated using robust (Theil-Sen) regression to reduce sensitivity to outliers and skewness. We use the implementation by SciPy (8) with 10^5 randomly selected data point pairs and 10^3 iterations for computing the spatial median of the two coefficients.

Norman et al. define four metrics for characterizing GIs (Table S2). These metrics quantify: (i) the magnitude of c_a and c_b; (ii) the similarity among $\boldsymbol{\delta}_a$, $\boldsymbol{\delta}_b$, and $\boldsymbol{\delta}_{a,b}$, measured via distance correlation (9); (iii) the quality of the linear fit for $\boldsymbol{\delta}_{a,b}$; and (iv) the equality of contribution of $\boldsymbol{\delta}_a$ and $\boldsymbol{\delta}_b$ in explaining $\boldsymbol{\delta}_{a,b}$. Based on these metrics, Norman et al. classify GIs into six categories: suppression, additivity, synergy, epistasis, redundancy, and neomorphism.

The original GI classifications by Norman et al. (6) were annotated based on GI metrics computed over the full gene set, whereas our analysis focused on $d = 1000$ highly variable genes. Although our metrics remain strongly correlated to those computed in the original study, preprocessing choices and gene selection affect their values and thus the GI classifications (Fig. S3). We therefore re-calibrated the classification thresholds for our gene set, following an approach similar to that of Roohani et al. (10). Specifically, we determined the GI metric thresholds required to recover the original GI annotations given the metrics computed under our gene selection (for robustness considering 5–95 percentiles of the computed metrics) (Fig. S4). The resulting thresholds are provided in Table S3 and were used to classify all available double gene perturbations.

Maximum Mean Discrepancy

We benchmarked methods by comparing a predicted set of N samples $\widehat{\mathbf{Y}}_q$ for perturbation q to the N_q observed post-perturbation samples $\mathbf{Y}_q$, following library size standardization and log transformation (Materials and Methods). Our primary metric was the maximum mean discrepancy (MMD), which quantifies the discrepancy between two distributions (11). Specifically, we computed an unbiased empirical estimate of the MMD with kernel k (11):

$$\mathrm{MMD}(\widehat{\mathbf{Y}}_q, \mathbf{Y}_q) = \frac{1}{N(N-1)} \sum_{n=1}^{N} \sum_{m \neq n}^{N} k(\widehat{\mathbf{y}}^{(n)}, \widehat{\mathbf{y}}^{(m)}) + \frac{1}{N_q(N_q-1)} \sum_{n=1}^{N_q} \sum_{m \neq n}^{N_q} k(\mathbf{y}^{(n)}, \mathbf{y}^{(m)}) - \frac{2}{N N_q} \sum_{n=1}^{N} \sum_{m=1}^{N_q} k(\widehat{\mathbf{y}}^{(n)}, \mathbf{y}^{(m)}) \, . \quad [20]$$

Baselines

We evaluated LCDs against various baseline methods. For GEARS (10)* and CPA (12)†, we used the original implementations by the authors. For SALT and PEPER (13), we used our own implementations. All baselines generated samples from the post-perturbation distribution by predicting a (mean) post-perturbation shift and then shifting samples from the control (q_0) data by the predicted offset. SALT, PEPER, and GEARS are designed to predict means only, and for CPA, this was the recommended approach in the implementation. As described in Materials and Methods , SALT predicts the post-perturbation shift of a double perturbation q_a, q_b as the sum $\Delta_{q_a} + \Delta_{q_b}$ of its constituent single post-perturbation shifts $\Delta_{q_a}, \Delta_{q_b} \in \mathbb{R}^d$. (13).

PEPER extends SALT by training a neural network correction that is added to the SALT offset $\Delta_{q_a} + \Delta_{q_b}$. Specifically, the method learns two multiplayer perceptrons (MLPs) $\boldsymbol{\phi}_1$ and $\boldsymbol{\phi}_2$ that predict the correction term as $\boldsymbol{\phi}_2(\boldsymbol{\phi}_1(\Delta_{q_a}) + \boldsymbol{\phi}_1(\Delta_{q_b}))$ using the central moment discrepancy loss (14). The model was trained for 30,000 steps with batch size 256 using the Adam optimizer (15) with learning rate 0.001 and weight decay tuned over $\{0.1, 0.003, 0.0001\}$. We also tuned the number of MLP hidden layers over $\{1, 2\}$ and hidden sizes over $\{256, 1024\}$.

CPA trains an autoencoder using a Gaussian reconstruction loss and an adversarial cross entropy loss that encourages a separation of latent state and perturbation information. We tuned the latent state dimensionality over $\{64, 256\}$; encoder, decoder, and adversarial classifier hidden size over $\{1024, 2048, 4096\}$, weight decay over $\{10^{-5}, 10^{-8}\}$; dropout probability of all networks over $\{0.0, 0.2\}$; and the strength of the adversarial loss over $\{0.01, 1.0\}$. Training performed a maximum number of 1000 epochs, and all networks used 2 hidden layers. The method has additional hyperparameters, for which we selected defaults based on recommendations in the original implementation by the authors.

GEARS trains a graph neural network (GNN) over gene and perturbation relationship graphs constructed from prior knowledge databases. We optimized the GEARS model for 20 epochs and tuned the batch size over $\{32, 256\}$, the Pearson correlation threshold when constructing the co-expression graph over $\{0.4, 0.8\}$, the maximum similar genes considered in the graphs over $\{5, 10, 20\}$, and the number of GNN layers over $\{1, 2\}$. The remaining hyperparameters were kept at defaults suggested by the original implementation. Hyperparameter tuning is described in Materials and Methods .

* GEARS code repository: https://github.com/snap-stanford/GEARS
† CPA code repository: https://github.com/theislab/cpa

Predictive Accuracy Across Gene Activity Levels and Differential Expression

The benchmarking metrics in Fig. 2 are computed either on the top DE genes of each held-out perturbation or the full gene set, following prior work (10, 16). We provide two complementary analyses to verify that the performance of LCDs does not simply reflect the large fraction of genes that remain unresponsive across perturbations.

First, we stratify the metrics by gene activity level (Fig. S6). We group genes into high (top 5%), medium (top 5–20%), and low (bottom 80%) activity groups according to their dynamic regulatory response, ranked by Seurat dispersions computed across the observed single-gene perturbations during the selection of highly-variable genes (Materials and Methods). LCDs largely retain their advantage over the baselines across these groupings and, in particular, outperform other methods most substantially on the most dynamically regulated genes, where stationarity is least likely to hold and prediction is most difficult.

Second, we assess how well each method identifies which genes are differentially expressed after a perturbation (Fig. S7). We report the area under the ROC curve (AUROC) and average precision for classifying which of the $d = 1000$ modeled genes are among the top 100 DE genes of a given held-out perturbation. Methods predict DE either from the mean log-fold change or from the significance of Mann-Whitney U test statistics between the predicted and control distributions. Overall, LCDs achieve the highest classification accuracy. Because the LCD model generates a full single-cell count distribution rather than shifting control cells by a predicted mean, the significance of their DE test statistics is itself highly predictive of true DE, often more so than the mean log-fold change. In contrast, the best baselines, SALT and PEPER, only achieve strong performance when using the mean log-fold change. Their P-values are uninformative, because mean-prediction baselines shift all counts in the predicted distribution by a constant, leading to a high rate of false positives in the DE tests. On the Norman dataset, the random subset of control samples performs slightly better than chance, because stochastic fluctuations in the subset mean tend to correlate with genes that are inherently more likely to be DE. We do not observe this effect on the Wessels dataset, which contains fewer than 1000 control samples and was therefore not subsampled (Materials and Methods).

Ablations of Model and Training Components

The LCD generative model and its inference procedure use several key components. To investigate their effects on the predictive performance in Fig. 2, we provide supplementary experiments that ablate key modeling and training components of the measurement model (Fig. 1A, top) and of the stationary diffusion model (Fig. 1A, bottom).

The measurement model (Eq. 2) relies on two key components. The count distribution $p(\mathbf{y})$ is shaped by the density $p(\mathbf{x})$ and by the link functions μ_g that map x_g to the Poisson rates generating y_g. In our models, we fixed the scaling factors η_g in μ_g to gene-specific constants that calibrate $\mu_g(x_g)$ to the expected scale of y_g. This lets x_g share the same relative scale across genes g, independent of the magnitude of y_g (Materials and Methods). Inferring the generative model requires learning the densities $p(\mathbf{x})$ given the observed count data $\mathbf{Y}$ before training the diffusion. We introduced a temperature scaling of the evidence to better learn the prior $p(\mathbf{x})$ that maximizes the evidence $\log p(\mathbf{Y})$ (Materials and Methods). Fig. S8 reports the results of ablating either of these components. Specifically, we fixed $\eta_g = 1$ or dropped the loss term with $\tau > 1$ in Eq. 11, leaving all other settings as in the Wessels et al. experiments in Fig. 2. Across metrics, both components proved essential to achieving the performance in Fig. 2, with each ablations performing worse than our final model. This confirms that learning an accurate prior density $p(\mathbf{x})$ is crucial to prevent error propagation when subsequently learning the diffusion model.

The stationary diffusion model (Eq. 1) depends on a parameterization of the drift f that generalizes to unseen embeddings $\mathbf{e}_q$ and remains efficiently trainable in high dimensions (Materials and Methods). In our models, we proposed modeling f by a neural network whose shared hidden states are perturbed additively by a representation of the perturbation embedding $\mathbf{e}_q$ (Fig. 1B). We contrasted this choice with prior work (e.g., 17) that multiplies the perturbation vectors with the hidden activations rather than adding it. In addition, we ablated the input and output scaling of f with respect to the smoothing scale α in the score matching loss (Eqs. 12 and 14). We set $f = h$ and only conditioned on α, rather than additionally dividing inputs and outputs by $\sqrt{1+\alpha^2}$ and α, respectively. Fig. S8 shows the results for both ablations. As with the measurement model, we found that the perturbation modeling choice and the smoothing-specific scaling are essential for obtaining good performance. In particular, naively using the MLP h to model the drift directly prevented learning altogether (Pearson $\Delta \approx 0$).

Enrichment Analysis

We analyzed the causal structure inferred from the genome-wide Perturb-seq screen (Fig. 4A) by hierarchically clustering genes based on their causal effects. Gene distances were defined as the shortest path lengths in the graph $\mathbf{W}_\lambda + \mathbf{W}_\lambda^\top$, where $\mathbf{W}_\lambda = 1/|\widehat{\mathbf{A}}_\lambda + 10^{-10}|$ to ensure symmetry and capture indirect dependency. We then clustered genes by iteratively linking sets with minimal average distances. We obtained the final set of tight gene clusters by cutting the dendrogram at the lowest height that still grouped ribosomal protein genes into a single cluster, which constituted a large, tight cluster upon visual inspection (SI Appendix, Fig. S9). Clusters containing fewer than five genes as well as genes not assigned to any cluster after cutting the dendrogram were excluded from further analysis. We performed GSEA on the predicted gene clusters by testing for overrepresentation in gene ontology terms (C5.GO) and canonical pathways (C2.CP) of the MSigDB database (18), using one-sided Fisher's exact tests and correcting for multiple testing across both gene clusters and annotations. Fig. 4A shows top five enriched terms per predicted cluster, ranked by odds ratio.

Lift and Conviction

We validated the direct causal effects inferred by LCD-CLIPR from the genome-wide Perturb-seq screen by studying whether the predicted causal effects (C) are consistent with the observed differential expressions (DE) (Figs. 4D–F). Specifically, we evaluated whether the implication C $\Rightarrow$ DE holds across gene pairs (a, b), where

$$\begin{array}{c}\text{Direct causal effect}\\ a \to b\end{array} \text{ (C)} \quad \Longrightarrow \quad \begin{array}{c}\text{Perturbation of } a \text{ results in}\\ \text{differential expression of } b\end{array} \text{ (DE)} \; .$$

This implication holds assuming the perturbation of gene a has a statistically detectable effect on the expression of gene b and that the effect does not cancel out with indirect effects of other pathways. We computed the conditional probability (or accuracy) $p(\mathrm{DE} \,|\, \mathrm{C})$ as well as lift and conviction (19) to quantify how consistent the predictions (C) are with the implication C $\Rightarrow$ DE given the observed perturbation data (DE). Lift measures the likelihood of DE given C compared to DE unconditionally, while conviction quantifies how much less likely an implication violation (C but not DE) is given a predicted direct causal effect (C):

$$\mathrm{lift}(\mathrm{C}, \mathrm{DE}) := \frac{p(\mathrm{DE} \,|\, \mathrm{C})}{p(\mathrm{DE})} \qquad \mathrm{conviction}(\mathrm{C}, \mathrm{DE}) := \frac{p(\neg\mathrm{DE})}{p(\neg\mathrm{DE} \,|\, \mathrm{C})} \, .$$

Lift provides a more interpretable measure of whether C implies DE than classification accuracy, since the differential expression rate $p(\mathrm{DE})$ is low (≈ 0.138). It quantifies how much more likely differential expression is when LCD-CLIPR predicted a direct causal effect than otherwise. By contrast, conviction is asymmetric and thus characterizes how strongly the implication holds directionally, since $\mathrm{conviction}(\mathrm{C}, \mathrm{DE}) \neq \mathrm{conviction}(\mathrm{DE}, \mathrm{C})$. In short, lift measures the joint occurrence of C and DE relative to if they were independent, while conviction quantifies the failure rate of the implication.

We estimated the probabilities by computing the frequency of C and DE across all $d(d-1)$ gene pairs a, b, excluding on-target effects ($a = b$). We labeled a gene pair (a, b) as C if its absolute edge weight in $\widehat{\mathbf{A}}_\lambda$ was in the specified top percentile of edges (see x-axis of Figs. 4E and F). We labeled (a, b) as DE by performing Mann-Whitney U tests comparing the true perturbed and control samples ($P < 0.05$, BH corrected for multiple testing across genes, individually per perturbation). To avoid leakage in the generalization experiment, the percentile threshold defining C was computed using only edges outgoing from genes perturbed during training and then applied to the held-out perturbed genes.

Regularization Strength Selection and Sensitivity Analysis for CLIPR

The parameter λ of the regularized CLIPR estimate $\widehat{\mathbf{A}}_\lambda$ (Eq. 8) controls the norm of the estimated causal effects via

$$\widehat{\mathbf{A}}_\lambda = \arg\min_{\mathbf{A}} \|\mathbf{A}\mathbf{L} + \mathbf{V}\|_{\mathrm{F}}^2 + \lambda \|\mathbf{A}\|_{\mathrm{F}}^2$$

(see proof of Theorem 1). Regularization is essential when the perturbation responses $\mathbf{V}$ and $\mathbf{L}$ are induced by nonlinear diffusion dynamics, which can lead to systems of equations $\mathbf{A}\mathbf{L} = -\mathbf{V}$ (Eq. 17) that are inconsistent.

While λ can be tuned by cross-validation on simulated systems, this is infeasible for real-world systems, where ground-truth causal effects are unknown. For our analysis of the genome-wide Perturb-seq screen (Fig. 4), we instead select the level of regularization λ beyond which changes in $\widehat{\mathbf{A}}_\lambda$ diminish relative to the additional approximation error they incur in fitting the true perturbation responses $\mathbf{V}$ and $\mathbf{L}$. This L-curve criterion (20) is a standard heuristic for selecting Tikhonov regularization in problems with inconsistent systems of equations. Fig. S11 plots the norm of the CLIPR matrix $\widehat{\mathbf{A}}_\lambda$ against its approximation error for the system in Eq. 17. We identify the bend in this curve, corresponding to the point of maximum curvature, to select the regularization level as $\lambda = 10^{-2}$ for the analysis in Fig. 4. Empirically, this coincides with the smallest λ at which structural patterns in the CLIPR matrix $\widehat{\mathbf{A}}_\lambda$ have emerged and strong clusters no longer change visually under further regularization.

Fig. S12 examines the robustness of this choice by repeating the causal graph and gene set enrichment analysis of Fig. 4 at the λ values shown in Fig. S11, keeping the hierarchical clustering height fixed to that for $\lambda = 10^{-2}$ (Materials and Methods). The recovered gene clusters and enriched gene ontology terms show substantial agreement across the selected value $\lambda = 10^{-2}$ and the higher value $\lambda = 10^{-1}$, with all terms enriched at the higher regularization level also enriched at the selected level. Under insufficient regularization ($\lambda = 10^{-3}$), gene clustering yields no significant enrichment, consistent with the absence of visible cluster structure in Fig. S11A. Reducing the clustering cutoff for this setting to allow finer-grained clusters does recover two enriched gene groups, a large ribosomal protein (RP) cluster enriched for the cytosolic small ribosomal subunit, and a cluster including HSP90B1, HSPA5, and MANF enriched for the endoplasmic reticulum (ER) lumen. Both of these clusters and their associated terms are also present under the other λ values, confirming that the recovered clusters in the causal structure are stable across regularization levels, with λ controlling the resolution at which clusters are resolved. Together, these results suggest that while λ requires careful selection, the qualitative causal structure recovered by CLIPR is robust across a reasonable range of values around the threshold in Fig. S11.

Computational Resources

We trained LCDs on an internal cluster with 1 GPU (20GiB) and 4 CPUs (24GiB each for Wessels. et al. and Norman et al. datasets; 120GiB each for Replogle et al. dataset). Inferring the implicit prior densities was more computationally intensive ($\sim$9h; inference step 1) than training the drift ($\sim$1h; inference step 2), because of the large number of Monte Carlo samples M needed to sufficiently reduce the bias of $\mathcal{L}_{\mathrm{IP}}$ and achieve good fit of the priors.

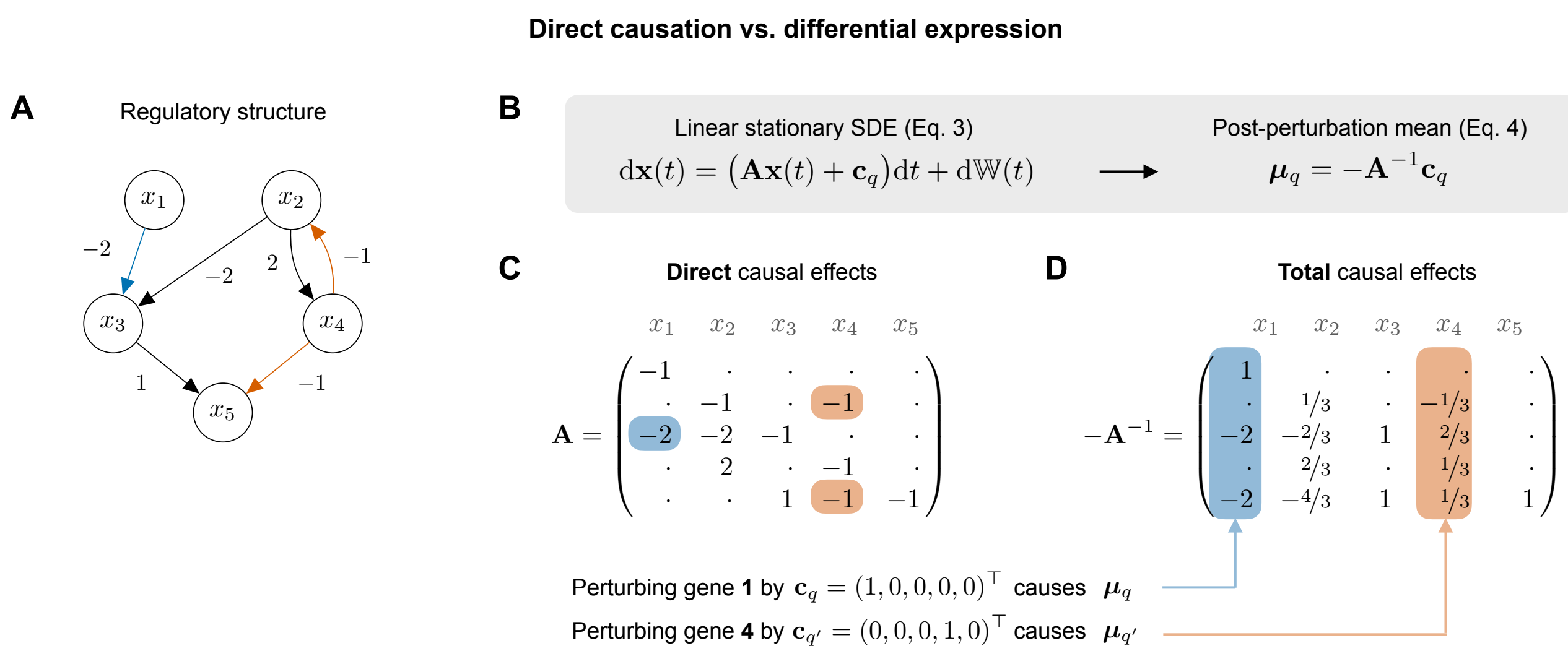

Fig. S1. Difference between direct and total causal effects, illustrated for a linear system. (**A**) Direct causal dependency structure among $d = 5$ genes, where directed edges denote direct regulatory effects in a linear drift (Eq. 3) and edge weights give their entries in $\mathbf{A}$. For simplicity, the drift has bias $\mathbf{b} = \mathbf{0}$, so the stationary distribution has observational mean $\boldsymbol{\mu} = \mathbf{0}$. Direct causal effects of x_1 and x_4 on the other gene expressions are highlighted in blue and red, respectively. (**B**) Shift perturbations $\mathbf{c}_q$ cascade through the system and shift the stationary distribution $p(\mathbf{x})$ to mean $\boldsymbol{\mu}_q = -\mathbf{A}^{-1}\mathbf{c}_q$ (Eq. 4), so a change relative to control ($\boldsymbol{\mu}_q \neq \mathbf{0}$) reflects the downstream consequences of perturbation effects that have propagated through direct causal effects over time. Perturbation data thus reveal *total* causal effects, but inferring *direct* effects requires learning the causal mechanisms $f(\mathbf{x})$. The LCD model infers a nonlinear $f(\mathbf{x})$ from noisy count measurements, and CLIPR provides the direct linear causal effects that best explain the perturbed stationary distributions without knowing $\mathbf{c}_q$ (Eq. 7). (**C**) Direct causal effect matrix $\mathbf{A}$ of the system in (**A**). Zeros are hidden for readability. (**D**) The matrix $-\mathbf{A}^{-1}$ encodes the total causal effects of shift perturbations $\mathbf{c}_q$ on the system. Unless pathways cancel out, every gene reachable from a perturbed gene by a directed path in (**A**) is affected. Because differential expression is observable in the data, we can validate whether predicted direct effects imply differential expression, which motivates our evaluation protocol in Fig. 4D–F. The converse does not hold: perturbations of x_1 or x_2, for example, induce differential expression of x_5 even though neither has a *direct* effect on x_5 (see also Materials and Methods).

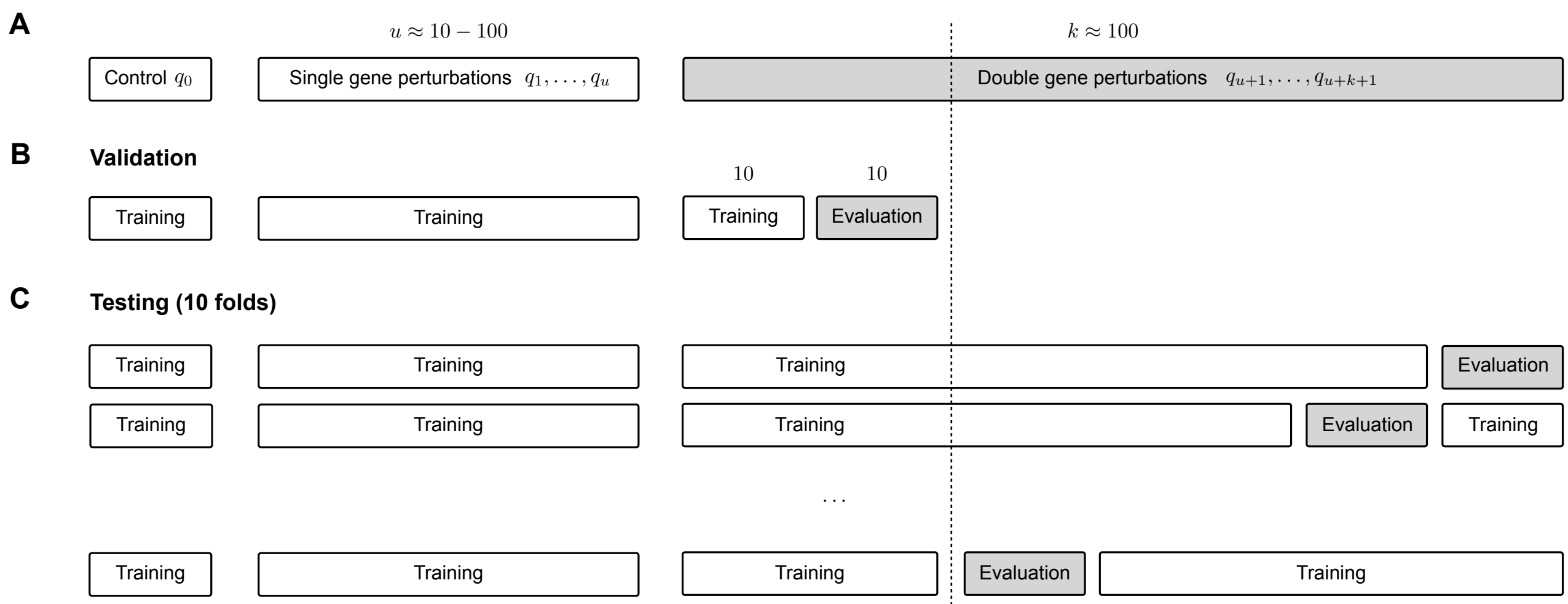

Fig. S2. Training and evaluation splits of the combinatorial gene perturbation data by Wessels et al. (7) and Norman et al. (6) used for benchmarking (Fig. 2). (**A**) Each dataset contains control samples (q_0), u single gene perturbations, and k double gene perturbations. Control and single gene perturbation samples are always in the training set. (**B**) We reserved 20 double gene perturbations for hyperparameter tuning of each method (Materials and Methods), where 10 were included in the training set and 10 were used for evaluation. (**C**) For benchmarking, we evaluated methods on 10 disjoint testing folds of the held-out double perturbations. All perturbations used for hyperparameter tuning were included in the training set to avoid information leakage. Combining all testing folds, methods generated held-out predictions for all $k - 20$ held-out double perturbations.

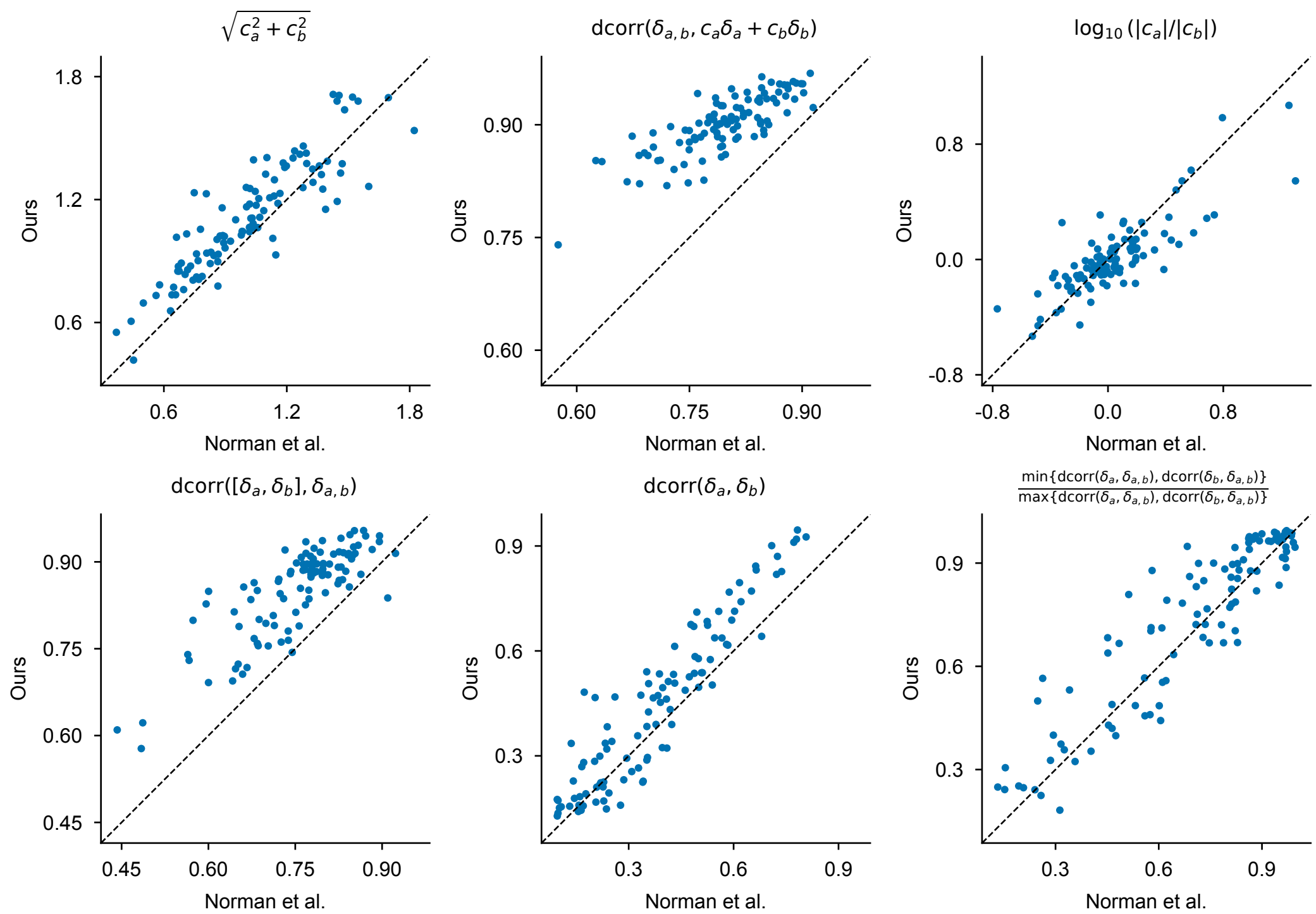

Fig. S3. Comparison of gene interaction (GI) metrics between the original study (6) and our experiments. We selected $d = 1000$ highly variable genes and applied specific preprocessing steps for quality control, which affect the computed GI metrics. Our results show strong correlation with those from the original study, motivating our decision to calibrate GI type thresholds based on the original annotations (SI Appendix, Fig. S4). Each point represents a gene pair for which a double perturbation and both constituent single perturbations are available, thus enabling GI metric computation.

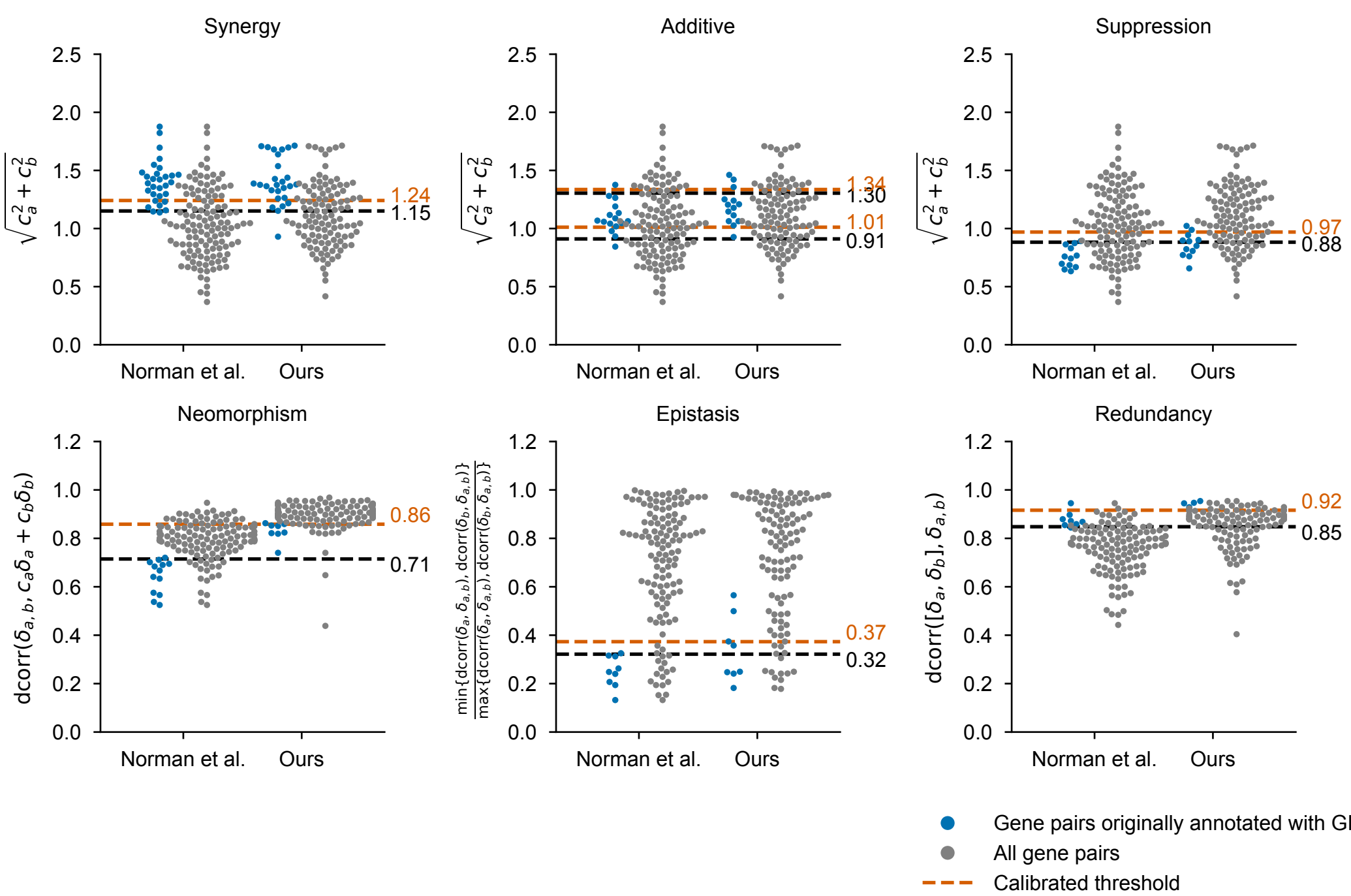

Fig. S4. Calibration of gene interaction (GI) classification thresholds between the original study (6) and our experiments. Each panel corresponds to one of six GI categories, comparing the distribution of GI metrics from the original study (left) with those from our experiments (right). Blue points indicate gene pairs annotated with the respective GI type in the original study; grey points represent all available gene pairs. Black horizontal lines denote classification thresholds derived from the original annotations; red lines show thresholds calibrated to our metrics based on the scores of the originally annotated pairs. Thresholds are summarized in SI Appendix, Table S3.

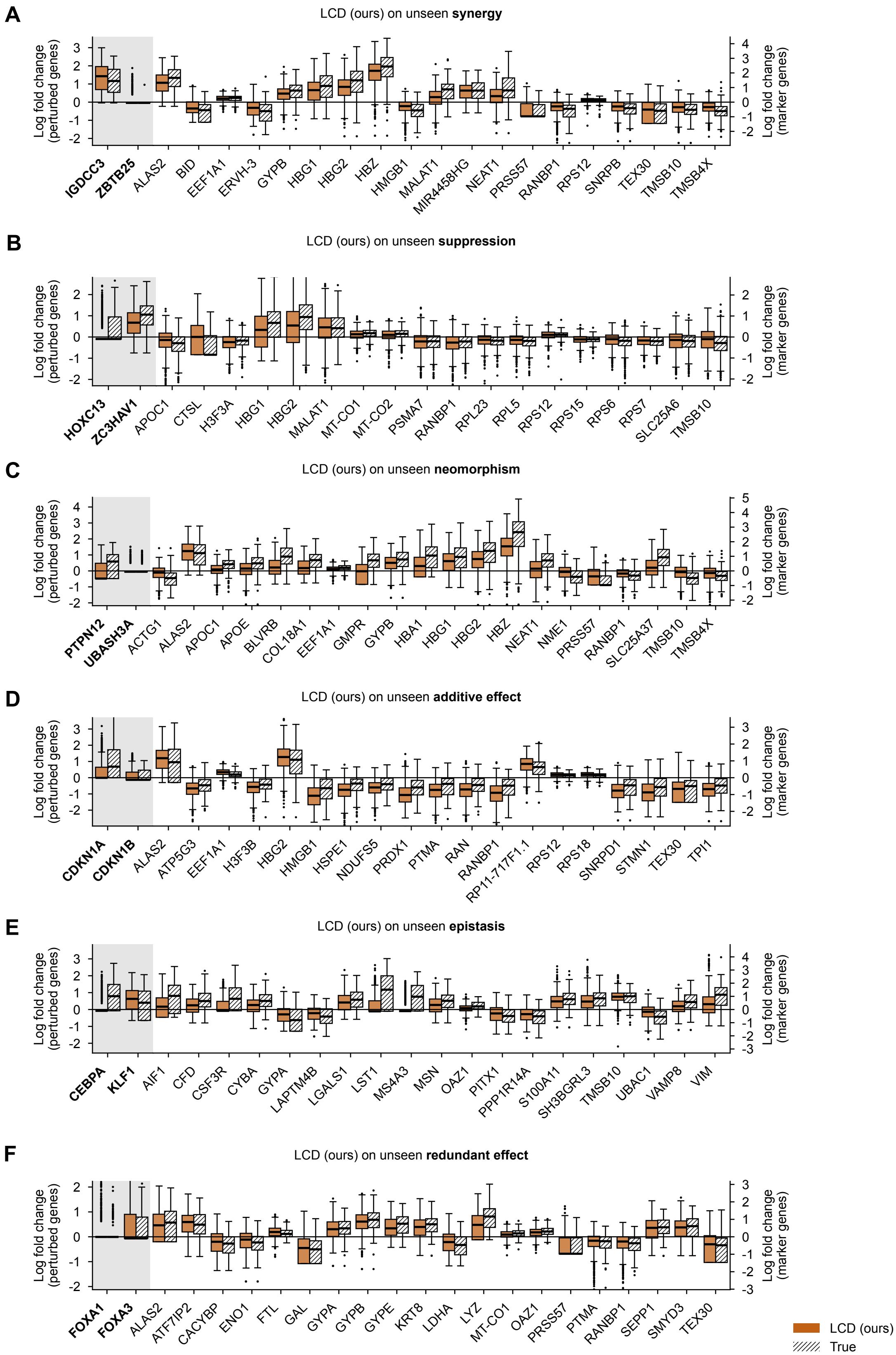

Fig. S5. LCD predictions on held-out two-gene perturbation combinations with different genetic interaction (GI) types. Boxplots show predicted vs. observed expression for the two perturbed and the top 20 DE genes of the held-out perturbation. Grey section shows the perturbed genes (bold labels). All box plots show median and interquartile range (IQR). Whiskers extend to farthest points within $1.5\times$ IQR.

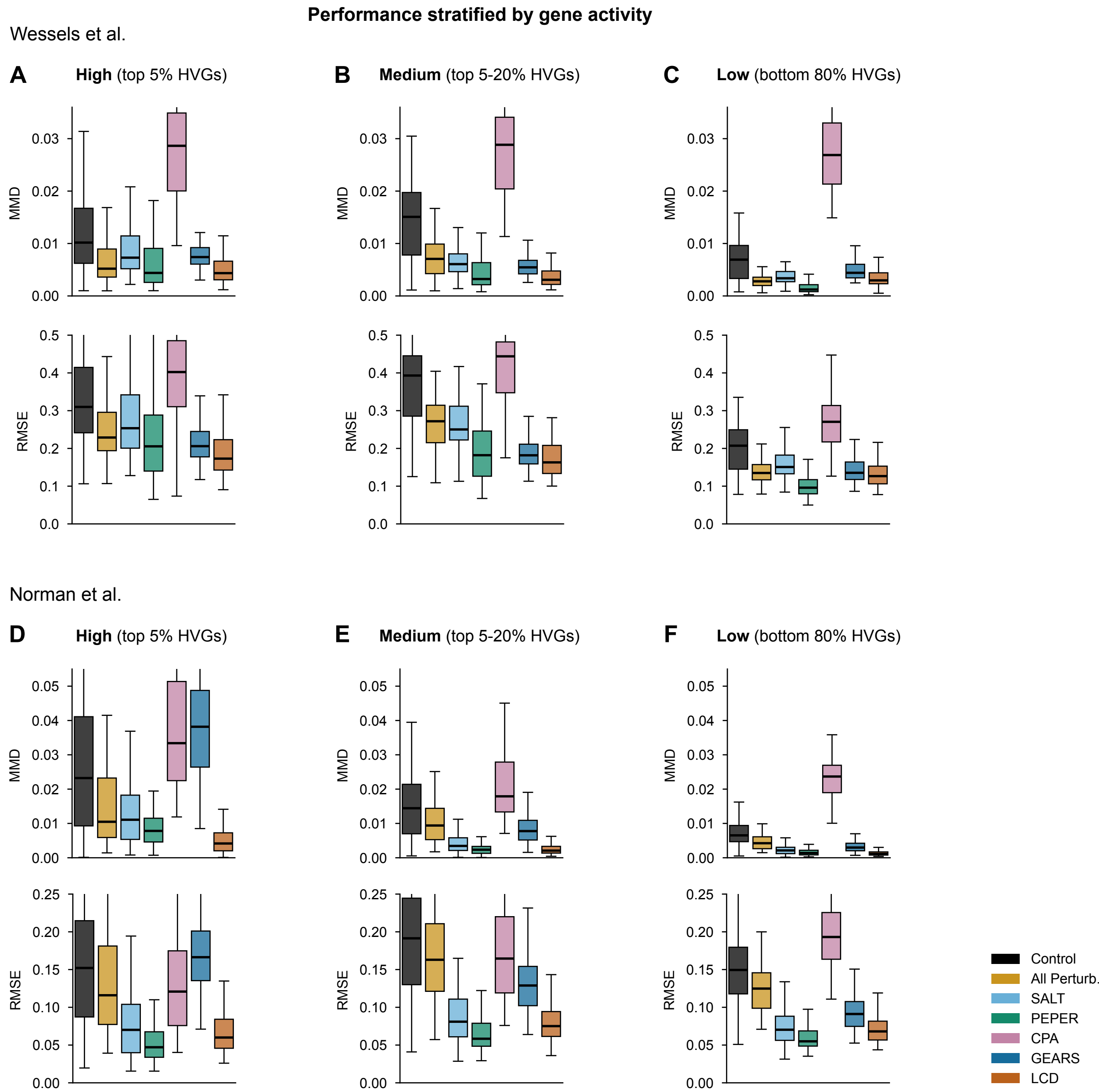

Fig. S6. LCDs accurately predict combinatorial perturbation effects for highly responsive, dynamically regulated genes. MMD and RMSE for the results in Fig. 2A-C, stratified by gene activity level. Genes are grouped by dynamic regulatory response into high (top 5%), medium (top 5–20%), and low (bottom 80%) tiers, ranked by Seurat dispersions (variability) across the observed single-gene perturbations as computed during the initial selection of highly-variable genes (HVGs) (Materials and Methods). Top (**A-C**) and bottom (**D-F**) panels show metrics for the Wessels et al. and Norman et al. data, respectively. Prediction error is generally largest for the most dynamically regulated genes (**A and D**). LCDs outperform the baselines most substantially on the highly responsive (top 5%) gene set, particularly relative to state-of-the-art methods GEARS and CPA. Metric axes use a linear scale to highlight differences error magnitudes across gene groups. Box plots show the median and interquartile range (IQR). Whiskers extend to farthest points within $1.5\times$ IQR.

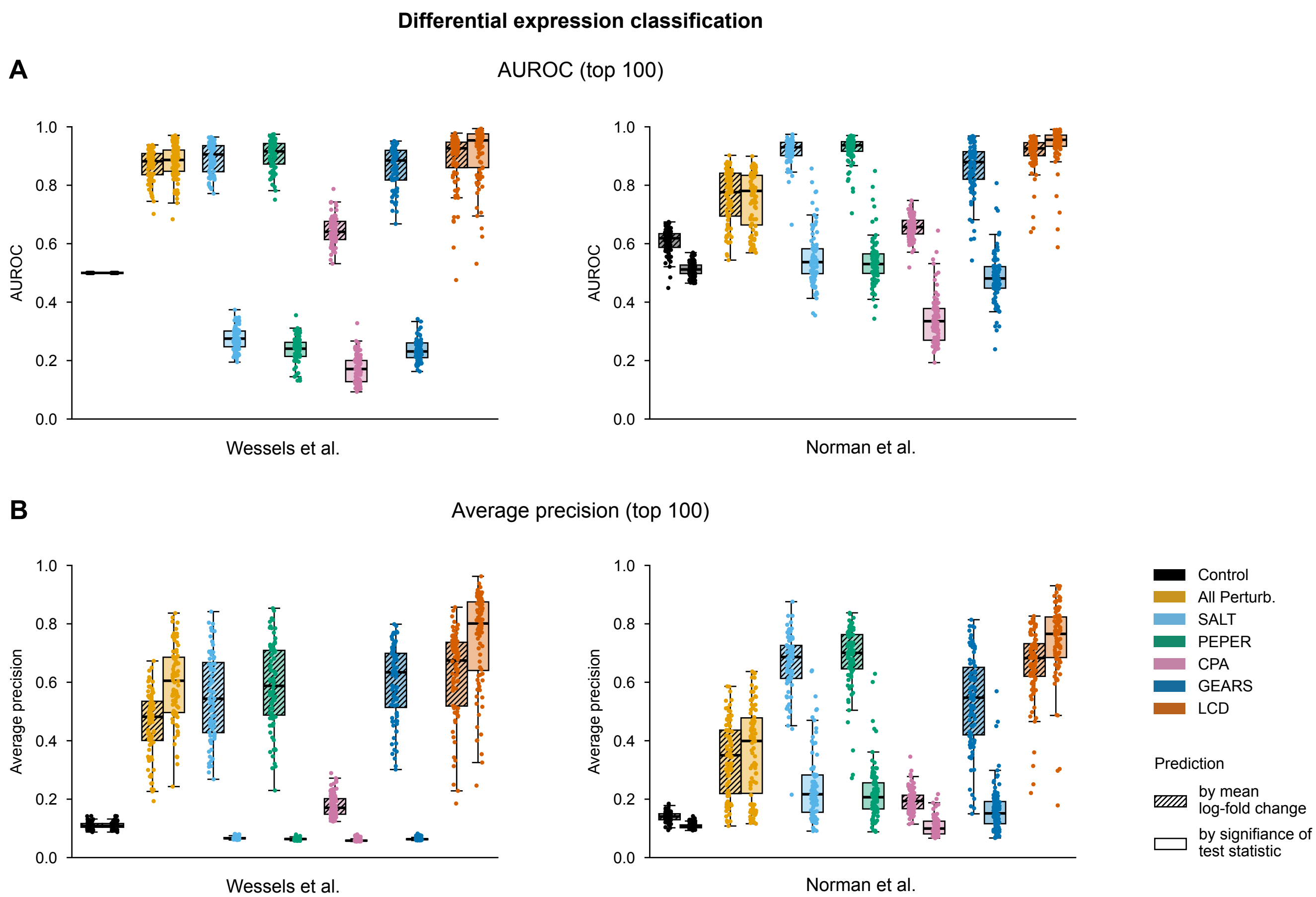

Fig. S7. LCDs outperform baselines at classifying genes as differentially expressed (DE), both from mean log-fold change and DE tests on the predicted distribution. AUROC (**A**) and average precision (**B**) for classifying which of the $d = 1000$ modeled genes are among the top 100 DE genes of a given held-out combinatorial perturbation, ranked by significance of the test statistic. Metrics are computed from the predictions in Fig. 2A–C for Wessels et al. (left panels) and (**B**) Norman et al. (right panels). True DE genes are determined by two-sided Mann-Whitney U tests ($P < 0.05$, BH corrected per perturbation). DE is predicted either from the mean log-fold change (hatched) or from the significance of Mann-Whitney U test statistics between the predicted and control distribution (not hatched), computed as for the true DE genes. SALT, PEPER, CPA, and GEARS only predict mean changes and then shift control cells (SI Appendix). Because this shifts all counts in the predicted distribution equally, their DE test statistics become uninformative (yielding false positives), and only the mean log-fold change is predictive of DE. By contrast, the LCD generative model samples valid single-cell counts from the post-perturbation distributions. As a result, the significance of DE test statistics is highly predictive of true DE, often more so than the mean log-fold change. Box plots show the median and IQR across perturbations, with whiskers extending to the farthest points within $1.5\times$ IQR. Each overlaid dot represents a single perturbation.

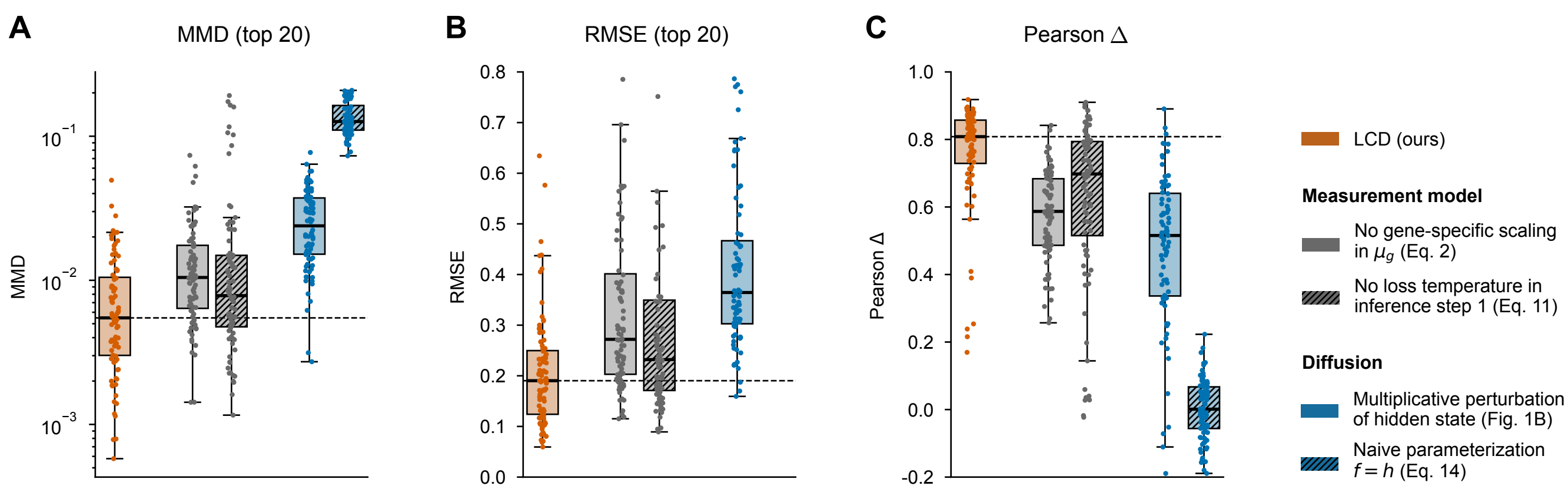

Fig. S8. Ablations of LCD modeling and inference components. Performance for predicting combinatorial perturbation effects (Fig. 2) in the dataset of Wessels et al. when ablating important choices in the measurement and diffusion modeling components. Grey LCD variants drop components of the measurement model: the gene-specific scaling (Eq. 2) and the loss temperature for learning $p(\mathbf{x})$ (Eq. 11). Blue LCD variants alter the diffusion model: a naive drift parameterization (setting $f = h$ rather than dividing inputs and outputs by $\sqrt{1+\alpha^2}$ and α, respectively) (21) and a drift in which the perturbation effect is multiplied rather than added to the hidden state of f (cf., Fig. 1B) (17). MMD and RMSE are computed on the top 20 DE genes, and Pearson Δ on the full mean expression vectors, as in Fig. 2. Base model performance from Fig. 2 is shown in red, with the median indicated by a horizontal dashed line. All box plots show the median and interquartile range (IQR). Whiskers extend to farthest points within $1.5\times$ IQR, and each dot represents one perturbation.

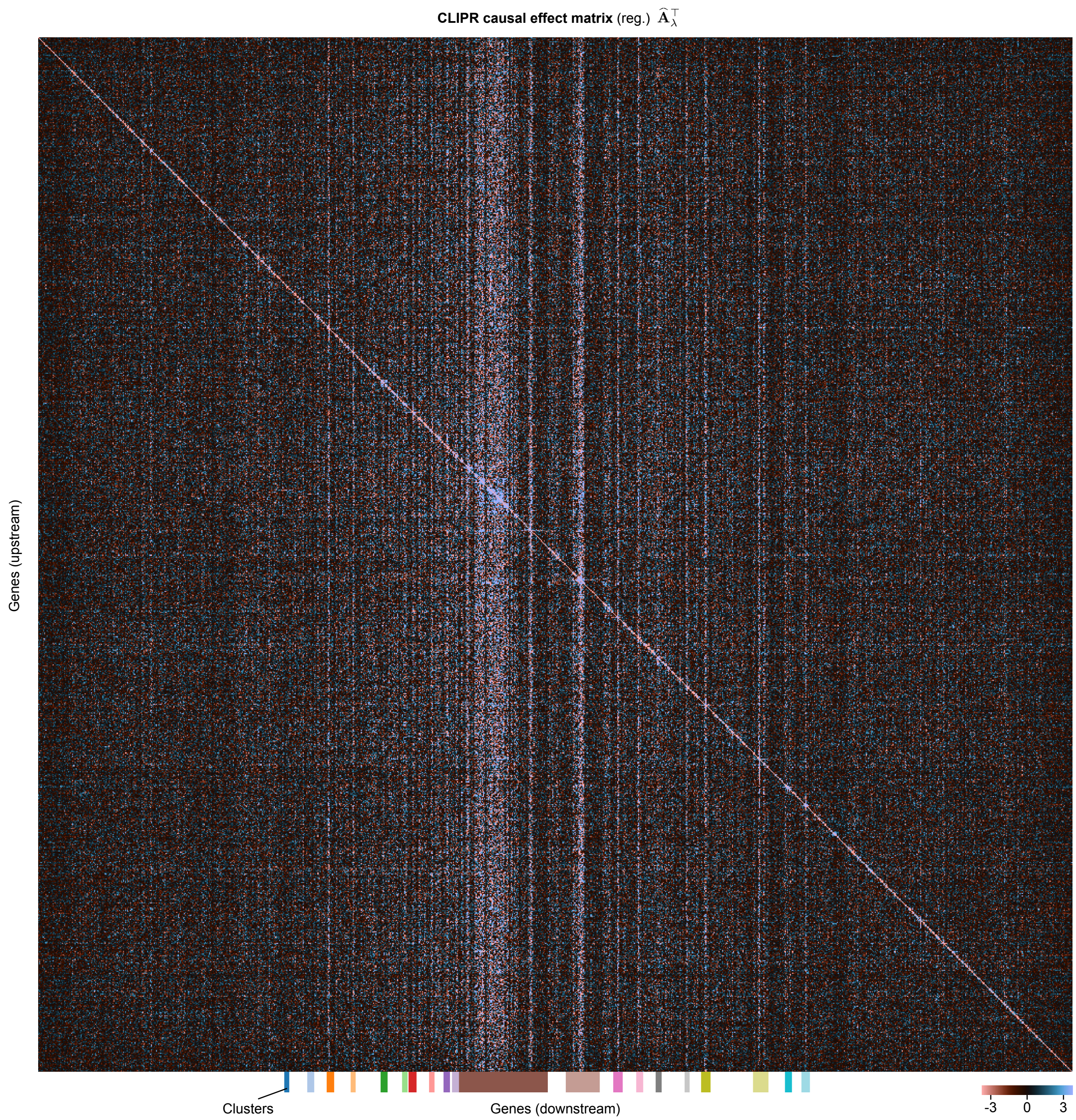

Fig. S9. LCD-CLIPR estimate of the direct gene-gene causal effects among all $d = 1000$ modeled genes from the Perturb-seq screen by Replogle et al. (22). This plot complements Fig. 4B, where only genes assigned to clusters are shown for visual clarity. Clusters indicate those analyzed in the enrichment analysis. The matrix is transposed into graph adjacency orientation, so that entry (a, b) corresponds to the direct causal effect of gene a on gene b. Tikhonov regularization: $\lambda = 0.01$ (see Materials and Methods).

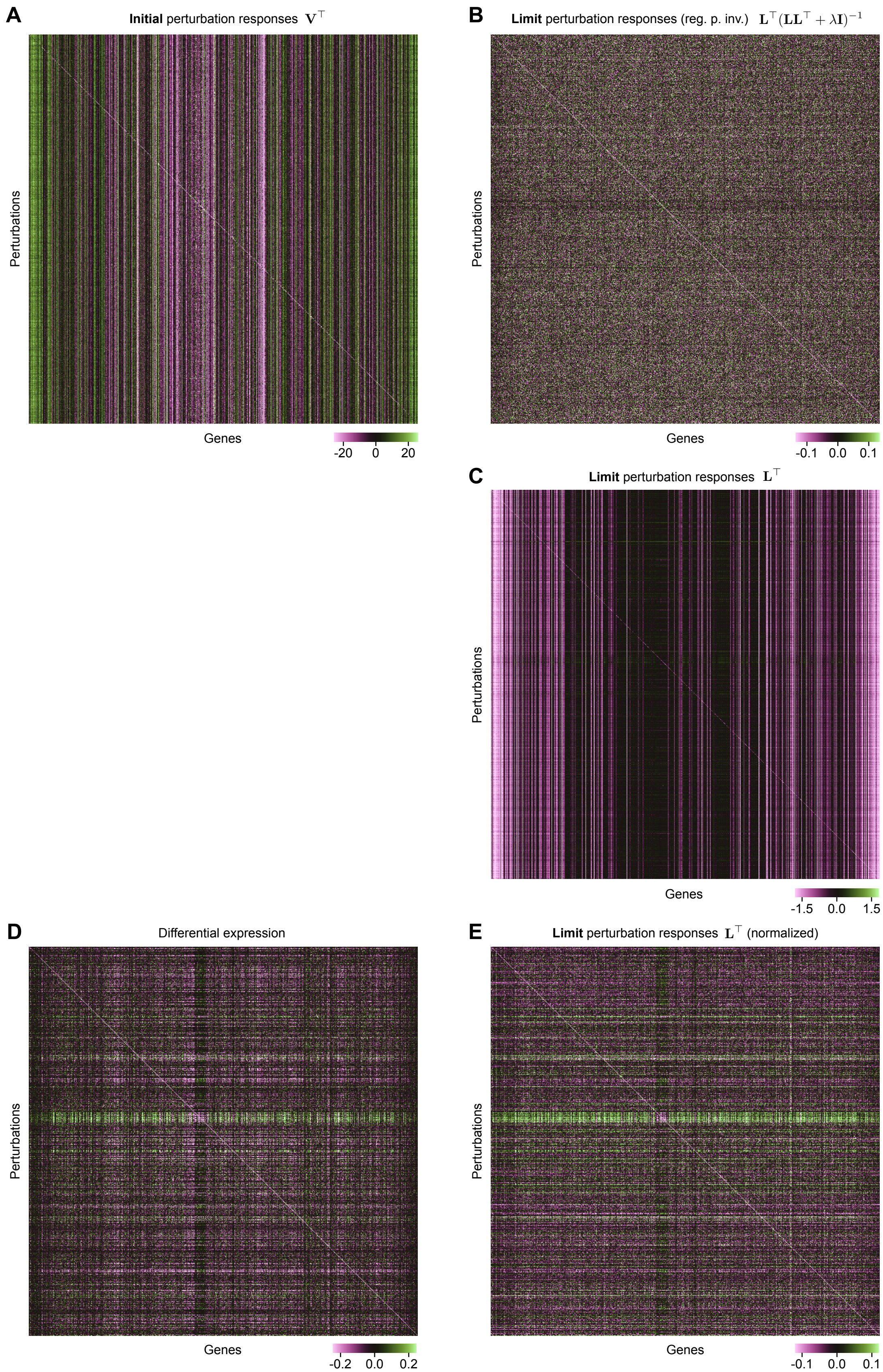

Fig. S10. CLIPR components of the causal effects $\widehat{\mathbf{A}}_\lambda$ in Fig. S9. This plot complements Fig. 4C, where only genes assigned to clusters are shown. (**A**) Initial perturbation responses (Eq. 5). (**B**) Tikhonov-regularized pseudoinverse of the limit perturbation responses. The CLIPR matrix $\widehat{\mathbf{A}}_\lambda$ is equal to the (negative) product of (**A**, transposed) and (**B**). (**C**) Limit perturbation responses (Eq. 6). (**D**) Differential expressions observed in the data. (**E**) Limit perturbation responses, normalized by passing values through the Poisson rate functions μ_g, then applying the same library size standardization and log transformation used to preprocess the data. (**E**) corresponds closely to (**D**). Matrices are transposed with perturbation indices on the first axis. Perturbation on row g targets the gene on column g. Tikhonov regularization: $\lambda = 0.01$ (see Materials and Methods).

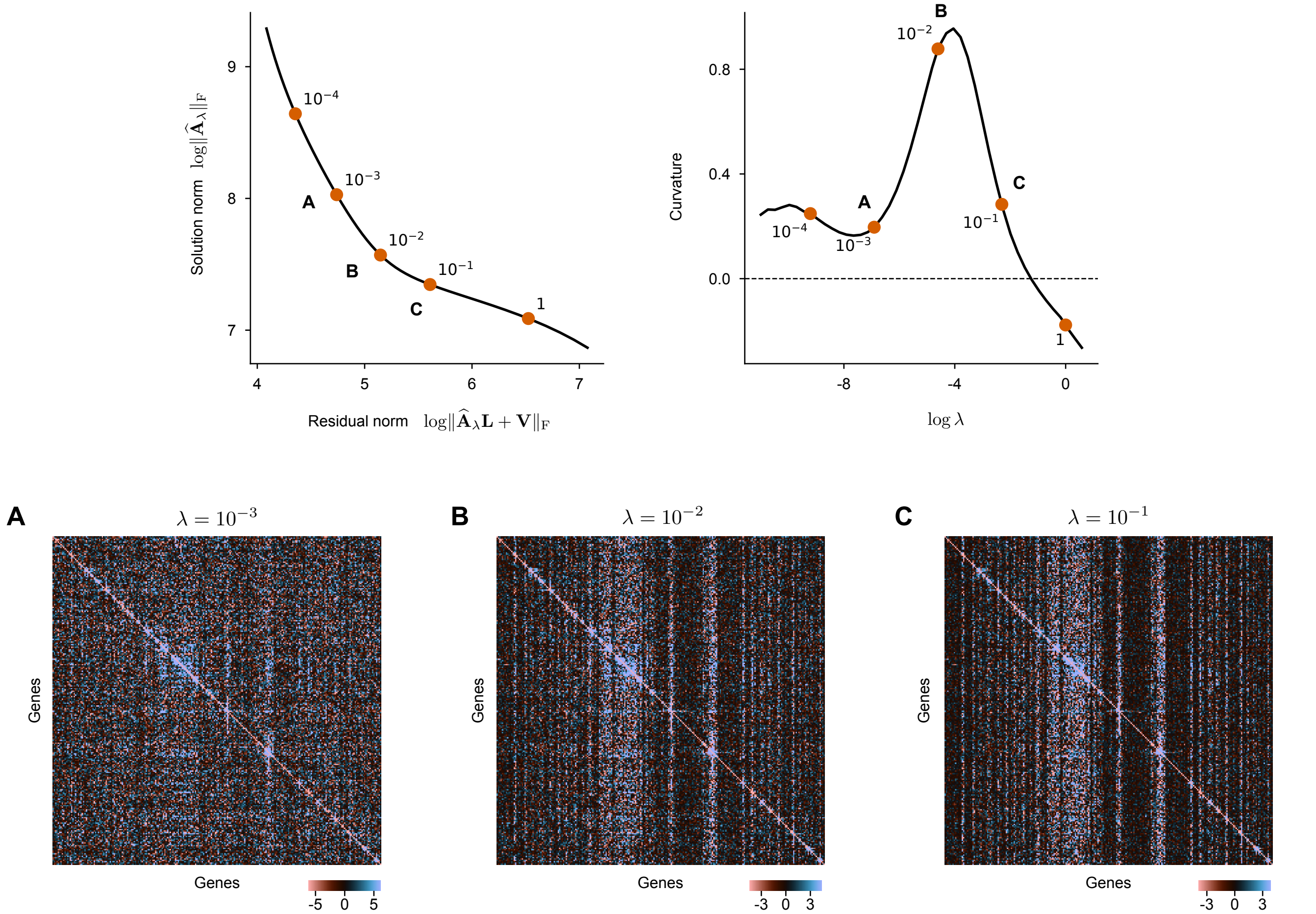

Fig. S11. Selecting the CLIPR regularization strength λ without cross-validation. **Top row:** Left panel plots the norm of the regularized CLIPR estimate $\widehat{\mathbf{A}}_\lambda$ against the norm of its residual $\widehat{\mathbf{A}}_\lambda \mathbf{L} + \mathbf{V}$ for the system of equations in Eq. 17, on a log-log scale, for the CLIPR analysis of Fig. 4. Right panel shows the curvature of the solution-norm versus residual-norm curve in the left panel. We select the regularization level that corresponds to the point of maximum curvature (or bend) of this L-curve (20), here $\lambda = 10^{-2}$. **Bottom row:** Regularized CLIPR estimate $\widehat{\mathbf{A}}_\lambda$ (**A**) below the selected λ, with insufficient regularization ($\lambda = 10^{-3}$), (**B**) at the selected value ($\lambda = 10^{-2}$), and (**C**) above it ($\lambda = 10^{-1}$). Beyond the selected regularization strength, the estimated matrix changes little visually. Top row plots sweep λ over the geometric sequence $\boldsymbol{\lambda} = [10^{-5}, \dots, 3] \in \mathbb{R}^{50}$. For visual clarity, matrices only show the 247 genes within clusters identified in the enrichment analysis (as for Fig. 4B and C).

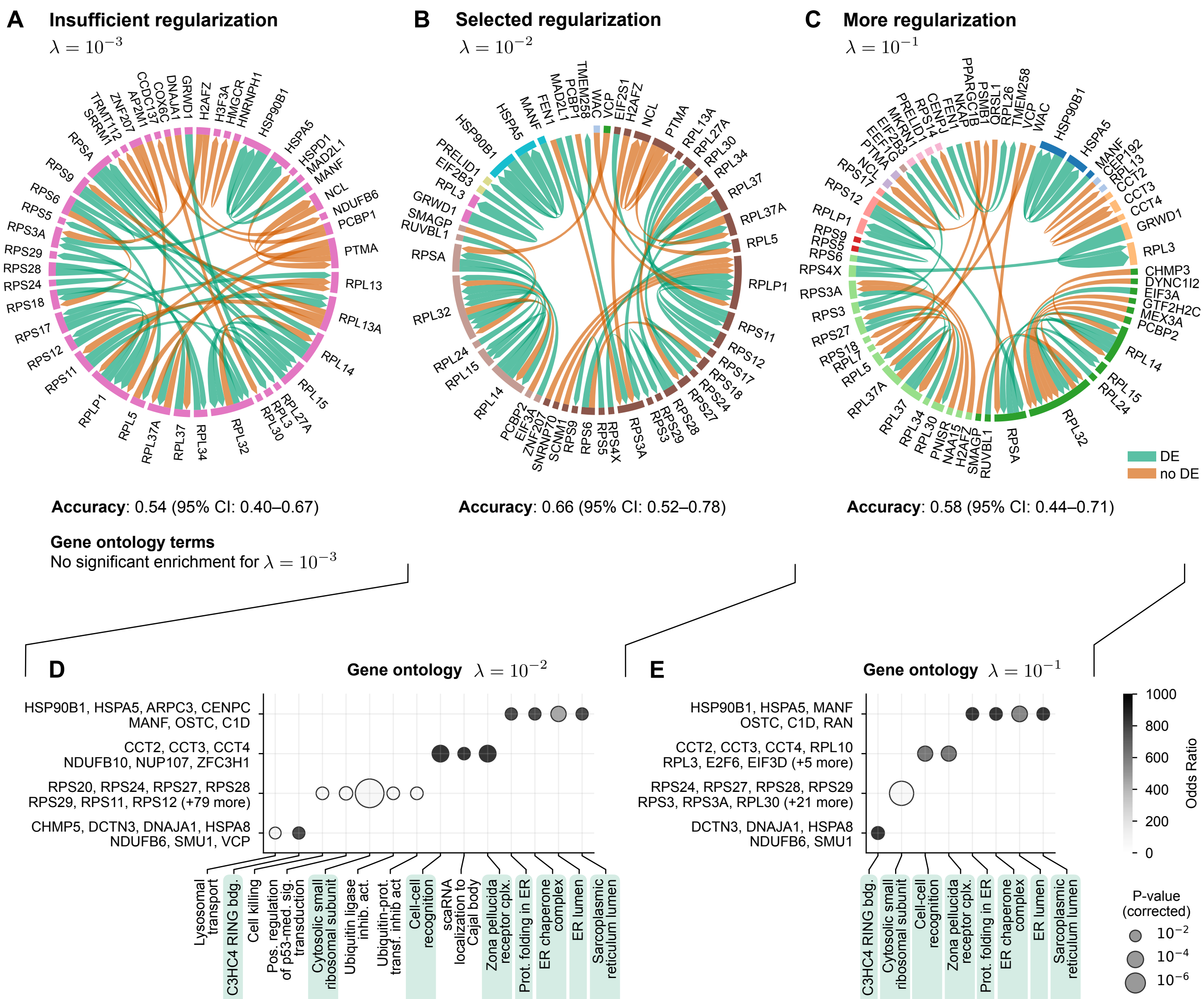

Fig. S12. Sensitivity of LCD-CLIPR to regularization strength λ for the genome-wide Perturb-seq screen. Complements Fig. 4 by showing how the λ values from Fig. S11 affect downstream analysis of the causal structure $\widehat{\mathbf{A}}_\lambda$. **Top row:** Top 50 predicted causal effects at (**A**) insufficient regularization ($\lambda = 10^{-3}$), (**B**) the selected value ($\lambda = 10^{-2}$), and (**C**) above it ($\lambda = 10^{-1}$). Edges are green where the predicted effect is validated by observed DE, and red otherwise. Gene colors denote hierarchical clusterings as described in Materials and Methods (cutting height fixed throughout), with no shared meaning across (**A–C**). **Bottom row:** Gene ontology enrichment analysis of clusters from the full causal matrices $\widehat{\mathbf{A}}_\lambda$ of (**A–C**). Only clusters with at least one enriched term are shown. Under insufficient regularization (**A**), clustering yielded no significant enrichment and lacked visible clusters (cf. Fig. S11A). At the selected (**B**) and higher (**C**) regularization, recovered clusters and enriched terms show substantial overlap. Clusters that show significant enrichment match up to individual genes. All terms enriched for $\lambda = 10^{-1}$ (**E**) are also enriched for $\lambda = 10^{-2}$ (**D**). Shared terms are highlighted in green. **B** and **D** equal Figs. 4D and 4A (top), respectively.

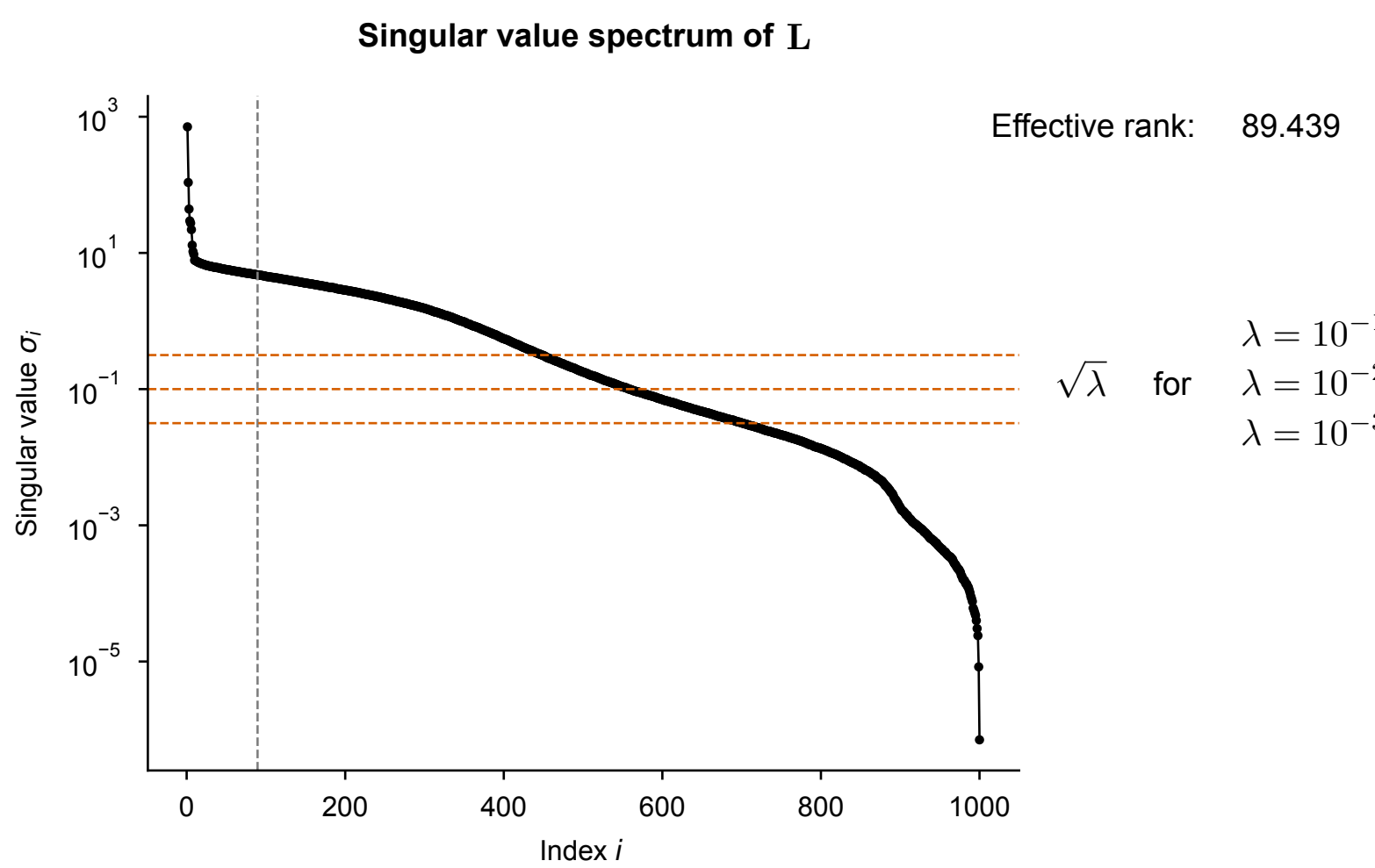

Fig. S13. Spectral analysis of the limit perturbation response matrix $\mathbf{L}$. Singular values $\sigma_1 \geq \cdots \geq \sigma_d$ of the limit response matrix $\mathbf{L}$ used to compute the CLIPR matrix $\widehat{\mathbf{A}}_\lambda$ for the genome-wide Perturb-seq screen in Fig. 4. Vertical line marks the entropy-based effective rank ($\approx$ 89.439) (23). Horizontal lines mark $\sqrt{\lambda}$ for the regularization values studied in Figs. S11 and S12. The Tikhonov-regularized estimate $\widehat{\mathbf{A}}_\lambda$ retains directions with $\sigma_i \gg \sqrt{\lambda}$ and shrinks those with $\sigma_i \ll \sqrt{\lambda}$ toward zero. Directions with small σ_i are weakly constrained by $\mathbf{L}$, so $\widehat{\mathbf{A}}_\lambda$ is shrunk towards zero there. Small entries therefore need not indicate absent regulation.

Algorithm 1 LCD inference

Input: count data $\mathbf{Y}_q$ for control condition q_0 and perturbations $q_1, \ldots, q_k$

Inference stage 1: measurement model

Initialize noise likelihood parameters $\boldsymbol{\pi}$
Initialize prior parameters $\boldsymbol{\phi}_q$ for each condition $q_0, \ldots, q_k$
while not converged **do**
    Randomly select condition q from $q_0, \ldots, q_k$
    Draw M samples from prior $\mathbf{x} \sim p(\mathbf{x}; \boldsymbol{\phi}_q)$ with $\mathbf{x} = g(\boldsymbol{\epsilon}, \boldsymbol{\phi}_q)$ and $\boldsymbol{\epsilon} \sim \mathcal{N}(\mathbf{0}, \mathbf{I})$ to compute loss
    Update $\boldsymbol{\phi}_q$ and $\boldsymbol{\pi}$ with optimizer step

$$\Delta\boldsymbol{\phi}_q \propto -\nabla_{\boldsymbol{\phi}_q}\mathcal{L}_{\mathrm{IP}}(\mathbf{Y}_q, \boldsymbol{\phi}_q, \boldsymbol{\pi}) \qquad \Delta\boldsymbol{\pi} \propto -\nabla_{\boldsymbol{\pi}}\mathcal{L}_{\mathrm{IP}}(\mathbf{Y}_q, \boldsymbol{\phi}_q, \boldsymbol{\pi})$$

Draw final samples $\mathbf{X}_q = \{\mathbf{x}^{(n)}\} \sim p(\mathbf{x}; \boldsymbol{\phi}_q)$ from inferred priors of each condition q
for each condition q **do**
    Standardize $\mathbf{X}_q$ by subtracting mean of $\mathbf{X}_{q_0}$ and dividing by standard deviation of concatenation $\mathbf{X}_{q_0}, \ldots, \mathbf{X}_{q_k}$ (variable-wise)

▷ **inferred** likelihood parameters $\boldsymbol{\pi}$, standardization statistics

Inference stage 2: stationary diffusion

Initialize drift parameters $\boldsymbol{\theta}$
Initialize perturbation embedding $\mathbf{e}_q$ for each $q_1, \ldots, q_k$ and fix $\mathbf{e}_{q_0} = \mathbf{0}$
while not converged **do**
    Randomly select condition q from $q_0, \ldots, q_k$
    Draw batch from $\mathbf{X}_q$ and smoothing levels $\alpha^{(n)}$ for each $\mathbf{x}^{(n)}$ in batch to compute loss
    Update $\boldsymbol{\theta}$ and $\mathbf{e}_q$ with optimizer step

$$\Delta\boldsymbol{\theta} \propto -\nabla_{\boldsymbol{\theta}}\mathcal{L}_{\mathrm{D}}(\mathbf{X}_q, \boldsymbol{\theta}, \mathbf{e}_q, \boldsymbol{\alpha}) \qquad \Delta\mathbf{e}_q \propto -\nabla_{\mathbf{e}_q}\mathcal{L}_{\mathrm{D}}(\mathbf{X}_q, \boldsymbol{\theta}, \mathbf{e}_q, \boldsymbol{\alpha}) \ \text{(when } q \neq q_0\text{)}$$

▷ **inferred** drift parameters $\boldsymbol{\theta}$, perturbation embeddings $\mathbf{e}_{q_1}, \ldots, \mathbf{e}_{q_k}$

Fig. S14. Pseudocode for the ***Inference*** section in Materials and Methods .

Algorithm 2 LCD sampling

Input: query perturbation embedding $\mathbf{e}_{q'}$, model parameters $\boldsymbol{\theta}$ and $\boldsymbol{\pi}$, standardization statistics, step size Δt and steps (same smoothing scales $\boldsymbol{\alpha}$ as during training)

Initialize $\mathbf{x} \sim \mathcal{N}(\mathbf{0}, \mathbf{I})$
for each smoothing scale $\alpha \in \boldsymbol{\alpha}$ in decreasing order **do**
  for each step **do**
    Sample $\boldsymbol{\xi} \sim \mathcal{N}(\mathbf{0}, \mathbf{I})$
    Update $\mathbf{x} \leftarrow \mathbf{x} + f\big(\mathbf{x}; \boldsymbol{\theta}, \mathbf{e}_{q'}, \alpha\big)\alpha^2 \Delta t + \boldsymbol{\xi}\alpha\sqrt{2\Delta t}$

Invert standardization of $\mathbf{x}$ using provided shift and scale statistics
Sample $\mathbf{y} \sim p(\mathbf{y} \,|\, \mathbf{x}; \boldsymbol{\pi})$

$\triangleright$ **return** count sample $\mathbf{y}$ for embedding $\mathbf{e}_{q'}$

Fig. S15. Pseudocode for the *Sampling* section in Materials and Methods .

Algorithm 3 CLIPR

Input: trained perturbation embeddings $\mathbf{e}_{q_1}, \ldots, \mathbf{e}_{q_k}$, drift parameters $\boldsymbol{\theta}$, standardization statistics, Tikhonov regularization λ, step size Δt and steps (same smoothing scales $\boldsymbol{\alpha}$ as during training)

Initialize $\mathbf{V}, \mathbf{L} \in \mathbb{R}^{d \times k}$

Compute limit perturbation responses L by integrating the flow

for each perturbation q_i **do**
  Initialize $\boldsymbol{\ell} = \mathbf{0}$
  for each smoothing scale $\alpha \in \boldsymbol{\alpha}$ in decreasing order **do**
    for each step **do**
      Update $\boldsymbol{\ell} \leftarrow \boldsymbol{\ell} + f\big(\boldsymbol{\ell}; \boldsymbol{\theta}, \mathbf{e}_{q_i}, \alpha\big)\alpha^2 \Delta t$
  Transform $\boldsymbol{\ell}$ based on provided shift and scale standardization statistics (Eq. 16)
  Set i-th column of $\mathbf{L}$ to transformed $\boldsymbol{\ell}$

Compute initial perturbation responses V by evaluating f at control state

Transform $\mathbf{0}$ based on provided shift and scale standardization statistics (Eq. 16)
for each perturbation q_i **do**
  Compute $\mathbf{v} = f(\mathbf{0}; \boldsymbol{\theta}, \mathbf{e}_{q_i}, \min \boldsymbol{\alpha})$ ($\triangleright$ condition on smallest α)
  Transform $\mathbf{v}$ based on provided shift and scale standardization statistics (Eq. 16)
  Set i-th column of $\mathbf{V}$ to transformed $\mathbf{v}$

Compute $\widehat{\mathbf{A}}_\lambda = -\mathbf{V}\mathbf{L}^\top \big(\mathbf{L}\mathbf{L}^\top + \lambda \mathbf{I}\big)^{-1}$

$\triangleright$ **return** CLIPR matrix $\widehat{\mathbf{A}}_\lambda$

Fig. S16. Pseudocode for the *CLIPR* section in Materials and Methods .

Table S1. Perturbation statistics of all datasets after preprocessing and gene selection.

Dataset	Perturbation	Cell line	Cells	Genes	Median cells per perturb.	One-gene perturbs.	Two-gene perturbs.	Genes perturbed
Wessels et al. (7)	Loss-of-function (knockdown; CRISPR-Cas13d)	THP-1	23,722	1000	175	5	104	25
Norman et al. (6)	Gain-of-function (overexpression; CRISPRa)	K562	96,804	1000	338	98	110	98
Replogle et al. (22)	Loss-of-function (knockdown; CRISPRi)	K562	209,462	1000	165	1000	–	1000

Table S2. Genetic interaction metrics following Norman et al. (6).

GI metric	Symbol	Definition
Magnitude	$\mathrm{GI}_{\mathrm{mag}}$	$\sqrt{c_a^2 + c_b^2}$
Similarity	$\mathrm{GI}_{\mathrm{sim}}$	$\mathrm{dcorr}([\boldsymbol{\delta}_a, \boldsymbol{\delta}_b], \boldsymbol{\delta}_{a,b})$
Linear fit	$\mathrm{GI}_{\mathrm{fit}}$	$\mathrm{dcorr}(\boldsymbol{\delta}_{a,b}, c_a \boldsymbol{\delta}_a + c_b \boldsymbol{\delta}_b)$
Equality of contribution	$\mathrm{GI}_{\mathrm{eq}}$	$\frac{\min\{\mathrm{dcorr}(\boldsymbol{\delta}_a, \boldsymbol{\delta}_{a,b}), \mathrm{dcorr}(\boldsymbol{\delta}_b, \boldsymbol{\delta}_{a,b})\}}{\max\{\mathrm{dcorr}(\boldsymbol{\delta}_a, \boldsymbol{\delta}_{a,b}), \mathrm{dcorr}(\boldsymbol{\delta}_b, \boldsymbol{\delta}_{a,b})\}}$

Table S3. Categories of genetic interactions based on the metrics in Table S2 following Norman et al. (6).

Category	Criterion	Description
Suppression	$\mathrm{GI}_{\mathrm{mag}} < 0.97$	Magnitude of double is smaller than expected from individual singles
Additivity	$0.97 < \mathrm{GI}_{\mathrm{mag}} < 1.34$	Magnitude of double is approximately equal to combination of singles
Synergy	$1.34 < \mathrm{GI}_{\mathrm{mag}}$	Magnitude of double is larger than expected from individual singles
Epistasis	$\mathrm{GI}_{\mathrm{eq}} < 0.37$	One perturbation dominates the other in explaining the double profile
Redundancy	$0.92 < \mathrm{GI}_{\mathrm{sim}}$	Single profiles strongly explain the profile of the double
Neomorphism	$\mathrm{GI}_{\mathrm{fit}} < 0.86$	Linear model does not provide good fit of the double profile

References

1. B Øksendal, *Stochastic differential equations.* (Springer), (2003).
2. R Penrose, On best approximate solutions of linear matrix equations. *Math. Proc. Camb. Philos. Soc.* **52**, 17–19 (1956).
3. GH Golub, CF Van Loan, *Matrix computations.* (The Johns Hopkins University Press), 4th edition, (2013).
4. AN Tikhonov, VY Arsenin, *Solutions of Ill-posed Problems.* (Winston and Sons), (1977).
5. S Särkkä, A Solin, *Applied stochastic differential equations.* (Cambridge University Press) Vol. 10, (2019).
6. TM Norman, et al., Exploring genetic interaction manifolds constructed from rich single-cell phenotypes. *Science* **365**, 786–793 (2019).
7. HH Wessels, et al., Efficient combinatorial targeting of RNA transcripts in single cells with Cas13 RNA Perturb-seq. *Nat. methods* **20**, 86–94 (2023).
8. P Virtanen, et al., SciPy 1.0: Fundamental Algorithms for Scientific Computing in Python. *Nat. Methods* **17**, 261–272 (2020).
9. GJ Székely, ML Rizzo, NK Bakirov, Measuring and testing dependence by correlation of distances. *The Annals Stat.* **35**, 2769–2794 (2007).
10. Y Roohani, K Huang, J Leskovec, Predicting transcriptional outcomes of novel multigene perturbations with GEARS. *Nat. Biotechnol.* **42**, 927–935 (2024).
11. A Gretton, KM Borgwardt, MJ Rasch, B Schölkopf, A Smola, A kernel two-sample test. *The journal machine learning research* **13**, 723–773 (2012).
12. M Lotfollahi, et al., Predicting cellular responses to complex perturbations in high-throughput screens. *Mol. systems biology* **19**, e11517 (2023).
13. T Gaudelet, et al., Season combinatorial intervention predictions with Salt & Peper. *arXiv preprint arXiv:2404.16907* (2024).
14. W Zellinger, T Grubinger, E Lughofer, T Natschläger, S Saminger-Platz, Central moment discrepancy (CMD) for domain-invariant representation learning in *International Conference on Learning Representations.* (2017).
15. D Kingma, J Ba, Adam: A method for stochastic optimization in *International Conference on Learning Representations.* (2015).
16. C Bunne, et al., Learning single-cell perturbation responses using neural optimal transport. *Nat. methods* **20**, 1759–1768 (2023).
17. T Zhang, et al., cfDiffusion: diffusion-based efficient generation of high quality scRNA-seq data with classifier-free guidance. *Briefings Bioinforma.* **26**, bbaf071 (2025).
18. A Liberzon, et al., Molecular signatures database (MSigDB) 3.0. *Bioinformatics* **27**, 1739–1740 (2011) Version: 2025.1.Hs.
19. S Brin, R Motwani, JD Ullman, S Tsur, Dynamic itemset counting and implication rules for market basket data in *Proceedings of the 1997 ACM SIGMOD international conference on Management of data.* pp. 255–264 (1997).
20. PC Hansen, DP O'Leary, The use of the L-curve in the regularization of discrete ill-posed problems. *SIAM J. on Sci. Comput.* **14**, 1487–1503 (1993).
21. Y Song, et al., Score-based generative modeling through stochastic differential equations in *International Conference on Learning Representations.* (2021).
22. JM Replogle, et al., Mapping information-rich genotype-phenotype landscapes with genome-scale Perturb-seq. *Cell* **185**, 2559–2575 (2022).
23. O Roy, M Vetterli, The effective rank: A measure of effective dimensionality in *2007 15th European signal processing conference.* (IEEE), pp. 606–610 (2007).
